# Chemistry of trisindolines: natural occurrence, synthesis and bioactivity

**DOI:** 10.1039/d1ra03091d

**Published:** 2021-07-21

**Authors:** First Ambar Wati, Mardi Santoso, Ziad Moussa, Sri Fatmawati, Arif Fadlan, Zaher M. A. Judeh

**Affiliations:** Department of Chemistry, Institut Teknologi Sepuluh Nopember Kampus ITS, Sukolilo Surabaya 60111 Indonesia; Department of Chemistry, College of Science, United Arab Emirates University P. O. Box 15551 Al Ain United Arab Emirates; School of Chemical and Biomedical Engineering, Nanyang Technological University 62 Nanyang Drive, N1.2–B1-14 Singapore 637459 Singapore Zaher@ntu.edu.sg

## Abstract

Heterocyclic nitrogen compounds are privileged structures with many applications in the pharmaceutical and nutraceutical industries since they possess wide bioactivities. Trisindolines are heterocyclic nitrogen compounds consisting of an isatin core bearing two indole moieties. Trisindolines have been synthesized by reacting isatins with indoles using various routes and the yield greatly depends on the catalyst used, reaction conditions, and the substituents on both the isatin and indole moieties. Amongst the synthetic routes, acid-catalyzed condensation reaction between isatins and indoles are the most useful due to high yield, wide scope and short reaction times. Trisindolines are biologically active compounds and show anticancer, antimicrobial, antitubercular, antifungal, anticonvulsant, spermicidal, and antioxidant activities, among others. Trisindolines have not previously been reviewed. Therefore, this review aims to provide a comprehensive account of trisindolines including their natural occurrence, routes of synthesis, and biological activities. It aims to inspire the discovery of lead trisindoline drug candidates for further development.

## Abstract 2

This in-depth review of trisindolines covers their natural occurrence in addition to several routes of synthesis and catalysts used. The biological activities of trisindolines have been discussed with a special emphasis on the structure–activity relationship.

## Graphical abstract

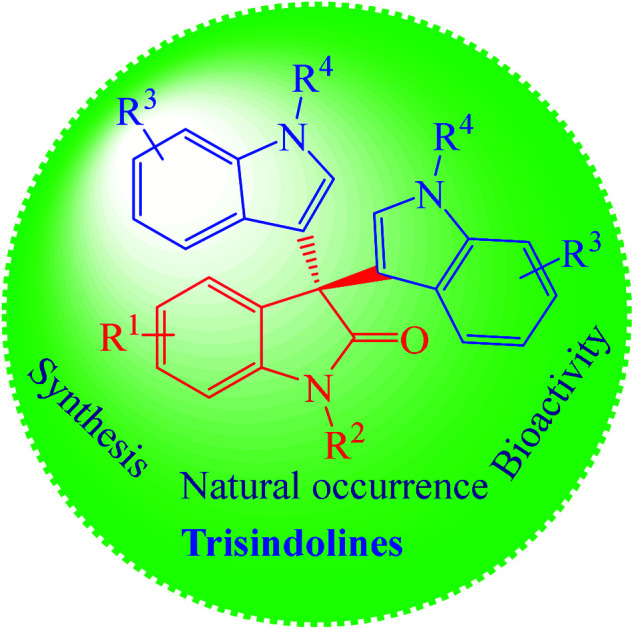

## 1. Introduction

**Fig. 1:**
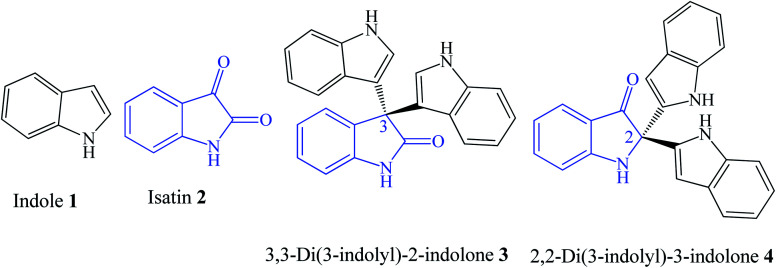
The structures of indole, isatin and the two regioisomers of trisindolines.

Heterocyclic compounds are ubiquitous in nature and possess many bioactivities, making them targets for drug development, health supplements and as highly functional materials. According to the Food and Drug Administration (FDA), 59% of the drug molecules are heterocyclic compounds containing at least one nitrogen atom.^1^ One such compound is the indole 1 (Fig. 1) scaffold with a bicyclic structure incorporating a benzene ring fused to a pyrrole ring.^2^ It is among the top ten nitrogen heterocyclic scaffolds used for constructing drug molecules.^1^ Examples of indole-based drugs include indomethacin, indoxole, and pindolol. Several good reviews covering the chemistry and bioactivity of indoles have been published.^3–5^

Trisindolines are compounds containing two indole units connected to the 1*H*-indol-2,3-dione 2 (also known as isatin) unit either at its C-3 position to form 3,3-di(3-indolyl)-2-indolone 3 or at its C-2 position to form a 2,2-di(3-indolyl)-3-indolone 4 regioisomeric structure (Fig. 1). Since their first discovery,^6^ trisindolines have attracted considerable attention due to their wide biological activities including anticancer,^7–9^ antimicrobial,^10^ antimycobacterial,^11^ antifungal,^10^ anticonvulsant,^10^ α-glucosidase inhibition,^12^ and spermicidal^13^ activities. The 3,3-di(3-indolyl)-2-indolone 3 isomer is far more interesting than the 2,2-di(3-indolyl)-3-indolone 4 isomer due to its higher potency and potential as a drug lead compound.^6^ It is worth noting that the widely occurring 1*H*-indol-2,3-dione 2 (isatin) framework is an indole ring bearing two carbonyl groups at C-2 and C-3 (Fig. 1).^14,15^ Isatin forms a core structure of several biologically active molecules and commercial drugs including Sunitinib, Nintedanib, and Semaxanib.^15,16^ The chemistry and bioactivity of isatins have also been covered in several reviews.^15,17^

To date, no reviews have been published about trisindolines. Hence, this comprehensive review covers the period of 1980–2020 and focuses on the natural occurrence, synthesis, and bioactivities of trisindolines. It highlights the scope, advantages, and limitations of various syntheses of trisindolines. It gives a comprehensive account of the activities reported for trisindolines with a special focus on the structure–activity relationship (SAR) where possible. The review is also meant to be a one-stop reference work for researchers interested in the development of trisindolines as lead drug candidates. Since trisindolines have two isomeric structures, 3,3-di(3-indolyl)-2-indolone 3, and 2,2-di(3-indolyl)-3-indolone 4, we will limit our discussion to the more common and more biologically active 3,3-di(3-indolyl)-2-indolone 3.

## 2. Natural occurrence of trisindolines

**Fig. 2:**
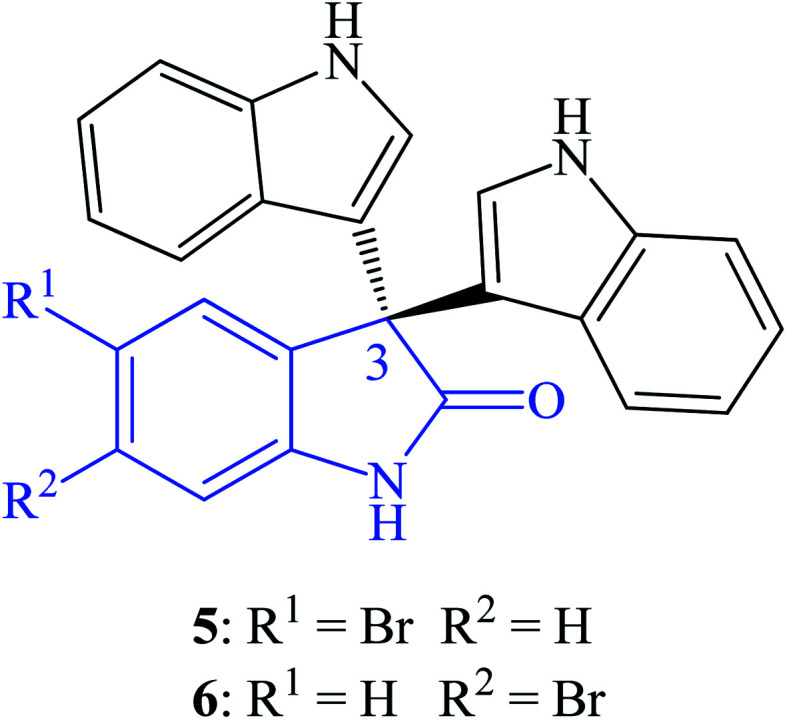
Trisindoline analogues isolated from the red-sea sponge *Callyspongia siphonella*.

Trisindolines were isolated from several natural sources including bacteria, sponges and plants. Trisindoline 3 was firstly isolated from cultured marine bacterium *Vibrio* sp. obtained from the Okinawan marine sponge *Hyrtios altum*.^6^ Trisindolines 3 and 4 were also isolated from the marine sponge *Discodermia calyx*.^18^*Aeromonas* sp., a marine-derived bacterium strain CB101, afforded trisindolines 3 and 4 from its ethyl acetate extract.^19^ Veluri *et al.* (2003) isolated trisindoline 3 and 4 from the ethyl acetate extracts of cultured *Vibrio parahaemolyticus* Bio249's mycelium.^20^ Trisindoline 4 was also isolated from the marine bacterium *Vibrio parahaemolyticus* found in the mucus of stressed fish *Ostracion cubicus* species.^21^ Wang *et al.* (2014) also isolated trisindoline 3 from the ethyl acetate extract of the deep-sea bacterium *Shewanella piezotolerans* WP3.^22^ Trisindoline 3 and its analogues (5-bromo-3,3-di(1*H*-indol-3-yl)indolin-2-one 5 and 6-bromo-3,3-di(1*H*-indol-3-yl)indolin-2-one 6) were isolated from the red-sea sponge *Callyspongia siphonella* (Fig. 2).^23^ Fractionation of the indigo metabolites obtained from the extracts of a recombinant *E. coli* gave trisindoline 3.^9^ Besides sponges and bacteria, the extracts from plant *Isatis costata*, Brassicaseae species, also afforded trisindoline 3.^24,25^ These diverse natural sources gave small amounts of trisindolines. To investigate the bioactivities of trisindolines, practical synthetic routes were developed to obtain them in reasonable amounts and diversify their structures to test their structure–activity relationship (SAR).

## 3. Synthesis of trisindolines

Trisindolines were synthesized (*vide infra*) following several routes. Efficient synthesis of trisindolines depends on the catalyst used, reaction conditions and the reactivity of the indoles and isatins which primarily depends on the position and type (electron donating group or electron withdrawing group) of the substituents on these rings. Additionally, the NH substituents on indoles and isatins rings also impact the efficiency of the reaction. The following sections will highlight these synthetic routes in detail.

**Scheme 1:**
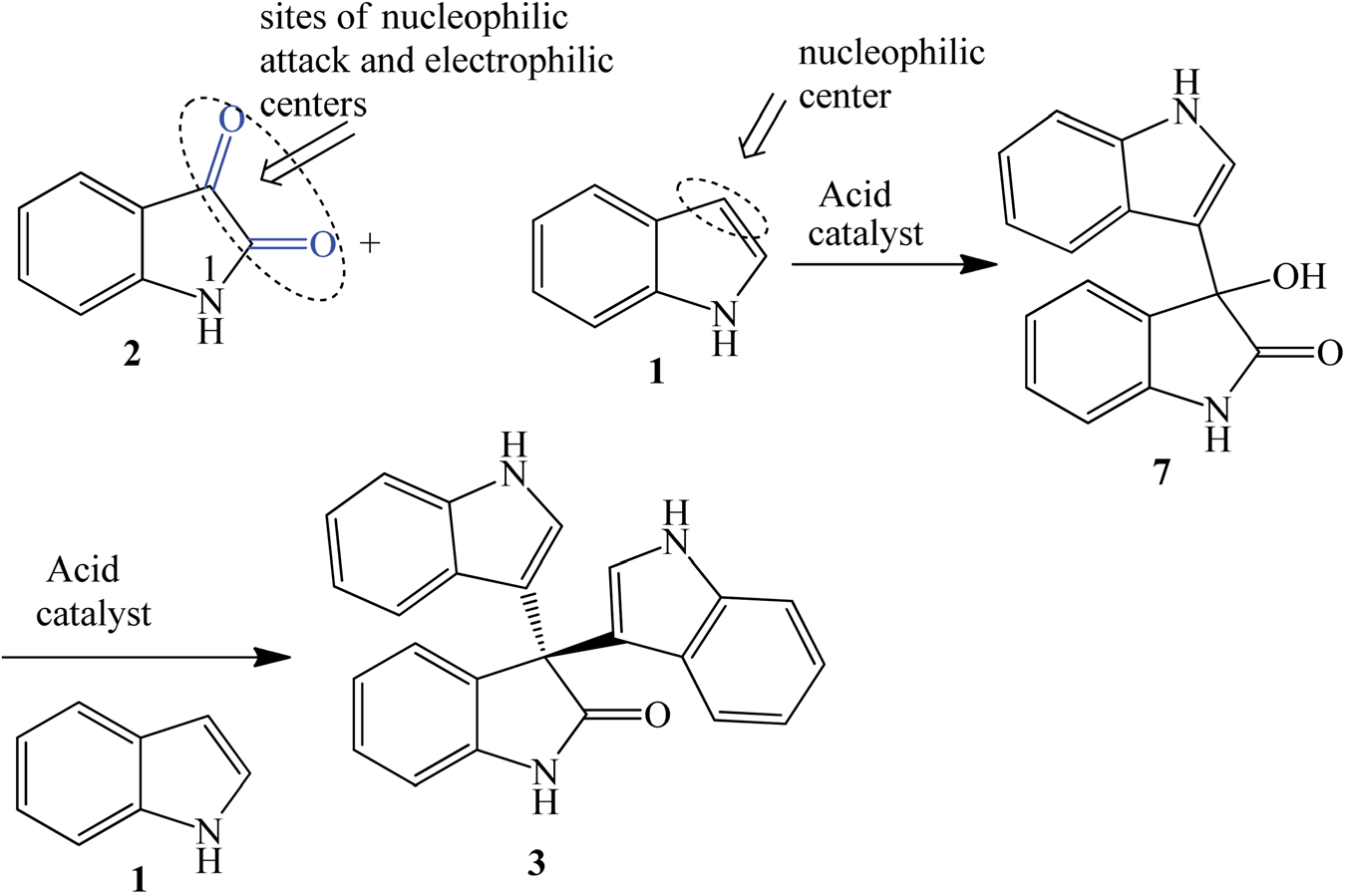
Acid-catalyzed reactions between indole 1 and isatin 2.

**Scheme 2:**
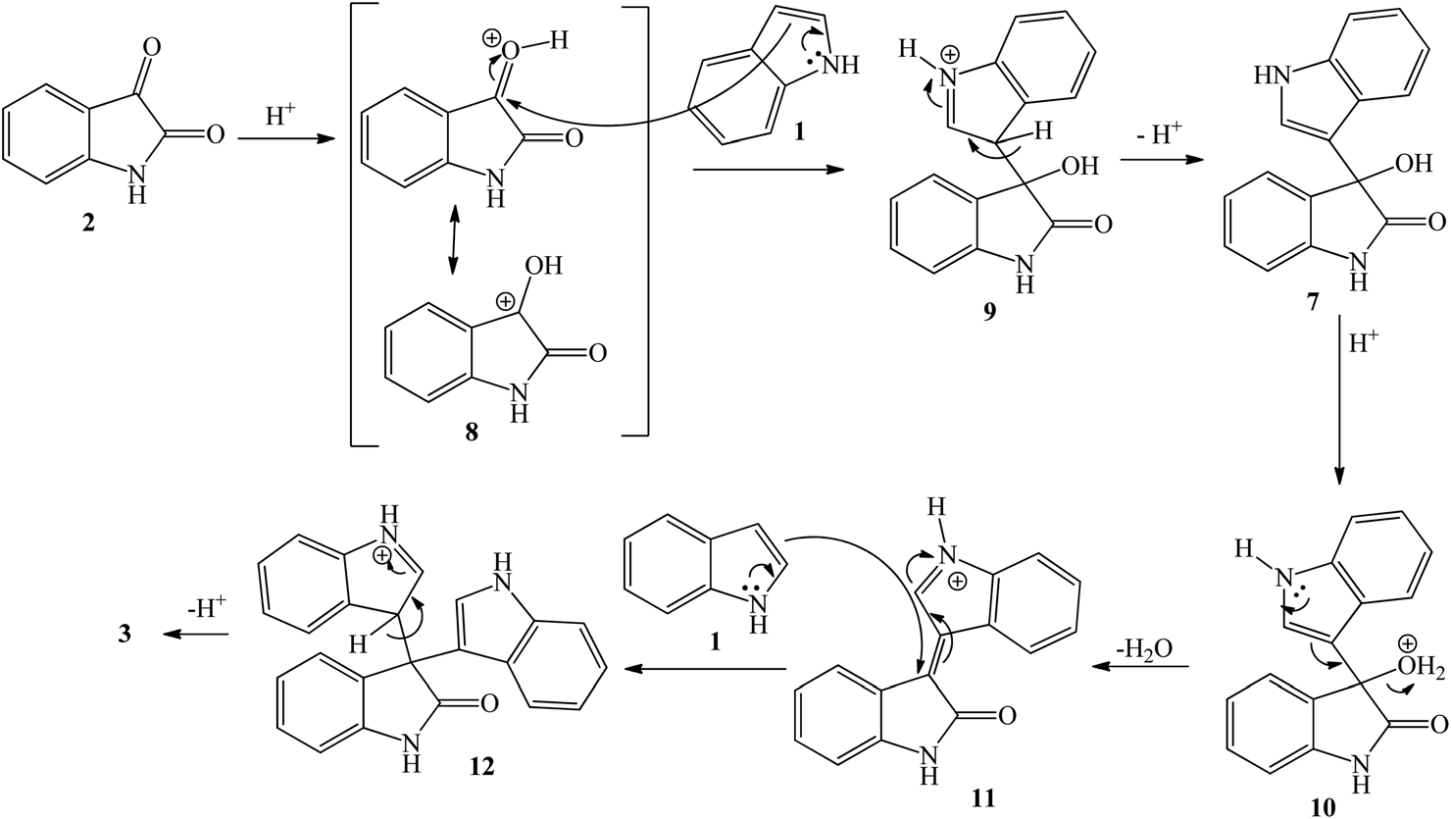
Proposed mechanism of acid-catalyzed formation of trisindoline 3 through reaction between isatin 2 and indole 1.

The synthetic routes based on acid-catalyzed reaction are the most researched and most efficient with respect to yield, selectivity and reaction time.^13^ Trisindolines have also been synthesized in protic solvents and in the complete absence of acid catalysts. Acid-catalyzed indolylation of isatin proceeds through a Friedel–Crafts electrophilic aromatic substitution mechanism. Although the indole itself undergoes faster electrophilic aromatic substitution than benzene, a catalyst is usually required for the indolylation to proceed and give reasonable yields.^26^ The indolylation is made efficient by increasing the nucleophilicity of the indole ring *via* appropriately-positioned substituents and by acid-catalyzed activation of the C-3 carbonyl of isatin.^27^. Mechanistically, the reaction involves two steps where 3-hydroxy-3-indolyl-2-indolone 7 is formed first and then is converted to 3,3-di(3-indolyl)-2-indolone 3 following the addition of a second equivalent of indole 1 (Scheme 1 and 2). Strong acid catalysts directly afford 3,3-diindolyl-2-oxindole 3.^26^

The proposed general mechanism of acid-catalyzed formation of trisindoline 3 through reaction between isatin 2 and indole 1 is shown in Scheme 2. Formation of 3,3-di(3-indolyl)-2-indolone 3 proceeds through a pathway in which the C-3 position of isatin 2 is activated to give intermediate 8 which undergoes nucleophilic attack by the indole 1 to generate 9. Deprotonation of the tertiary alcohol 9 to 3-hydroxy-3-(1*H*-indol-3-yl)indolin-2-one 7 is followed by protonation to form 10. Dehydration of 10 generates α,β-unsaturated iminium ion 11 which is followed by addition of a second molecule of indole 1 and re-aromatization of 12, affording trisindoline 3.

Since many common symmetrical and unsymmetrical trisindolines have been prepared by different routes, we categorized them in Table 1 and 2 and assigned them numbers for easy reference throughout this review. In symmetrical trisindolines, the isatin unit bears the same indole moieties. However, in unsymmetrical trisindolines, the isatin unit bears different indole moieties.

**Table 1:** List of symmetrical trisindolines

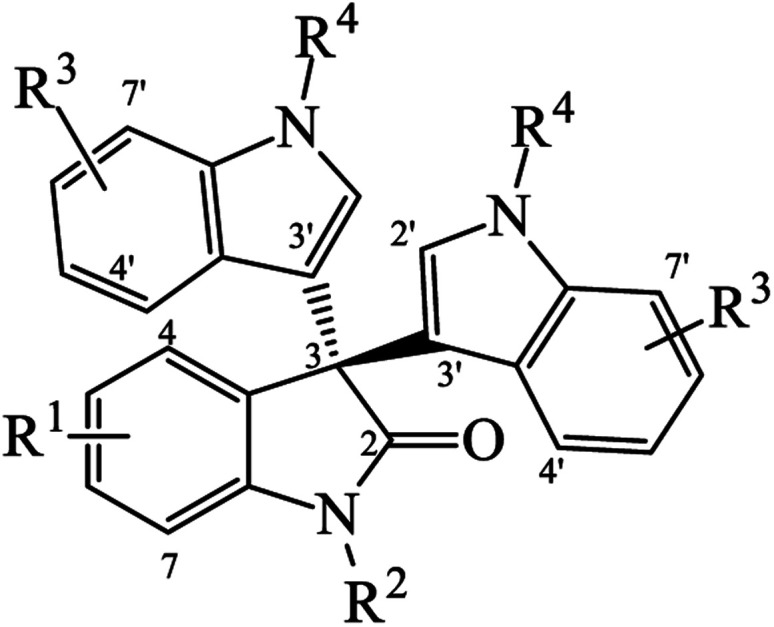							
No. R^1^	R^2^	R^3^	R^4^	No. R^1^	R^2^	R^3^	R^4^
13: 5-Br	4-NO_2_-Bn	5-OH	H	50: H	H	H	2-F-Bn
14: 5-Br	Bn	H	H	51: H	H	H	2-Cl-Bn
15: H	H	5-Br	H	52: H	H	H	3-F-Bn
16: H	H	5-OMe	H	53: H	H	H	4-Br-Bn
17: H	H	H	Me	54: H	H	5-CN	H
18: H	Me	H	H	55: H	H	5-CO_2_Me	H
19: H	Me	H	Me	56: H	H	5-F	H
20: H	Me	5-OMe	H	57: 5-Cl	H	H	Me
21: H	Me	5-Br	H	58: 5-Cl	H	5-F	H
22: H	H	2-Me	H	59: 5-F	H	5-F	H
23: H	Me	2-Me	H	60: 5-F	H	5-OMe	H
24: H	Bn	2-Me	H	61: 5-F	H	H	Me
25: H	Bn	H	H	62: 5-Br	H	5-OMe	H
26: H	Bn	H	Me	63: 5-Br	H	5-F	H
27: 5-NO_2_	H	H	H	64: 5-NO_2_	H	5-F	H
28: 5-NO_2_	H	H	Me	65: 5-NO_2_	H	5-OMe	H
29: 5-Br	H	H	Me	66: H	Me	5-F	H
30: H	H	5-Cl	H	67: H	H	H	Et
31: 5-Cl	H	H	H	68: 5-OMe	H	H	Me
32: 5-F	H	H	H	69: H	H	5-Me	H
33: 5-Me	H	H	H	70: 6-Br	H	H	Me
34: 5-Me	H	5-OMe	H	71: H	H	6-F	H
35: 7-CF_3_	H	H	H	72: H	H	4-F	H
36: 6-Cl	H	H	H	73: H	H	7-F	H
37: 5-OMe	H	H	H	74: H	H	5-Br	Et
38: 5-OMe	H	5-OMe	H	75: H	H	2-Me, 5-OMe	H
39: 5,7-Me	H	H	H	76: H	H	2-Me	Me
40: 5-Me	H	2-Me	H	77: H	H	2-Me	Et
41: 5-I	H	H	Me	78: H	H	2-Me	*n*-Bu
42: H	H	5-NO_2_	H	79: H	H	2-Ph	Me
43: H	H	7-Me	H	80: H	H	2-Ph	Et
44: H	H	6-Cl	H	81: 7-Br	H	H	Me
45: H	H	H	*n*-Pr	82: 5-Me	H	H	Me
46: H	H	H	*i*-Pr	83: 4-Br,5-Me	H	H	Me
47: H	H	H	*n*-Bu	84: 5-NO_2_	H	2-Me	H
48: H	H	H	*i*-Bu	85: 5-Br	H	2-Me	H
49: H	H	H	Bn	86: 5-Cl	H	5-Br	H
87: 5-Cl	H	2-Me	H	131: H	H	H	Cinnamyl
88: 5-Br	H	5-Br	H	132: H	H	5-Br	Prenyl
89: H	Ac	H	H	133: H	Propargyl	5-Br	Prenyl
90: H	Propargyl	H	H	134: H	H	H	Propargyl
91: 5-Cl	Propargyl	H	H	135: H	Bn	H	Propargyl
92: 5-Cl	Bn	H	H	136: H	Propargyl	H	Propargyl
93: 5,7-Br	H	H	H	137: H	H	2-Me	Propargyl
94: 5,7-Br	Bn	H	H	138: H	Me	2-Ph	H
95: 4-Br	H	H	H	139: 5-Me	H	2-Ph, 5-Cl	H
96: H	H	6-Me	H	140: H	H	2-Ph, 5-Me	H
97: H	H	6-NO_2_	H	141: H	H	2-Ph, 5-CN	H
98: 7-F	H	H	H	142: H	H	2-Bu	H
99: 5-NO_2_	H	5-Br	H	143: H	Et	H	H
100: H	Bn	5-Br	H	144: H	H	4-OMe	H
101: 5-F	H	5-Br	H	145: H	H	6-OMe	H
102: 5-OMe	H	2-Me	H	146: 5-F	H	4-OMe	H
103: 5-OMe	H	5-Br	H	147: 5-F	H	6-OMe	H
104: 5-F	H	2-Me	H	148: 5-F	H	5-Cl	H
105: H	H	2-CO_2_H	H	149: 5-NO_2_	Me	H	H
106: H	H	2-Me, 5-NO_2_	H	150: 5-NO_2_	Bn	H	H
107: H	H	2-Ph	H	151: 5-Br	Me	H	H
108: 5-Cl	H	2-Me, 5-NO_2_	H	152: 5-Br	Et	2-Me	H
109: 5-Cl	H	2-Ph	H	153: H	Allyl	H	H
110: 5-Cl	H	5-CO_2_Me	H	154: H	Allyl	5-Br	H
111: 5-Cl	H	5-OMe	H	155: H	Pr	H	H
112: 5-Cl	H	5-NO_2_	H	156: H	Pr	5-Br	H
113: 5-Cl	H	6-Cl	H	157: 5-Cl	Bn	2-Me	H
114: 5-Cl	H	7-Me	H	158: H	Allyl	2-Me	H
115: 5-Me	H	5-Br	H	159: H	Propargyl	2-Me	H
116: 5-Me	H	5-NO_2_	H	160: H	Pr	2-Me	H
117: 5-Me	H	7-Me	H	161 5-Br	Bn	2-Me	H
118: 5-Me	H	6-Cl	H	162: H	Morpholinomethyl	2-Me	H
119: H	H	2-CO_2_Et	H	163: H	Morpholinomethyl	H	H
120: H	H	5-OBn	H	164: 5-Br	Me	2-Me	H
121: 4-Br	H	H	Me	165: 5-Br	Bn	H	Me
122: H	H	7-Et	H	166: H	H	5-OH	H
123: H	H	2-Me	Boc	167: 5-CN	H	5-CN	H
124: 5-F	H	5-NO_2_	H	168: H	Me	5-Me	H
125: H	H	H	Allyl	169: H	Ts	H	H
126: 5-NO_2_	H	H	Allyl	170: H	Ts	2-Me	H
127: H	H	5-OMe	Allyl	171: H	Ts	5-Me	H
128: 5-Cl	H	5-OMe	Allyl	172: H	H	5-COOH	H
129: 5-Br	H	5-OMe	Allyl	173: H	H	4-OH	H
130: H	CH_2_CO_2_Et	5-OMe	Allyl				

**Table 2:** List of unsymmetrical trisindolines

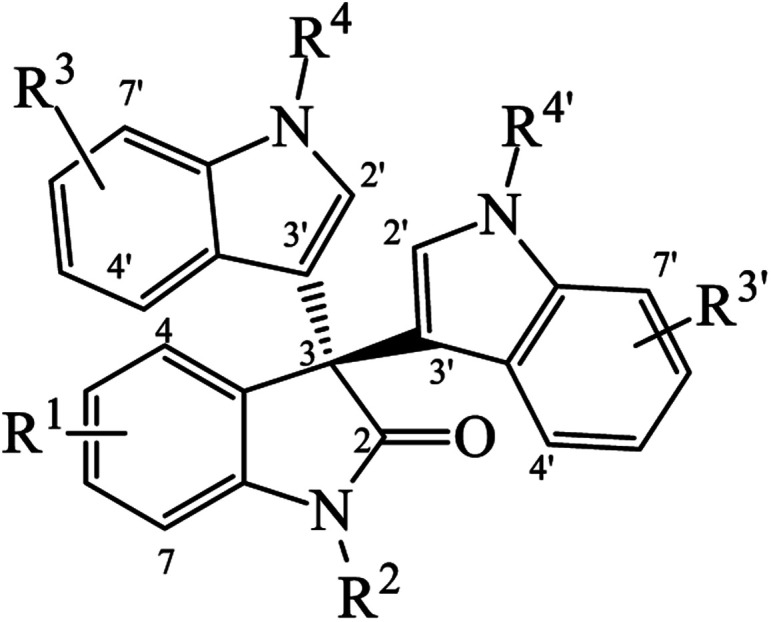					
No. R^1^	R^2^	R^3^	R^3^′	R^4^	R^4^′
174: H	H	H	H	H	Me
175: H	H	5-Br	H	H	H
176: 5-Br	H	5-Br	H	H	H
177: H	H	H	2-Me	H	H
178: H	Bn	H	2-Me	H	H
179: H	Bn	H	5-Br	H	H
180: H	H	5-CN	H	H	H
181: H	Me	H	5-Br	H	H
182: H	H	H	5-Me	H	H
183: H	H	H	6-Me	H	H
184: H	H	H	7-Me	H	H
185: H	H	2-Me	H	H	Me
186: H	H	2-Me	5-Br	H	H
187: H	Bn	H	H	H	Me
188: 5-NO_2_	H	H	5-Cl	H	H
189: H	Allyl	H	2-Me	H	H
190: H	Allyl	5-Br	H	H	H
191: 5-Cl	Allyl	H	5-Br	H	H

In the below sections, the grouping of the catalysts under different headings is not very strict and is meant for easy reference. This is because some catalysts can fall under several groupings.

### 3.1 Synthesis of trisindolines from isatin as a coupling partner

Trisindolines can be synthesized from several coupling partners, the most notable of which is indole 1 and isatin 2. Several reagents and catalysts that promote the coupling process are discussed in the next sub-sections.

#### 3.1.1 Mineral acids-based catalysts

##### 3.1.1.1 Sulfuric acid and related acids catalyzed synthesis

**Scheme 3:**
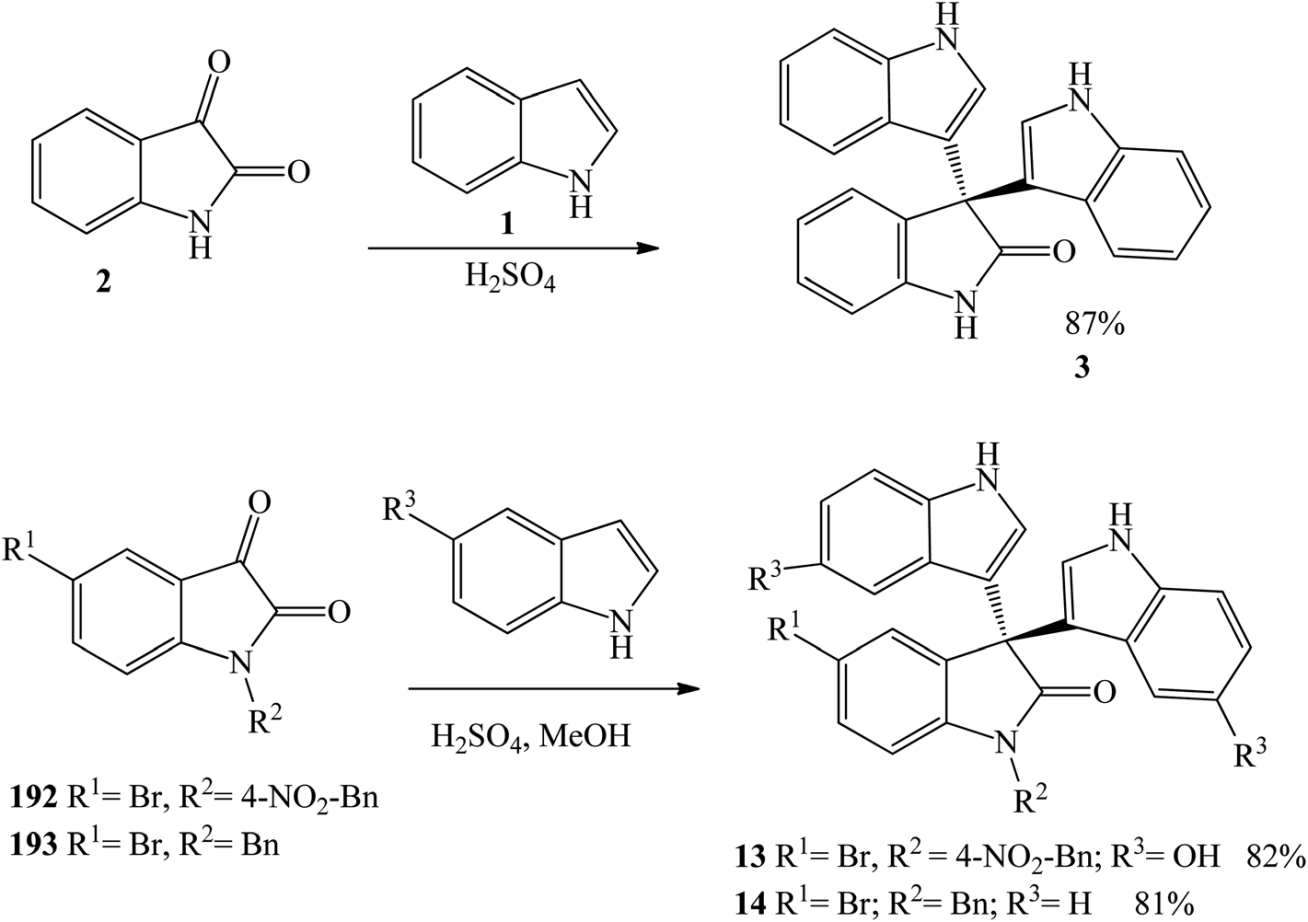
Trisindoline synthesis using sulfuric acid as catalyst.

El-Sayed *et al.* (2015) used sulfuric acid to catalyze the reaction between isatin 2 and indole 1 to prepare trisindoline 3 in 87% yield under reflux conditions in methanol within 2 h.^28^ Additionally, Annuur *et al.* (2018) employed sulfuric acid to react *N*-benzylisatin substrates 192 and 193 under ambient temperature to obtain trisindolines 13 and 14 in 82% and 81% yield, respectively (Scheme 3).^11^

**Scheme 4:**
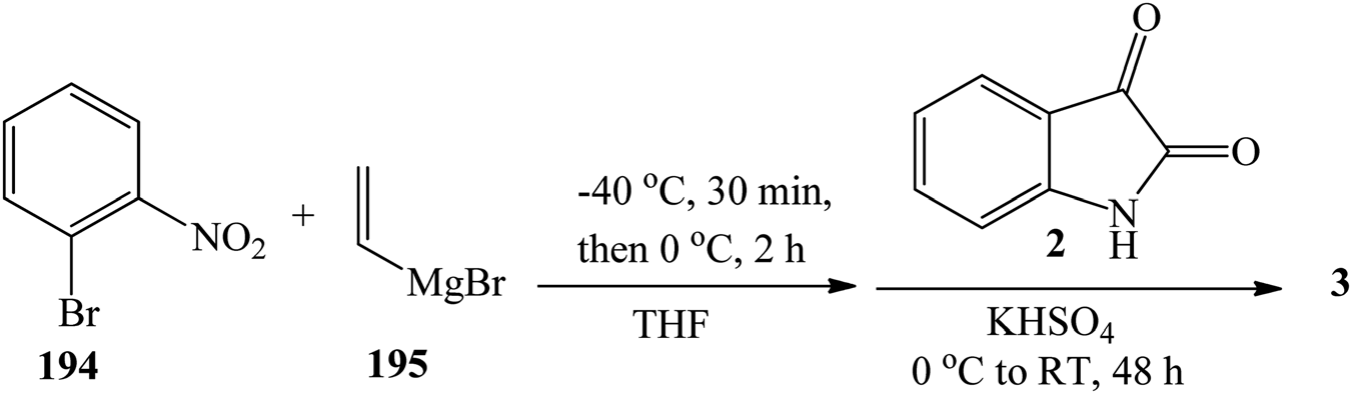
Synthesis of trisindoline 3 by Bartoli indole synthesis.

Bartoli indole synthesis involves reaction of 1-bromo-2-nitrobenzene 194 with vinylmagnesium bromide 195 followed by reaction with isatin 2 in the presence of potassium bisulfate (obtained from KOH + H_2_SO_4_) to give trisindoline 3 in 25% yield. Debromination reaction of the indole ring occurred simultaneously (Scheme 4).^29^ This procedure is time consuming and requires several manipulations resulting in low yields.

##### 3.1.1.2 Tungstic acid and related acids catalyzed synthesis

The monohydrated tungsten trioxide, tungstic acid (H_2_WO_4_, 10 mol%), afforded trisindolines 3 and 15–21 in 88–92% yields within 6 h in ethanol. Remarkably, 5-bromoindole was very reactive with isatin and gave trisindoline 15 in highest 92% yields. The heterogeneous tungstic acid catalyst was recycled by refluxing in dry ethanol for 30 minutes then reused. Other advantages include easy work up and high reactivity under mild conditions especially for NH-substituted indoles and isatins.^30^

The Wells–Dawson heteropoly acid H_6_P_2_W_18_O_62_ is well-known for its super acidity, stability in solution, and its recyclability. Trisindolines 3, 5, 17–19 and 22–29 were successfully synthesized in 86–95% yields using 5 mol% H_6_P_2_W_18_O_62_ in water at 60 °C within 30 minutes. *N*-Alkyl and *N*-benzyl isatins and indoles also reacted to give high yields.^31^

Heteropoly acid silicotungstic acid (H_4_SiW_12_O_40_, 0.1 mol%) successfully catalyzed the synthesis of trisindolines 3, 5, 15, 17, 18, 22–25 and 27–29 in 85–96% yields within 5–70 minutes at room temperature in methanol. However, 5-bromoisatin and 5-bromoindole reacted at a slower rate, giving lower yields.^32^

##### 3.1.1.3 Polyphosphoric acid (PPA) catalyzed synthesis

**Scheme 5:**
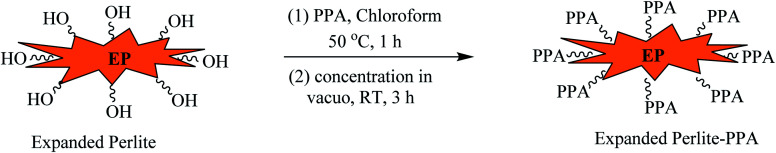
Synthesis of expanded perlite-PPA (EP-PPA).

Polyphosphoric acid (PPA) is a mineral acid catalyst used in several organic transformations. However, owing to its high viscosity, the acid exhibited limited use.^33^ On a different note, perlite is universal name for amorphous volcanic glass that mainly comprises of alumina-silicate. Perlite expands 4–20 times of its original volume once heated to 760–980 °C. Due to its high surface area, low density, and inertness, expanded perlite can be used as solid support for heterogeneous catalysts.^34^ Esmaielpour *et al.* (2017) fabricated expanded perlite-polyphosphoric acid (EP-PPA) (Scheme 5) and evaluated its catalytic activity for bisindolylmethane (BIM) synthesis. The BIMs were obtained in excellent yields within short reaction times. Nevertheless, trisindoline 3 was obtained in 65% yield within 40 minutes.^33^

#### 3.1.2 Organic acids-based catalysts

The synthetic routes based on organic acid-catalyzed reactions to afford 3,3-diindolyl-2-oxindoles are generally selective and give high yields under short reaction time. The next subsections discuss the use of various organic acids in this regard.

##### 3.1.2.1 *p*-Toluenesulfonic acid (*p*-TSA)-catalyzed reactions

**Scheme 6:**
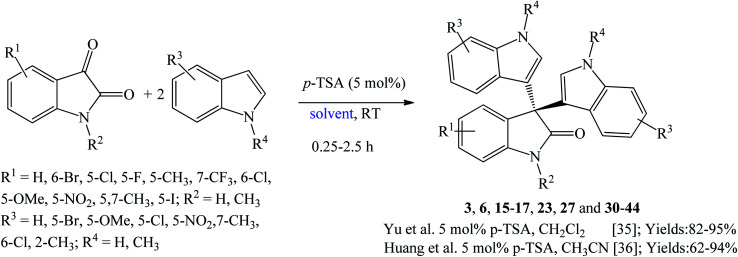
*p*-TSA-catalyzed synthesis of trisindolines 3, 6, 15–17, 23, 27 and 30–44.

*p*-Toluenesulfonic acid (*p*-TSA) is a nontoxic, affordable, safe, and readily available catalyst used frequently in many organic transformations.^35^ Yu *et al.* (2014) prepared a series of trisindolines 3, 16, 23, 27 and 30–41 in 82–95% yields within 10–150 minutes employing 5 mol% *p*-TSA-catalyzed reaction of isatins and indoles in dichloromethane at room temperature (Scheme 6).^35^ Reactions of isatins bearing EDG afforded higher yields under shorter reaction time compared to reactions of isatins bearing EWG. The reaction of electron-rich isatins and electron-rich indoles gave the highest yields within shorter reaction times. Similar reaction results were obtained by Huang *et al.* (2015) (Scheme 6),^36^ when dichloromethane was replaced by acetonitrile where trisindolines 3, 6, 15–17, 31, 33 and 42–44 were obtained in 62–94% yields. *N*-Methylindole reacted smoothly with isatin within 3 h to yield trisindoline 17 in excellent yield (90%). Like the case of Br_2_-catalyzed reactions, *N*-tosylindole did not react even after 24 h.^36^ In comparison, although the yields of the products are similar, reactions in dichloromethane proceeded at a much faster rate than in acetonitrile (less than 1 h *vs.* several hours).

Following that, a series of trisindolines 3, 5, 17, 31, 33 and 45–53 alkylated and benzylated at the indole rings were synthesized using 10 mol% *p*-TSA in dichloromethane *albeit* with moderate yields of 50–76%.^12^

##### 3.1.2.2 Acetic acid-catalyzed reactions

**Scheme 7:**
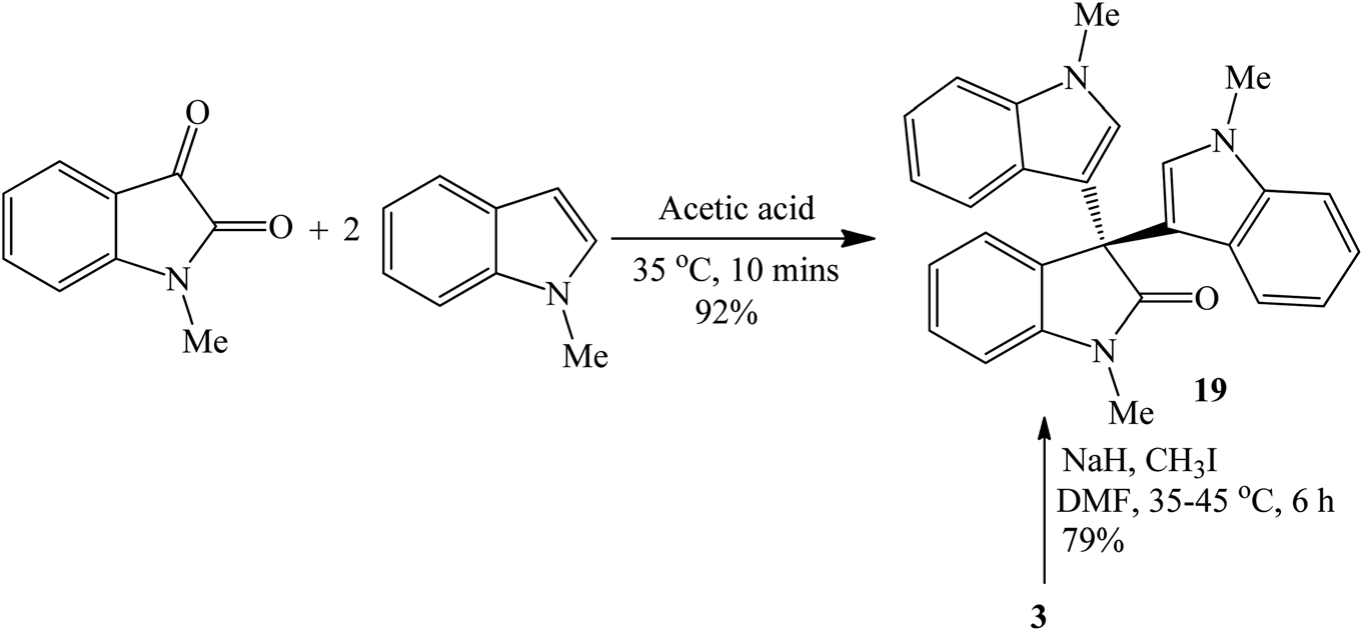
Synthesis of trisindoline 19.

Acetic acid, as both catalyst and solvent, afforded 19 at 35 °C within 10 minutes in 92% yield.^37^ This method is superior (mild, high yield, short reaction time) to the synthesis by alkylation of trisindoline 3 using CH_3_I (3 equivalents) in the presence of NaH in DMF at 35–45 °C which gave only 79% yield (Scheme 7).

##### 3.1.2.3 Succinimide-*N*-sulfonic acid catalyzed reactions

Trisindolines 3 and 22 were successfully synthesized in 96% and 92% yields, respectively, using 5 mol% succinimide-*N*-sulfonic acid in acetonitrile.^38^

##### 3.1.2.4 Sulfamic acid-catalyzed reactions

**Scheme 8:**
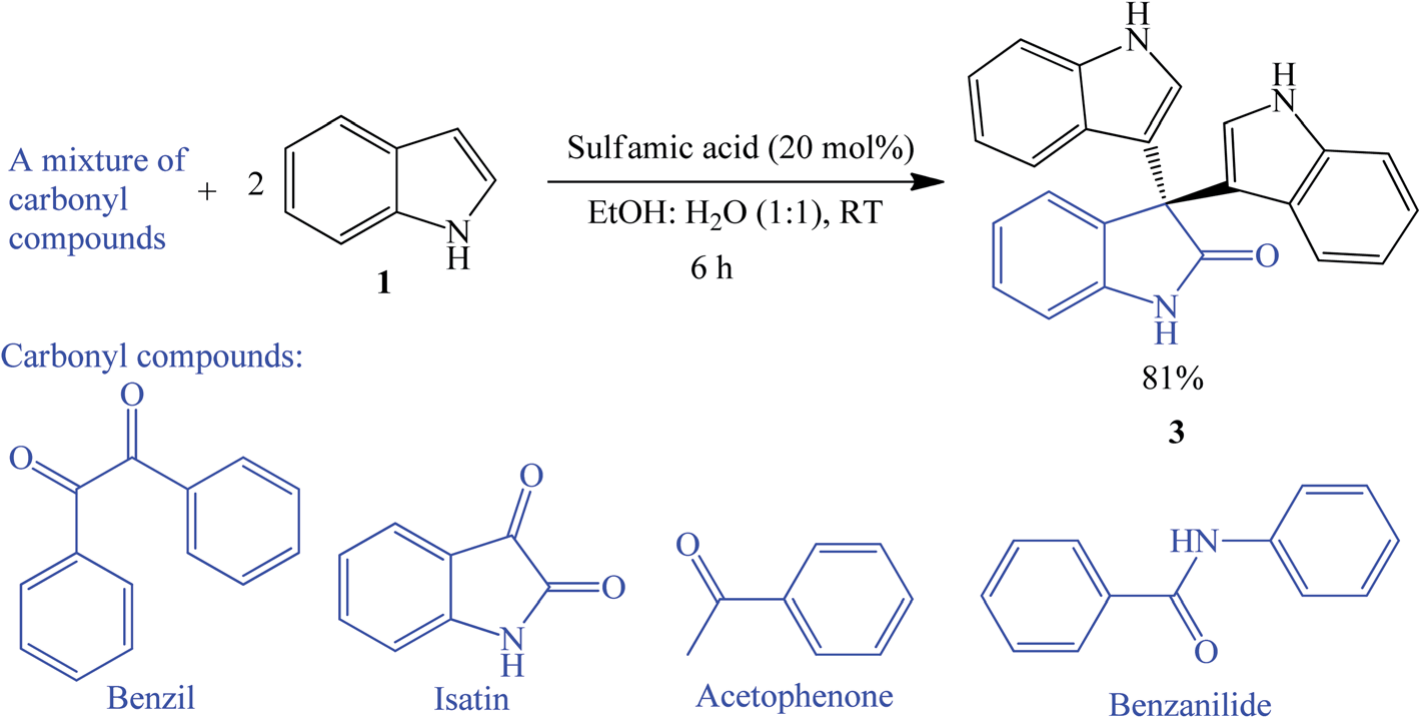
Competitive reaction between indole 1 and carbonyl compounds.

An efficient and simple synthesis of trisindolines 3, 5, 15–18, 20, 27, 29, 31, 32 and 54–66 was disclosed by Brahmachari *et al.* (2014) using sulfamic acid (NH_2_SO_3_H). Reaction between indoles and isatins using 20 mol% sulfamic acid in ethanol : water (1 : 1, v/v) at ambient temperature gave the desired trisindolines in 84–94% yields within 2–7 h. The electron deficient 5-cyanoindole was unreactive. The catalyst was also reused with a slight decrease in efficiency in the third cycle.^39^ In a competitive experiment (Scheme 8), when the indole was treated with several carbonyl compounds including benzil, isatin, acetophenone, and benzanilide, it selectively reacted with isatin to produce trisindoline 3 in 81% yield. Sharma *et al.* (2016) adopted Brahmachari *et al.* method to synthesize 56 in 87% yield.^40^

##### 3.1.2.5 *N*′-isopropylbenzohydrazide hydrochloride-catalyzed reactions

**Fig. 3:**
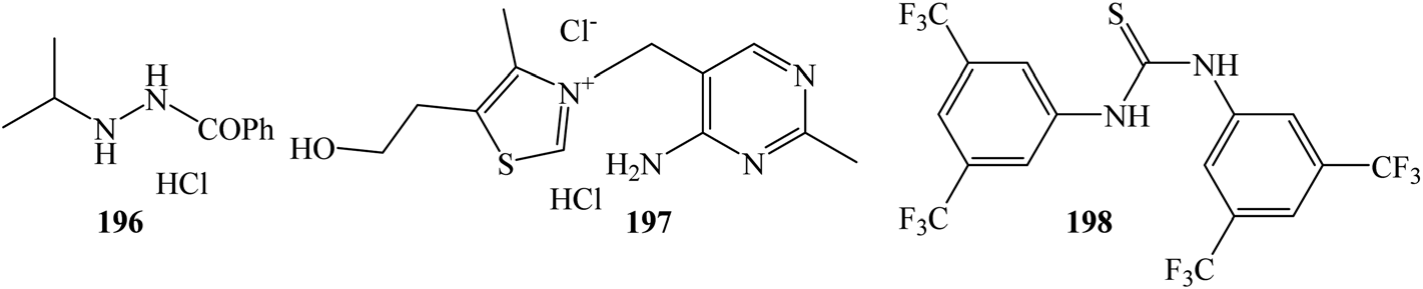
Structures of catalysts 196, 197 and 198.

The Friedel–Crafts reaction between isatin 2 and indole 1 was successfully catalyzed using 10 mol% *N*′-isopropylbenzohydrazide hydrochloride 196 aminocatalyst in methanol at room temperature to afford trisindoline 3 in 72% yield.^41^ No variations of the indoles or isatins were reported (Fig. 3).

##### 3.1.2.6 Thiamine (vitamin B_1_) hydrochloride catalyzed reactions

**Scheme 9:**
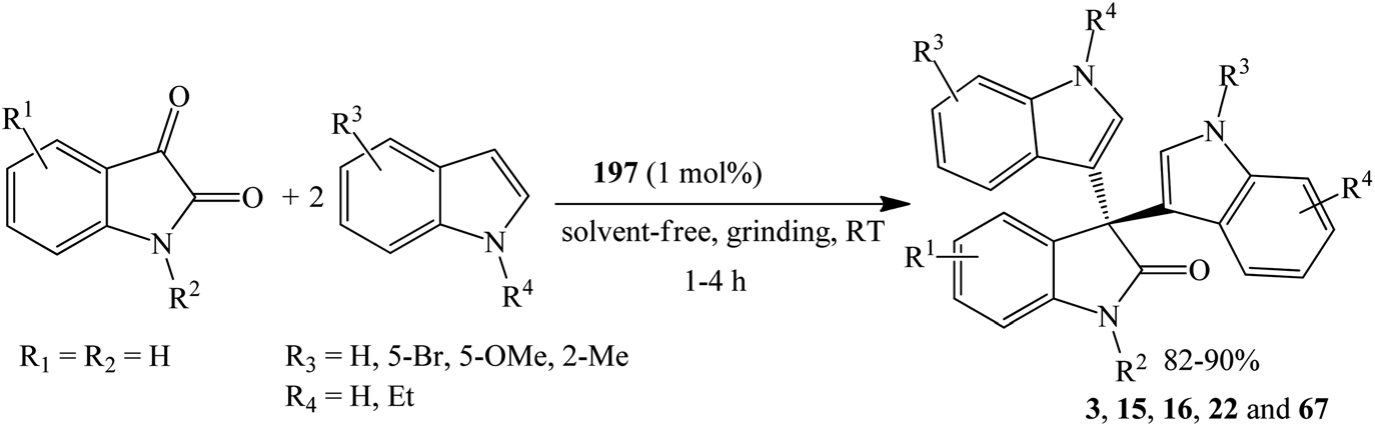
Thiamine hydrochloride 197 catalyzed synthesis of trisindolines 3, 15, 16, 22 and 67.

Mathavan *et al.* (2019) utilized the environmentally benign aminocatalyst thiamine (vitamin B_1_) hydrochloride 197 (1 mol%) to catalyze the solvent-free condensation reaction between indoles and isatins under grinding conditions (Scheme 9). The reaction at ambient temperature gave trisindolines 3, 15, 16, 22 and 67 in yields ranging from 82–90% after 10 h. Under these conditions, 5-methoxyindole and 5-bromoindole as well as *N*-ethylindole gave high yields of the trisindolines 16, 15 and 67. 2-Methylindoles also reacted but gave 82% yield of 22. Notably, the catalytic activity of the recovered catalyst remained high, giving trisindoline 3 in 96% yield in the first cycle and in 78% yield after ten cycles.^42^

##### 3.1.2.7 Dodecylsulphonic acid catalyzed reactions

Hazarika *et al.* (2008) used 10 mol% dodecylsulphonic acid (DCA) as a catalyst and water-solubilising agent to catalyze the reaction of indole 1 and isatin 2 to obtain trisindoline 3 in 87% yield within 20 minutes at room temperature. No other variations of the isatins and indoles were evaluated.^43^

##### 3.1.2.8 Photoacids catalyzed reactions

Photoacids are molecules that become more acidic when absorb light. They can acidify a neutral aqueous solution within very short times (nanoseconds).^44^*N*,*N*′-Bis[3,5-bis(trifluoromethyl)phenyl]thiourea 198 also known as Schreiner's thiourea belongs to photoacid organocatalysts that function as proton donors. Trisindole 32 was synthesized in 79% yield using a blue LEDs-activated Schreiner's thiourea 198 in 1,4-dioxane at room temperature for 18 h.^45^ Under these conditions, but using 370 nm LEDs, trisindolines 3 and 18 were synthesized in 69% and 65% yields, respectively. Unfortunately, the synthesis of trisindoline 32 required 4 equivalents of the indole which is much higher than other routes. This finding reveals that light irradiation plays a prominent role in initiating the reaction.

##### 3.1.2.9 Acidic solvent-catalyzed reactions

**Scheme 10:**
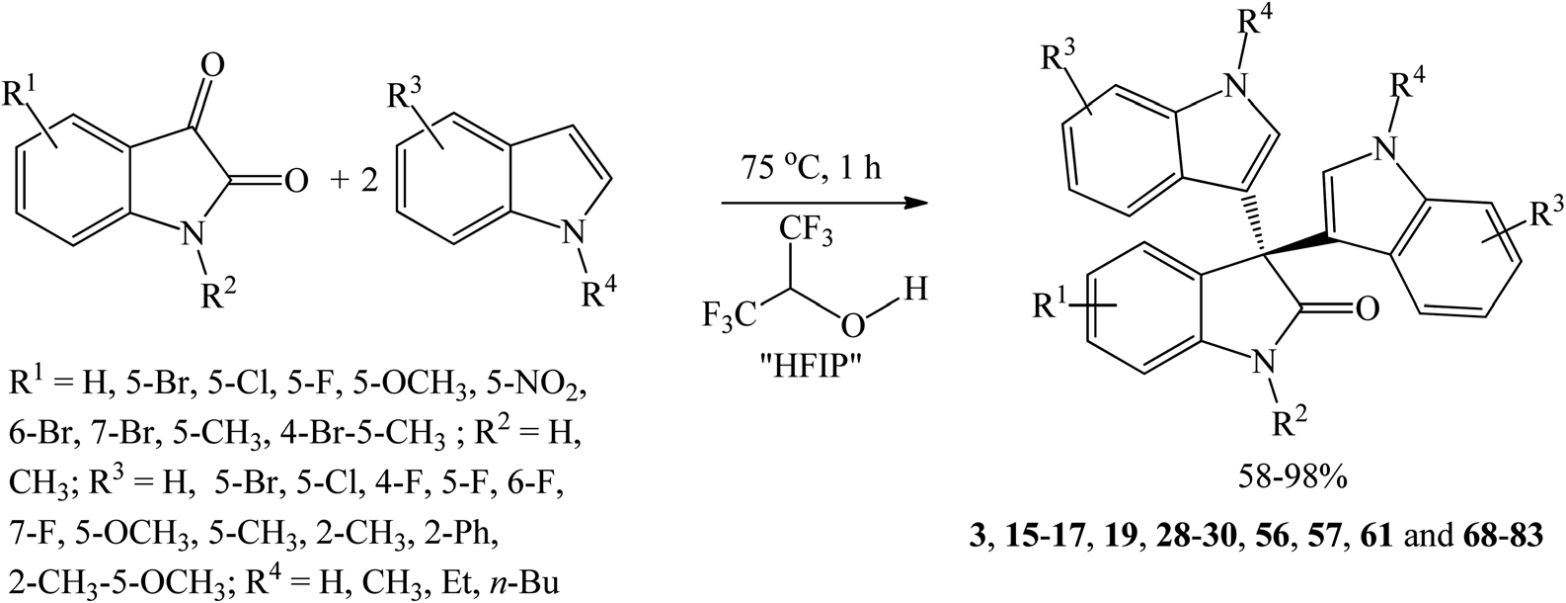
HFIP-catalyzed synthesis of symmetrical trisindolines 3, 15–17, 19, 28–30, 56, 57, 61 and 68–83.

Yuan *et al.* (2020) used hexafluoro-2-propanol (HFIP, p*K*_a_ = 9.3) as a strong H-bond donor solvent to obtain a series of trisindolines 3, 15–17, 19, 28–30, 56, 57, 61 and 68–83 in 58–98% yields (Scheme 10).^46^ HFIP activates the C_3_ carbonyl group of isatin in the first step and is also involved in subsequent proton transfer reactions during the isomerization and dehydration processes of the next two steps. The reaction conditions work well with *N*-alkylated indoles but are ineffective with indoles bearing substituents (*e.g.* methyl, phenyl) on C-2 of the indole ring. Additionally, no desired products were obtained when indole bearing NO_2_ or CN was used as substrate. However, isatin ring tolerates a wide range of substituents such as halogen, nitro, methyl and methoxy substituents.^46^

#### 3.1.3 Heterogeneous and nanoparticle acidic catalysts

##### 3.1.3.1 Amberlyst-15-catalyzed reactions

Amberlyst-15, an acidic cation-exchange resin, is a heterogeneous catalyst that has been used for several organic transformations. Sarrafi *et al.* (2012) reacted various isatins and indoles in the presence of Amberlyst-15 in water (reaction of *N*-benzylisatin using H_2_O : acetone (4 : 1)) at 70 °C for 30 minutes, furnishing 88–95% yields of the trisindolines 3, 5, 17–19, 22–29, 84 and 85. 5-Nitro- and 5-bromo isatins reacted well with either indole or 5-methylindole while derivatization of the NH of the isatin or indole with electron-donating group did not have any significant effects on reaction rate and product yields.^47^ The catalyst showed outstanding reusable activity. Amberlyst-15 was also found to catalyze the electrophilic substitution reaction of 3-methylindole with isatins to afford the corresponding 2,2-diaryloxindole 4 in high yield. This is an added advantage since the number of reports on the reaction of isatin with 3-substituted indoles is scant.^47^

##### 3.1.3.2 Clay-catalyzed reactions

**Scheme 11:**
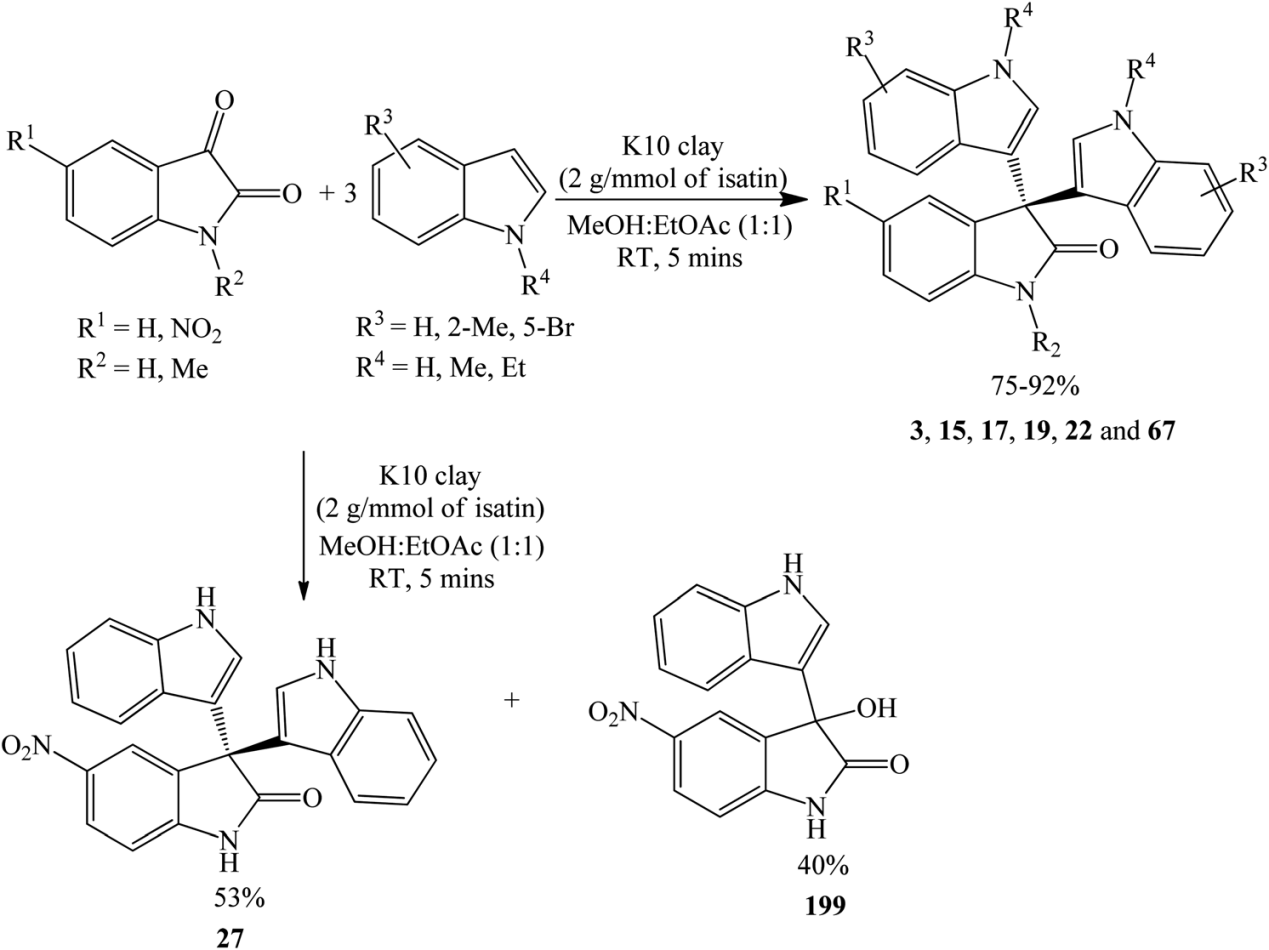
K10 clay-catalyzed synthesis of trisindolines.

Clay is a naturally occurring aluminosilicates solid acid. Modified montmorillonites such as K10 clay and KSF exhibit both Brønsted and Lewis acid properties and are characterized by a large surface area. K10 (250 m^2^ g^−1^) offers a remarkably higher surface area compared to KSF (around 10 m^2^ g^−1^)^48^ and has been shown to catalyze trisindolines formation much faster than KSF. K10 clay was evaluated by Chakrabarty *et al.* (2005) under ambient temperature for 5 minutes by adsorbing a solution of isatin and 3 equivalents of the indole in ethyl acetate : methanol (1 : 1), furnishing the desired products 3, 15, 17, 19, 22 and 67 in moderate to excellent yield (75–92%). While methyl-substituted indole on either C-2 or the *N* atom produced higher yields, *N*-Et substitution decreased the reactivity. The reaction of 5-nitroisatin and indole afforded not only the desired trisindoline 27, but also 199, which was expected as an intermediate product (Scheme 11).^49^ Intermediate product 199 may be used for the synthesis of unsymmetrical trisindolines.

Nikpassand *et al.* (2010) synthesized symmetrical trisindolines 3, 5, 15, 17, 22, 29, 31 and 85–88 and unsymmetrical trisindoline 174, 175 and 176 using KSF-catalyzed one-pot reaction of isatin and indoles in refluxing ethanol within 22–35 minutes. Excellent yields of 85–93% for symmetrical and 88–91% for unsymmetrical trisindolines were obtained. 2-Methylindole enhanced the reactivity and the yield. 5-Br isatins showed enhanced reactivity in some cases compared to their chloro analogues. Unsymmetrical trisindolines 174, 175 and 176 were prepared by initially refluxing equimolar amounts of isatin and indole substrates, followed by adding a second equivalent of a different indole. Without KSF, the reaction was unsuccessful even when heated at reflux for 12 h. The catalyst was easily recovered and reused without significant loss of reactivity.^50^

##### 3.1.3.3 Cellulose sulfuric acid (CSA) catalyzed reactions

Alinezhad *et al.* (2010) conduct solvent-free ecofriendly synthesis of trisindolines by grinding a mixture of isatins and indoles with cellulose sulfuric acid (CSA) catalyst at room temperature. 2-Methylindole reacted more smoothly with isatin within 10 minutes to form 22 in 85% yield in comparison to unsubstituted indole which gave 3 in 88% yield within 2 h.^51^

##### 3.1.3.4 Acidic nano-SiO_2_ catalyzed reactions

**Scheme 12:**
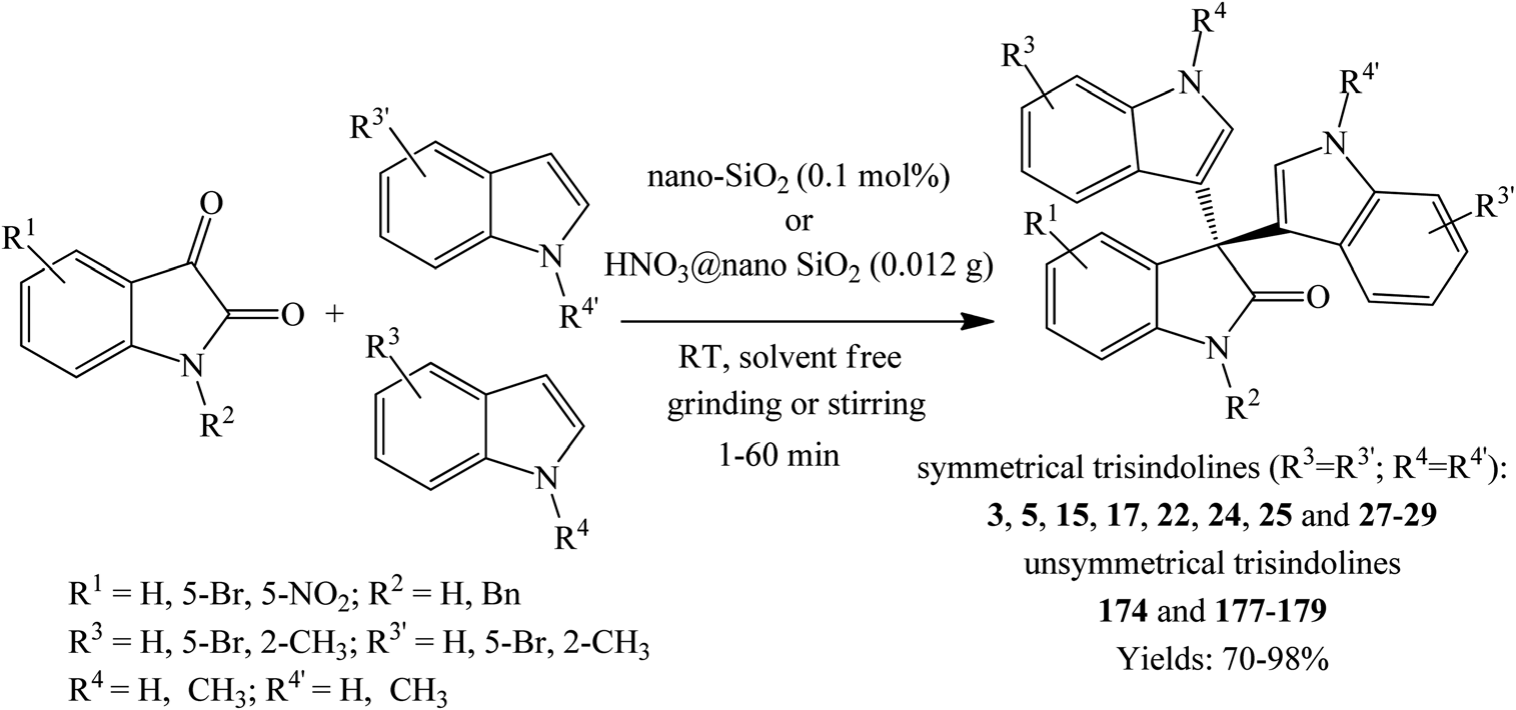
Coupling of isatins and indoles using nano SiO_2_ catalyst and HNO_3_@nano SiO_2_.

Nikoofar and Khalili (2016) conducted one-pot solvent-free condensation reaction of various isatins and indoles in the presence of 0.1 mol% nano-SiO_2_ acidic catalyst under ambient temperature using two different techniques. The stirring technique showed wide scope using various substituted indoles and isatins, promoting reactions within 15–75 minutes to yield trisindolines 3, 15, 17, 22, 24, 25, 28 and 29 in 80–98% yield (Scheme 12). On other side, the grinding method was conducted by mortar and pestle (0.1 mol%, 0.06 g catalyst/1 mmol of isatins), requiring considerably shorter reaction times (1–6.5 minutes) than the preceding method. However, yields were relatively lower ranging from 63 to 98% yields. In the grinding technique, the starting materials were pulverized to fine powder which generated local heat that sped the reaction. Interestingly, nano-SiO_2_ catalyst produced best yields of trisindolines (98% for both techniques) with 5-nitroisatin 28, while *N*-Bn isatins 25 and 24 relatively gave better yield under magnetic stirring (98% yields).^52^ As a notable feature of the protocol and while the attempted multi-step one pot preparation of unsymmetrical 3,3-di(indolyl)indolin-2-ones failed to produce the desired products, nano-SiO_2_ efficiently catalyzed the synthesis of various unsymmetrical trisindolines 174 and 177–179 using the two reported methods (Scheme 12).^52,53^

Another mechanical synthesis was achieved using ball-milling technique. The method was introduced as an eco-friendly solvent-free method for the synthesis of trisindoline in the presence of silica gel (SiO_2_) as acid catalyst and grinding medium.^54^ Trisindoline 3 was afforded after 7 h of grinding in 62% yield, compared to the grinding technique by mortar and pastel using nano-SiO_2_ as a catalyst.^52^

Azizian *et al.* (2006) demonstrated silica sulfuric acid SiO_2_–OSO_3_H-catalyzed condensation reaction (0.2 g SiO_2_–OSO_3_H/1 mmol isatins) of indoles with various isatins in DCM at room temperature for 2–3.5 h. Trisindolines 3, 5, 18, 22–25, 27, 33, 40 and 89 were obtained in 87–94% yields. Unsubstituted trisindoline 3 was obtained in best yield under shortest reaction time. Both EDG and EWG attached to C-5 isatin did not impact the yield appreciably but substrates with EWG required longer reaction time than those with EDG. Methyl on C-2 of the indole ring and EDG or EWG substituents on the NH of isatin did not affect the yield either. Further, the catalyst was recycled five times without loss in yield.^55^

Similarly, Chakrabarty *et al.* (2006) further demonstrated the application of H_3_PO_4_–SiO_2_ in the synthesis of trisindoline 3 (80% yield).^56^

Nikoofar *et al.* (2018) compared the solvent-free stirring and grinding techniques during the synthesis of symmetrical trisindolines 3, 5, 15, 17, 22, 24, 25 and 27–29 using HNO_3_@nano SiO_2_ catalyst (Table 3). Both methods gave 80–96% yields. However, the reaction using grinding method proceeded within 2–5 minutes while the stirring method required 20–40 minutes. Under both methods, 5-nitroisatin was the most sluggish substrate while trisindoline 3 was produced in >94% yield *albeit* it required longer reaction times. Unsymmetrical trisindolines 174 and 177–179 were also successfully prepared by reacting 1 eq. of isatin and 2 eq. of different indoles. Grinding technique afforded products 174 and 177–179 in 72–78% yields within 5–10 minutes, while stirring technique afforded the same in 71–80% within 5–60 minutes (Scheme 12).^53^ Compared to a previous method by the same authors using nano-SiO_2_ catalyst,^52^ this catalyst was comparable in terms of yield and reaction time for both techniques (Table 3).

**Table 3:** Reactions of isatins and indoles using nano-SiO_2_ and HNO_3_@nano-SiO_2_ under stirring and grinding techniques

Catalyst	Nano-SiO_2_ (0.1 mol%)^52^				HNO_3_@nano-SiO_2_ (ref. 53)			
	Symmetrical trisindoline		Unsymmetrical trisindoline		Symmetrical trisindoline		Unsymmetrical trisindoline	
	Stirring	Grinding	Stirring	Grinding	Stirring	Grinding	Stirring	Grinding
Time (min)	15–75	1–6.5	120–150	7–10	20–40	3–5	5–60	5–10
Isolated yields (%)	80–98	63–98	80–83	70–81	80–96	80–95	71–80	72–78

**Scheme 13:**
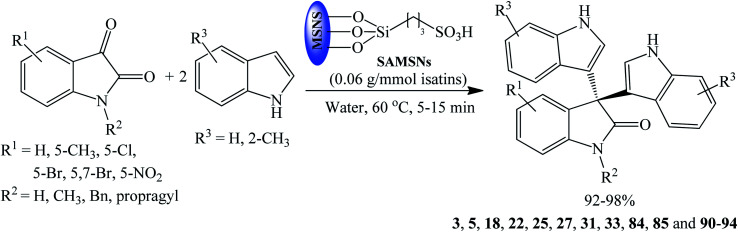
SAMSNs catalyzed synthesis of trisindolines 3, 5, 18, 22, 25, 27, 31, 33, 84, 85 and 90–94.

Sulfonic acid-functionalized mesoporous silica nanoparticles (SAMSNs) were used by Mehrasbi *et al.* (2014) as heterogeneous and recyclable catalyst to synthesize trisindolines 3, 5, 18, 22, 25, 27, 31, 33, 84, 85 and 90–94 in water at 60 °C over 5–25 minutes (Scheme 13). SAMSNs was prepared by incorporating a mercaptopropyl moiety into mesoporous silica nanoparticles, followed by oxidation of the SH group with H_2_O_2_ to SO_3_H. The catalyst showed wide scope for substituted isatins with outstanding yields of 90–98%. Trisindoline 3 gave the best yield within 10 minutes. Generally, isatins bearing EWG gave better yield within short reaction time than methyl-substituted isatin. The catalyst also worked well for bulky isatins, *N*-benzyl-5,7-dibromoisatin and 5,7-dibromoisatin.^57^

**Scheme 14:**
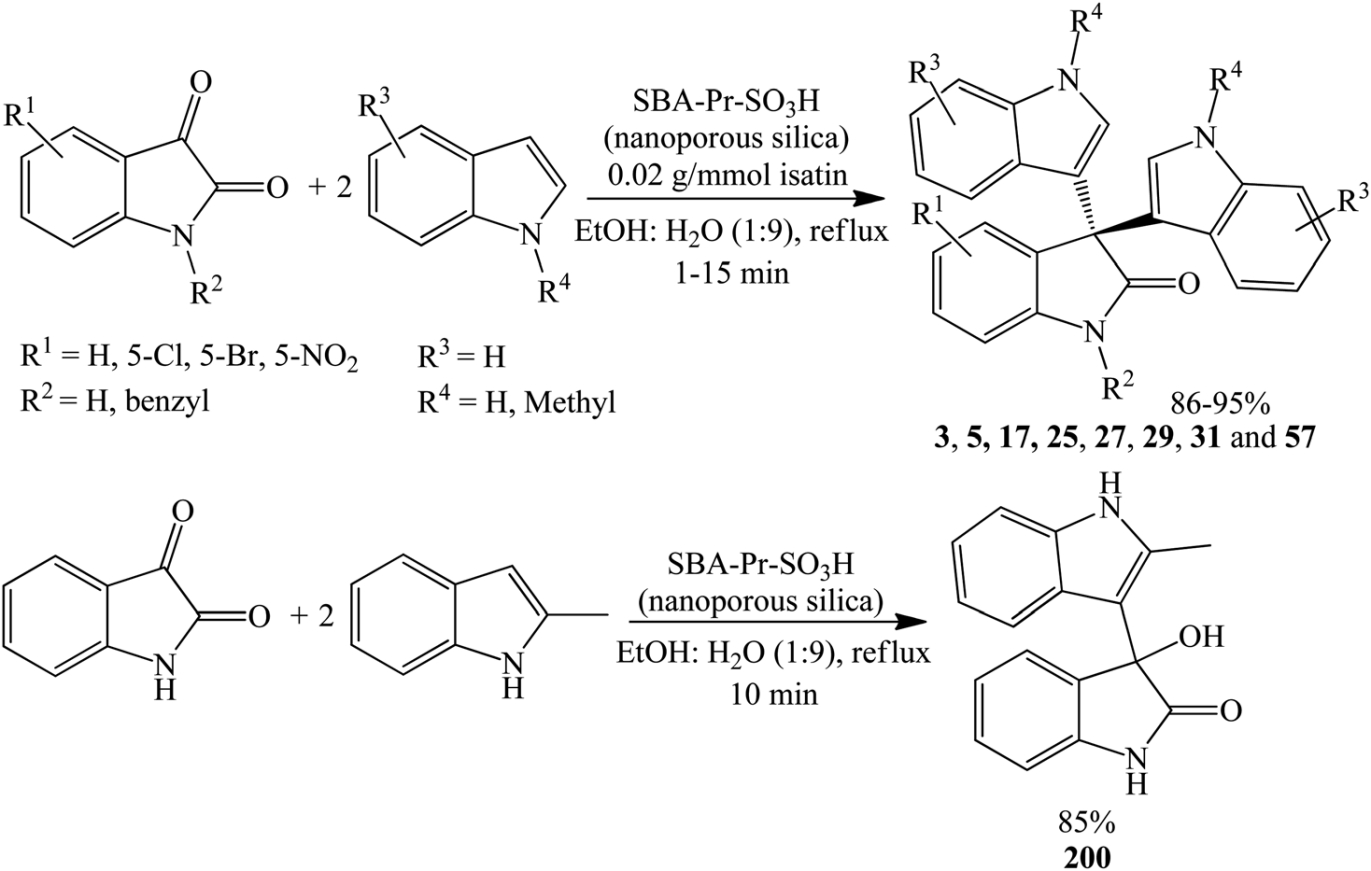
SBA-Pr-SO_3_H catalyzed synthesis of symmetrical 3,3-di(indolyl)oxindoles 3, 5, 17, 25, 27, 29, 31 and 57 and 3-hydroxy-3-(2-methyl-1*H*-indol-3-yl)indolin-2-one 200.

SBA-15 (SBA: Santa Barbara Amorphous) is mesoporous silica which possess uniform-sized nanopores with a large surface area and high thermal stability.^58^ SBA-Pr-SO_3_H is a nanoporous solid acid catalyst incorporating sulfonic acid-functionalized silica (SBA-15). Ziarani *et al.* (2015) synthesized 3, 5, 17, 25, 27, 29, 31 and 57 in 86–95% yields within 1–15 minutes by refluxing indoles and isatins in H_2_O : EtOH (9 : 1) in the presence of SBA-Pr-SO_3_H as reusable catalyst (Scheme 14). Substitution of NH of indoles with methyl groups reduces the reaction time and/or enhances the yields. EWG groups (halogen, nitro) on C-5 of isatin did not affect the reaction time or yield appreciably. *N*-allyl trisindoline was not formed even after 48 h while *N*-benzyl trisindoline 25 was obtained within 3 minutes in 90% yield. Unexpectedly, instead of forming the desired trisindoline 22, compound 200 was obtained in 95% yield when 2-methylindole was employed as a substrate.^59^

**Scheme 15:**
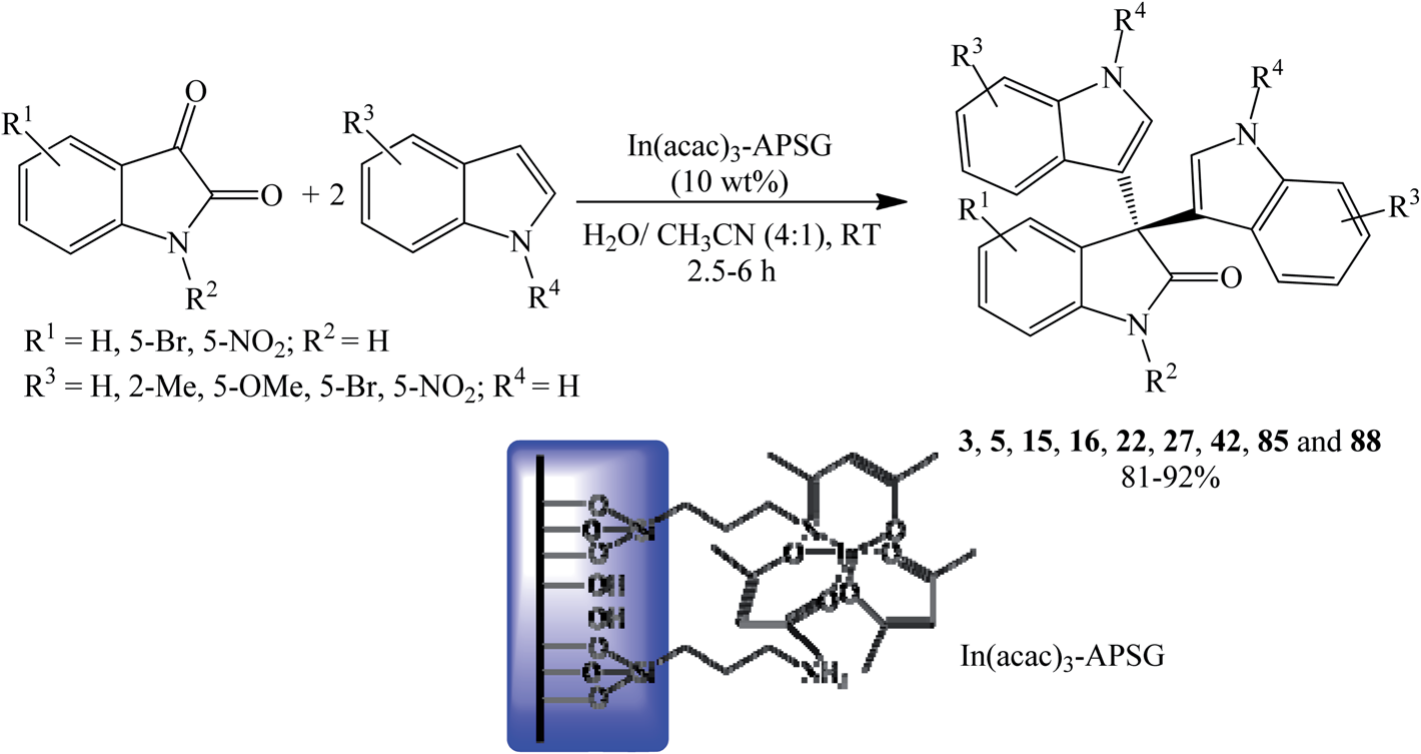
In(acac)_3_-APSG catalyzed synthesis of symmetrical 3,3-di(indolyl)oxindoles.

The modification of silica gel with 3-aminopropyltriethoxy silane results in aminopropylsilica gel (APSG). Subsequent anchoring of indium(iii) acetylacetonate complex to aminopropylsilica gel (APSG) gave In(acac)_3_-APSG which was investigated as a reusable heterogeneous catalyst for oxindole synthesis. Sharma and Sharma (2010) utilized this catalyst (10 wt%) to prepare trisindolines 3, 5, 15, 16, 22, 27, 42, 85 and 88 in 81–93% yield in water/acetonitrile (4 : 1) solvent system within 2.5–6 h (Scheme 15). Indoles with EDGs showed better reactivity than those with EWGs (5-nitroindole) which showed complete inertness when reacted with 5-nitro or 5-bromoisatin. Additionally, 5-bromoindole did not undergo reaction with 5-nitroisatin.^60^

##### 3.1.3.5 Nano-SiO_2_ supported boron trifluoride catalyzed reactions

Boron trifluoroide-etherate (BF_3_·OEt_2_) is a Lewis acid homogeneous catalyst which has been used in many organic transformations. Heterogenization of BF_3_ is possible if anchored on solid materials. One such example is the nano-SiO_2_–BF_3_^−^CH_3_OH_2_^+^ which has been synthesized by mixing BF_3_·OEt_2_ and preheated silica gel in MeOH and stirring for 3 h at room temperature. The catalyst was further applied for trisindoline synthesis by Saffar-Teluri (2013). Indoles and isatins were treated with a catalytic amount of nano-SiO_2_–BF_3_^−^CH_3_OH_2_^+^ (0.3 g mmol^−1^ isatins) in methanol under reflux conditions to afford trisindolines 3, 5, 18, 25, 27 and 33 in 93–97% yields within 0.17–0.5 h. The condensation reaction of this catalytic system depended on the electronic properties of the isatins substrate. For instance, 5-methylisatins reacted much faster than 5-nitroistatins. On the other hand, indoles with EDGs or EWGs reacted very smoothly with isatins, producing outstanding yields of 93–95%. *N*-methyltrisindoline 18 or *N*-benzyltrisindoline 25 were also successfully prepared in 96% and 97% yields, respectively.^61^

##### 3.1.3.6 PWA/MCM-41 catalyzed reactions

MCM-41 belongs to mesoporous silica and aluminosilicate materials featuring hexagonal pore arrangement.^62^ Phosphotungstic acid (PWA) supported on silica materials has been extensively utilized in organic transformations, especially in Friedel–Crafts reaction. Xing *et al.* (2018) reported efficient electrophilic coupling reaction between indoles and isatins using 60 wt% PWA/MCM-41 (0.0050 g/0.1 mmol isatins) in THF for 2.5 h at room temperature to give trisindolines 3, 18, 22, 30, 31, 33, 42, 44, 56, 69, 71 and 95–98 in 55–99% yields. When the ratio of PWA/MCM-41 surpassed 60 wt%, the chemical yields of the trisindolines declined, suggesting that the excess amounts of PWA blocked the MCM-pores, thus highlighting the role of the pores in facilitating the coupling reaction. Unsubstituted trisindoline 3 was formed in excellent 99% yield. Having a methyl group on various positions of the indoles (C-2 or C-5 or C-6) as well as a nitro group on C-5 or C-6 gave moderately lower yields (55–80%) than indoles bearing fluorine, and chlorine substituents on C-5 or C-6 positions (96–98%). On the contrary, 1-methylisatin and 5-methylisatin, as well as 5-chloro, 7-fluoro, or 4-bromo-isatins reacted smoothly with indoles, furnishing trisindolines in excellent 60–99% yields. Interestingly, unlike its 5- and 7-halogenated analogues, 4-bromoisatin showed the least reactivity affording the product 95 in only 60% yield. The catalyst exhibited high activity and was recycled and reused over six cycles with an overall drop of 9% in yield.^63^

##### 3.1.3.7 Graphene oxide catalyzed reactions

Graphene oxide catalyzed the reaction between indoles and isatins in water media under ambient temperature to give trisindolines 3, 5, 15, 18, 21–25, 27, 31, 84–88, 99 and 100 in 65–98% yields within 1.5–5 h.^64^ The best yields were obtained when the indoles were substituted with EDG on C-2 and/or isatins were substituted with EWG on C-5. EWG on C-5 of the indole decreased the yield and/or prolonged the reaction time. *N*-Methylisatin exhibited better reactivity than *N*-benzylisatin.

##### 3.1.3.8 [Amb]l-prolinate catalyzed reactions

**Scheme 16:**
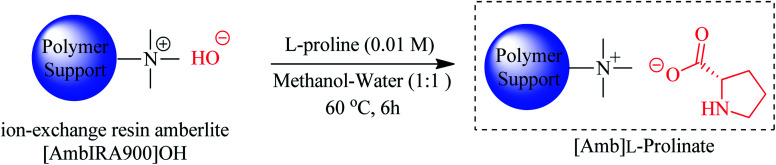
Synthesis of [Amb]l-prolinate ion-pair hybrid.

Keshavarz *et al.* (2015) immobilized l-proline on the surface of the cationic anion-exchange resin amberlite IRA900OH to obtain [Amb]l-prolinate. This was achieved by treating a 1 : 1 methanol/water solution of 0.01 M l-proline with amberlite IRA900OH at 60 °C for 6 h (Scheme 16).^65^

When isatins (1 eq.) and indoles (2 eq.) were refluxed in ethanol and treated with 10 mol% [Amb]l-prolinate catalyst, trisindolines 3, 5, 15, 17, 18, 21–23, 27, 31, 32, 37, 57, 61, 68, 84, 86, 99 and 101–104 were obtained in 79–99% yields within 8–15 minutes. Both EDG and EWG substituents attached to isatin were well-tolerated. Furthermore, 2-methylindoles were more reactive than indole itself. Interestingly, the heterogeneous catalyst could be reused up to 8 times *albeit* with a slight 5% drop in the yield.^65^ In addition, the catalyst is characterized by very low leaching of l-proline during 8 cycles. Another notable feature of this catalyst is its superiority compared to other catalysts in terms of yield and reaction times.

#### 3.1.4 Halogens-based catalysts

The use of halogens-based catalysts to prepare 3,3-diindolyl-2-oxindoles is outlined in the next subsections.

##### 3.1.4.1 Iodine catalyzed reactions

**Scheme 17:**
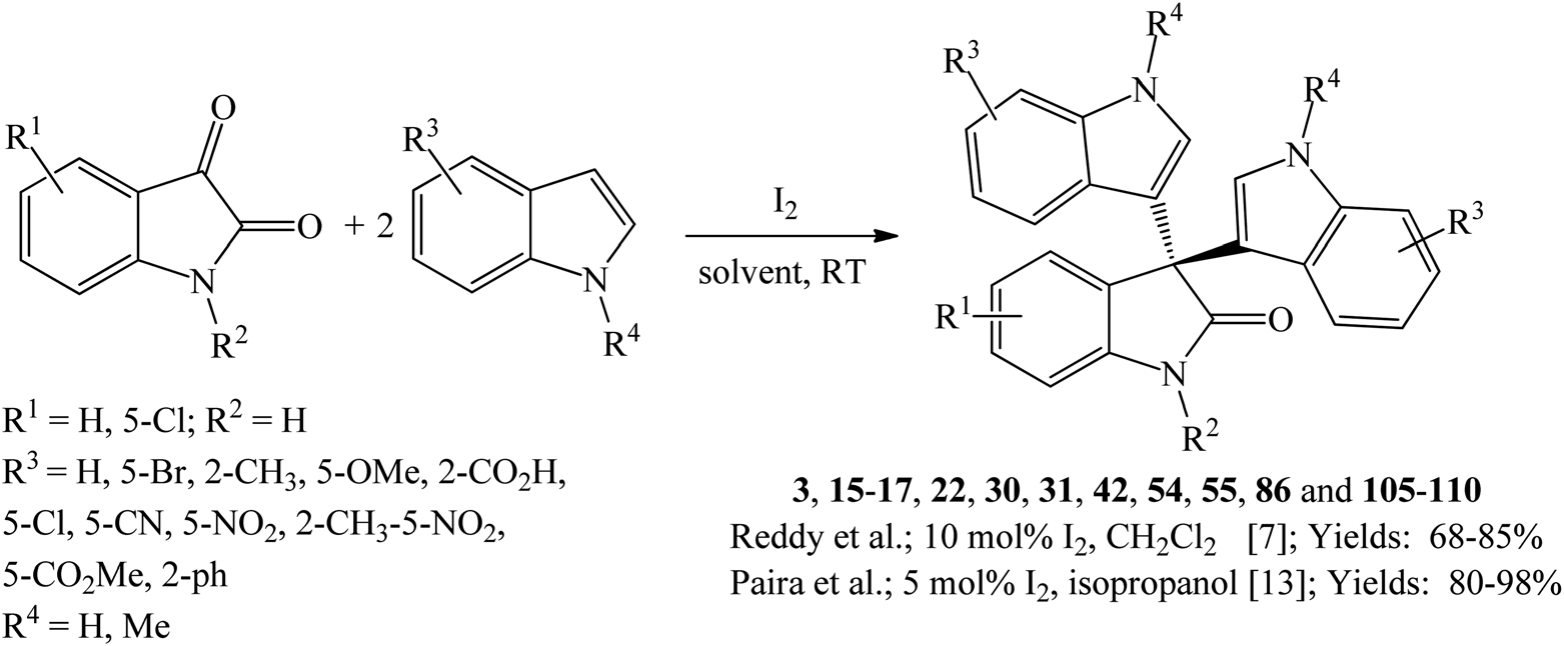
Iodine-catalyzed synthesis of trisindolines 3, 15–17, 22, 30, 31, 42, 54, 55, 86 and 105–110.

Paira *et al.* (2009) reported that the reaction of isatins with indoles using 5 mol% I_2_ in isopropanol at room temperature gave 80–95% yields of trisindolines 3, 15–17, 22 and 105 within 15–45 minutes (Scheme 17).^13^ Even electron-deficient indoles reacted very well under these conditions. However, 5-methoxyindole needed 3 h to give 80% yield of trisindoline 19. Later, Reddy *et al.* (2012) disclosed that 10 mol% of I_2_ catalyzed the same reaction in dichloromethane but required longer reaction times (12–22 h) and generally gave lower yields of trisindolines 3, 15, 17, 22, 30, 31, 42, 54, 55, 86 and 106–110 (68–85% yields) especially when 5-chloroisatin was used (Scheme 17). The I_2_-catalyzed reaction showed a wide scope with electron-poor and electron-rich indoles.^7^ In comparison, the method by Paira *et al.* has advantages over Reddy *et al.*'s method in terms of higher yields and shorter reaction times (Scheme 17).

##### 3.1.4.2 Bromine catalyzed reactions

**Scheme 18:**
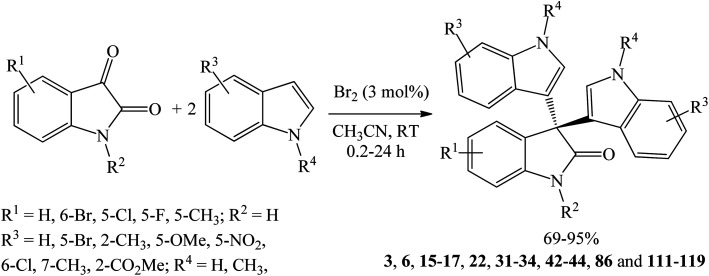
Br_2_-catalyzed synthesis of trisindolines 3, 6, 15–17, 22, 31–34, 42–44, 86 and 111–119.

Huang *et al.* (2015) investigated several bromine-based catalysts including Br_2_, *N*-bromosuccinimide (NBS), and 40% HBr in water. Br_2_ (3 mol%) was the most active catalyst where trisindolines 3, 6, 15–17, 22, 31–34, 42–44, 86 and 111–119 were obtained in 69–95% yields within 0.2–24 h in acetonitrile at room temperature (Scheme 18).^36^ Both EDGs and EWGs at any position on indoles gave trisindoline in excellent yields (>90%), except for 2-CO_2_Et indole 119 and 5-nitroindole 112 which gave lower yields of 69% and 77%, respectively. *N*-Tosylindoles and *N*-pivalylindoles were unreactive under these conditions.

#### 3.1.5 Ionic liquids and related catalysts

Ionic liquids have been used extensively as environmentally benign and green reaction solvents and catalysts in many organic transformations due to their special physicochemical characteristics.^66^ Several ionic liquids successfully catalyzed the reaction between indoles and isatins to afford trisindolines.

##### 3.1.5.1 *N*,*N*,*N*,*N*-Tetramethylguanidinium trifluoroacetate catalyzed reactions

**Scheme 19:**
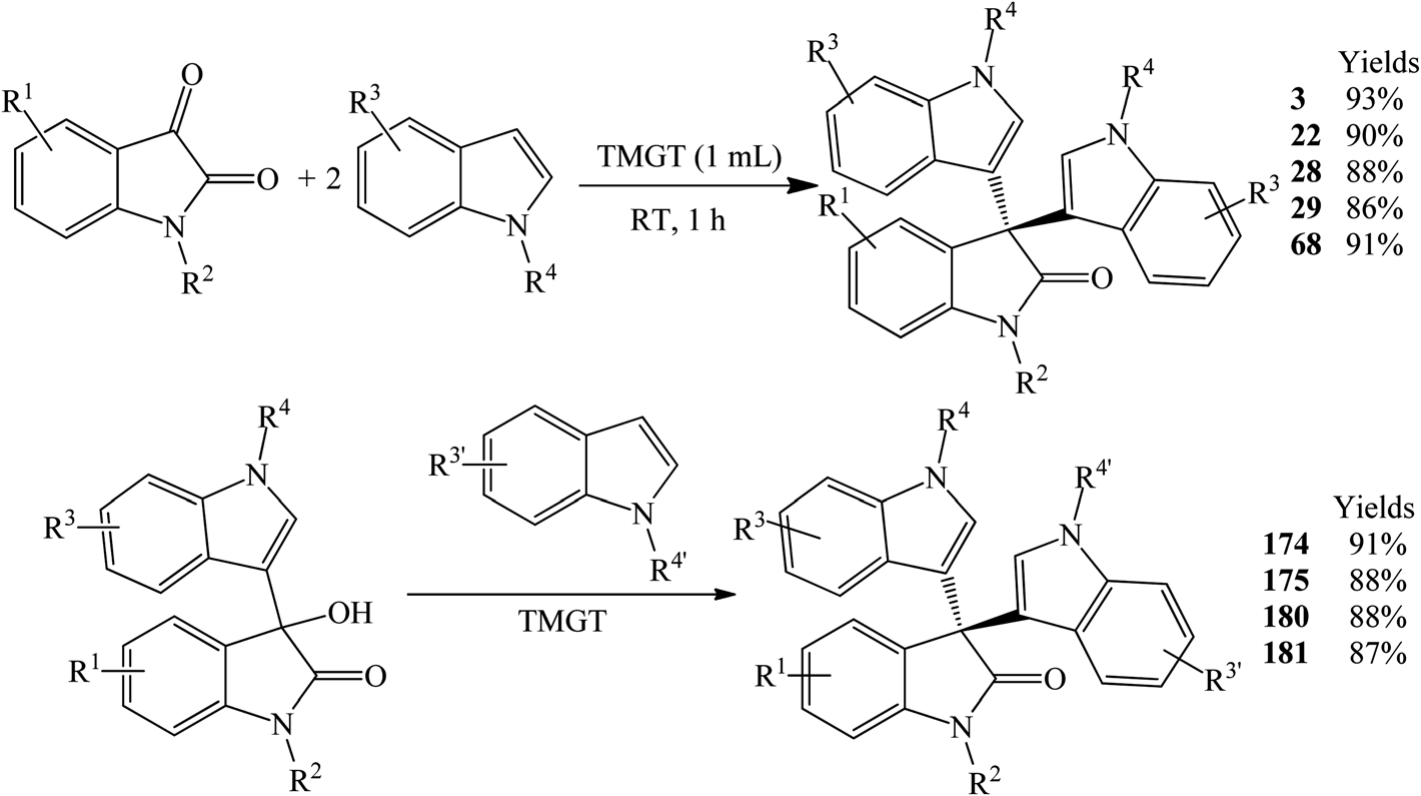
Preparation of trisindolines catalyzed by TMGT.

The reaction using *N*,*N*,*N*,*N*-tetramethylguanidinium trifluoroacetate (TMGT) as a solvent and a catalyst gave symmetrical trisindolines 3, 22, 28, 29 and 68 in 86–93% yield at room temperature within a short reaction time of 1 h (Scheme 19).^27^ Both isatins with EWDs and EDGs reacted smoothly. While *N*-methylindoles reacted smoothly, the scope of indoles with EWGs has not been evaluated under these conditions. Asymmetrical trisindolines 174, 175, 180 and 181 (87–91% yields) were also prepared under the same conditions by reacting an equimolar amount of 3-hydroxy-3-indolyl-2-indolones and other indoles (Scheme 19).

##### 3.1.5.2 1-Butyl-3-methyl-imidazolium hydrogen sulphate catalyzed reactions

**Scheme 20:**
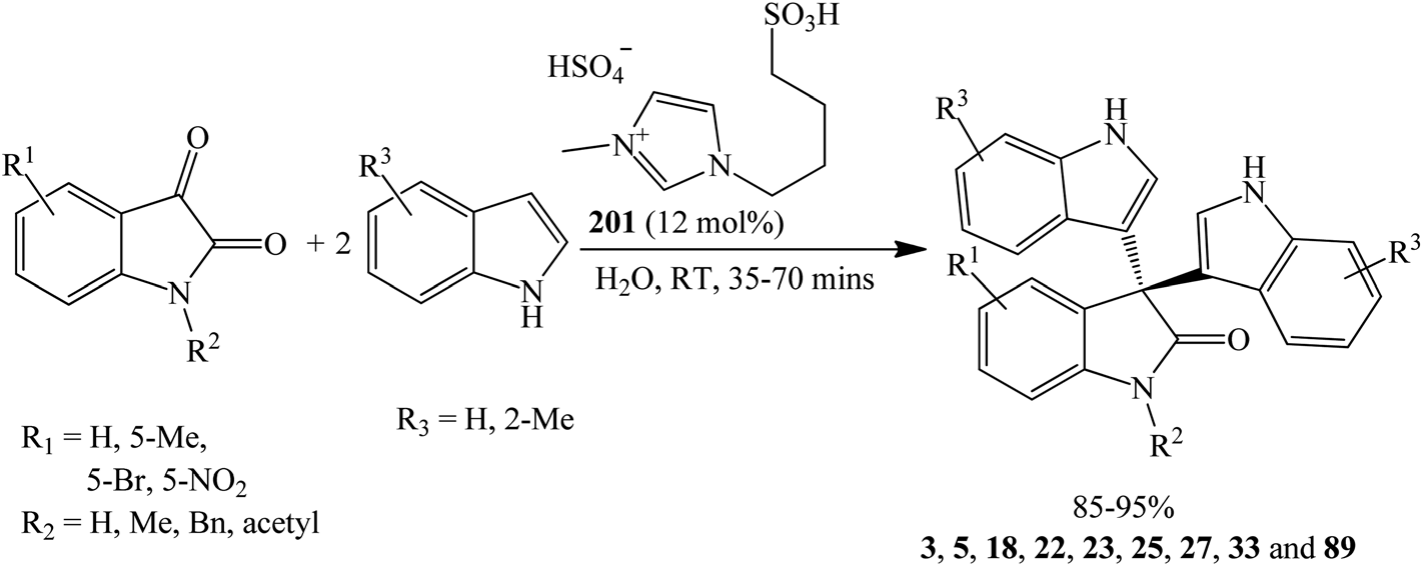
Synthesis of trisindolines 3, 5, 18, 22, 23, 25, 27, 33 and 89 catalyzed by [(CH_2_)_4_SO_3_HMIM][HSO_4_] 201.

Likewise, the Brønsted acid ionic liquid 1-butyl-3-methyl-imidazolium hydrogen sulphate ([(CH_2_)_4_SO_3_HMIM][HSO_4_]) 201 (12 mol%) gave the trisindolines 3, 5, 18, 22, 23, 25, 27, 33 and 89 in 85–95% yields in water at room temperature within a shorter reaction time of 35–55 minutes (Scheme 20).^67^ Here again, isatins with either EDGs or EWGs afforded excellent yields. Interestingly, *N*-methylisatin, *N*-benzylisatin, and *N*-acetylisatin also gave the trisindolines 18, 25 and 89 in >90% yields. The catalyst was recycled and reused five times without significant loss in the yield of the trisindolines.

##### 3.1.5.3 1,4-Diazabicyclo[2.2.2]octane hydrogen sulfate catalyzed reactions

Tong *et al.* (2016) evaluated ionic liquid [DABCO-H][HSO_4_] (1,4-diazabicyclo[2.2.2]octane hydrogen sulfate) as catalyst for indolylation of isatin. The reaction furnished 95% yield of trisindoline 3 within 2 h. Substituted indoles or isatins has not been evaluated. Like other ionic liquid catalyst, [DABCO-H][HSO_4_] was also reused up to six times without significant loss in the catalytic activity.^68^

##### 3.1.5.4 1,4-Diazobicyclo[2.2.2]octanes catalyzed reactions

**Scheme 21:**
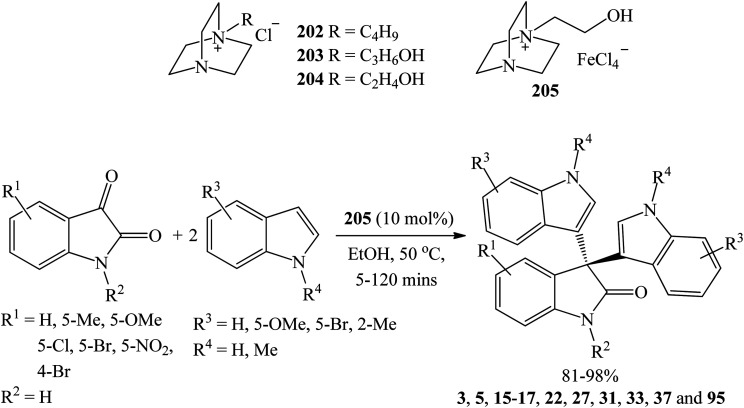
[Dabco-C_2_H_4_OH][FeCl_4_]-catalyzed synthesis of 3, 5, 15–17, 22, 27, 31, 33, 37 and 95.

Gu *et al.* (2018) investigated several 1,4-diazobicyclo[2.2.2]octanes (Dabco) as ionic liquid-based catalysts including [Dabco-C_4_H_9_]Cl 202, [Dabco-C_3_H_6_OH]Cl 203, [Dabco-C_2_H_4_OH]Cl 204, and [Dabco-C_2_H_4_OH][FeCl_4_] 205. After extensive screening, 10 mol% [Dabco-C_2_H_4_OH][FeCl_4_] 205 emerged as the best catalyst giving the trisindolines 3, 5, 15–17, 22, 27, 31, 33, 37 and 95 in 85–97% yields in ethanol at 50 °C within 1 h (Scheme 21).^69^ The reaction showed a wide substrate scope where substituted indoles (halogen, methoxy) and isatins (halogen, nitro, methyl) gave the desired trisindolines in excellent yields. However, the reaction between 4-bromoisatin and indole required longer reaction time of 2 h to afford 81% yield of 95. 5-Nitroisatin and 5-methylisatin reacted smoothly with indole and gave better yield (96% of 27 and 98% of 33, respectively) than in the case when [(CH_2_)_4_SO_3_HMIM][HSO_4_] was used.^67^ The catalyst 205 was recycled and reused six times with a 10% overall decrease in the yield of 3.^69^

##### 3.1.5.5 Prolinium triflate catalyzed reactions

Since several ionic liquids are air and moisture sensitive, their catalytic activity tends to decrease over time. Protic prolinium triflate (PrOTf), obtained by treating aqueous l-proline with triflic acid, was proposed as water-tolerant ionic liquid. Shiri *et al.* (2013) demonstrated the usefulness of 10 mol% prolinium triflate as a homogeneous catalyst in acetonitrile for the synthesis of trisindolines 3, 18 and 22 in 90%, 89% and 94% yields, respectively.^70^ The reaction proceeded at room temperature but took 5 h for completion.

##### 3.1.5.6 Low transition temperature mixtures (LTTMs) catalyzed reactions

**Scheme 22:**
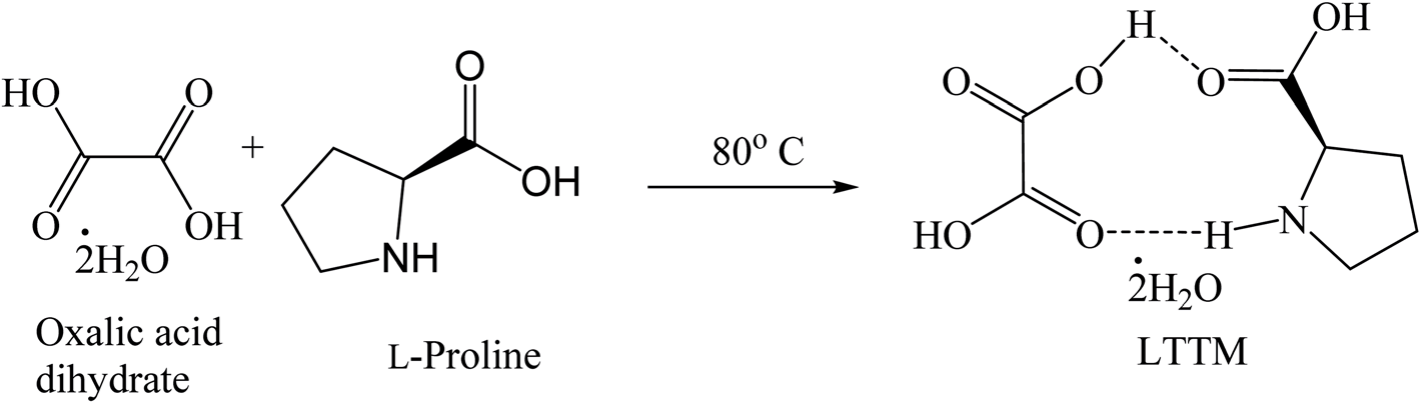
Preparation of low transition temperature mixture (LTTM).

**Scheme 23:**
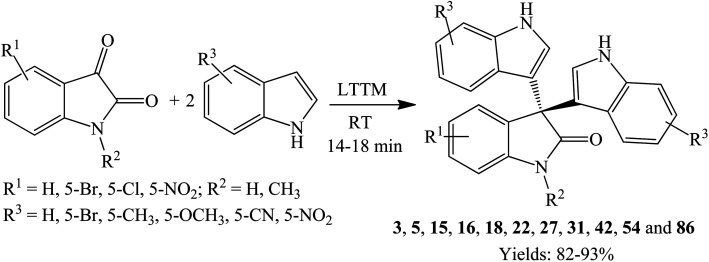
Reaction of isatins and indoles using LTTM catalyst.

Synthesis of trisindolines is also possible using low transition temperature mixtures (LTTMs), also called deep eutectic solvents (DES), comprising oxalic acid dihydrate and l-proline as the solvent/catalyst (Scheme 22). The reaction tolerates wide substrate scope and produces trisindolines 3, 5, 15, 16, 18, 22, 27, 31, 42, 54 and 86 in 80–93% yields within 14–18 minutes, even in the presence of EWGs such as CN and NO_2_ (Scheme 23).^71^ This is not surprising as the reactions between isatins and 5-cyanoindole or 5-nitroindole are often difficult and fail to proceed satisfactorily even with catalysts due to poor nucleophilicity.^39,60,72^ LTTM could be recycled and reused five cycles without significant loss of reactivity.^71^

#### 3.1.6 Metallic species-based catalysts

Several metal-based catalysts were used to catalyze the reaction between isatins and indoles to trisindolines. The following sub-sections highlight some of the important reagents used.

##### 3.1.6.1 Potassium aluminum sulfate (KAlSO_4_) catalyzed reactions

**Scheme 24:**
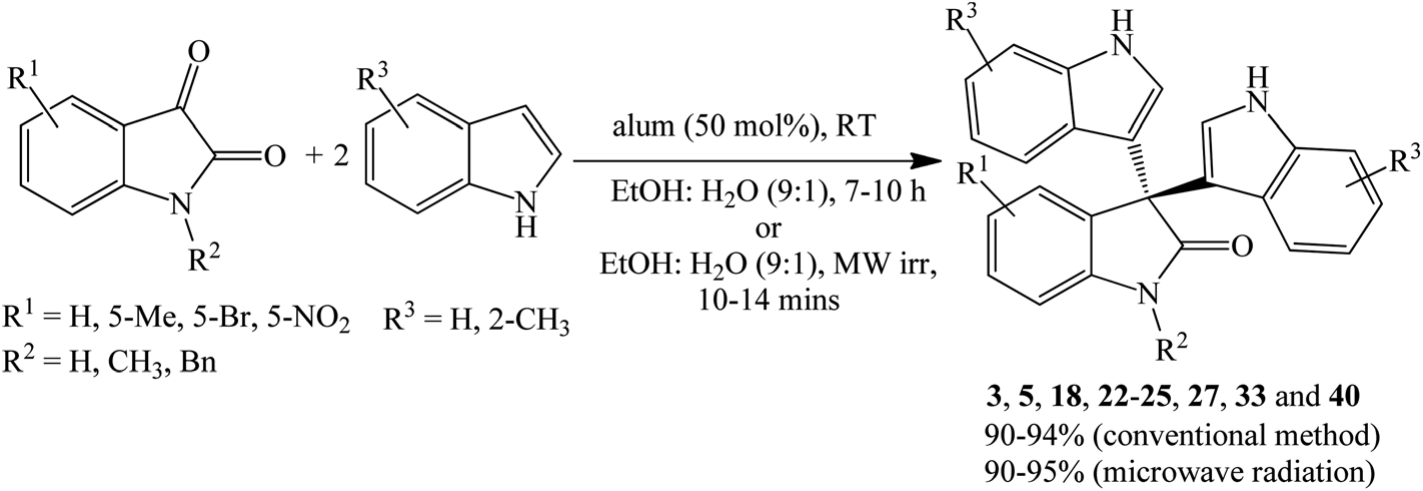
KAlSO_4_-catalyzed synthesis of 3,3-di(indolyl)oxindoles 3, 5, 18, 22–25, 27, 33 and 40.

Potassium aluminum sulfate (KAlSO_4_), also known as alum, successfully afforded trisindolines 3, 5, 18, 22–25, 27, 33 and 40 in excellent yields under conventional and microwave-assisted conditions (Scheme 24).^73^ The yields of both methods were comparable at >90%. While the microwave reaction in 90% ethanol at 150 watt irradiation was completed within 15 minutes, the conventional reaction in ethanol/water (2 : 3, v/v) at room temperature needed 7 h. However, there were no significant differences in the yields of the products obtained from both methods. The catalyst showed a wide substrate scope where *N*-substituted isatins, and isatins with EWGs and EDGs on C-5 gave excellent yields. Likewise, 2-methylindoles gave excellent yields.

##### 3.1.6.2 Ceric ammonium nitrate (NH_4_)_2_Ce(NO_3_)_6_ (CAN) catalyzed reactions

**Scheme 25:**
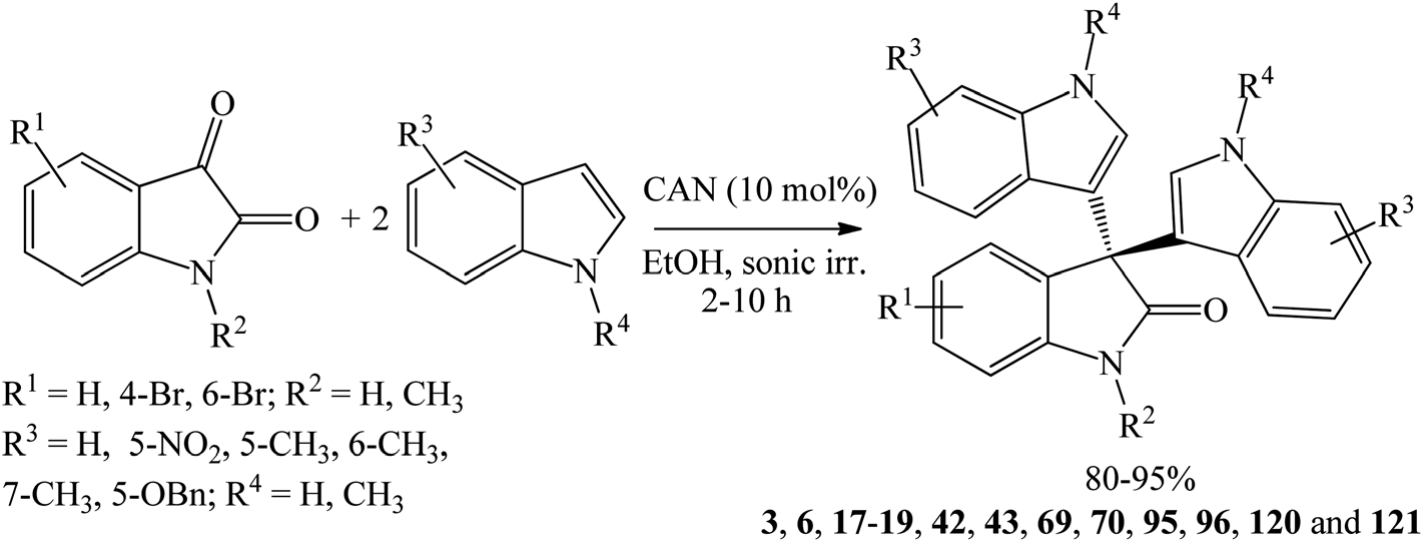
CAN-catalyzed synthesis of symmetrical trisindolines 3, 6, 17–19, 42, 43, 69, 70, 95, 96, 120 and 121.

Ceric ammonium nitrate (NH_4_)_2_Ce(NO_3_)_6_ (CAN) is well-known catalyst in organic transformation for its mildness, ease of handling and efficiency. 10 mol% CAN catalyzed the condensation of isatins with indoles under ultrasonic irradiation at room temperature and afforded symmetrical trisindolines 3, 6, 17–19, 42, 43, 69, 70, 95, 96, 120 and 121 in 80–95% yields within 2–10 h (Scheme 25).^74^ Reactions of substituted isatins with unsubstituted indoles took 2–3 h while reactions of unsubstituted isatins with substituted indoles took longer time of 7–8 h. The challenging 5-nitroindole reacted smoothly with isatin to afforded trisindoline 42 in 90% yield, but the 7-nitroindole isomer was unreactive. Nevertheless, indoles bearing methyl substituent at any position and isatins bearing bromo substituent at any position were all reactive and gave excellent yields.

**Scheme 26:**
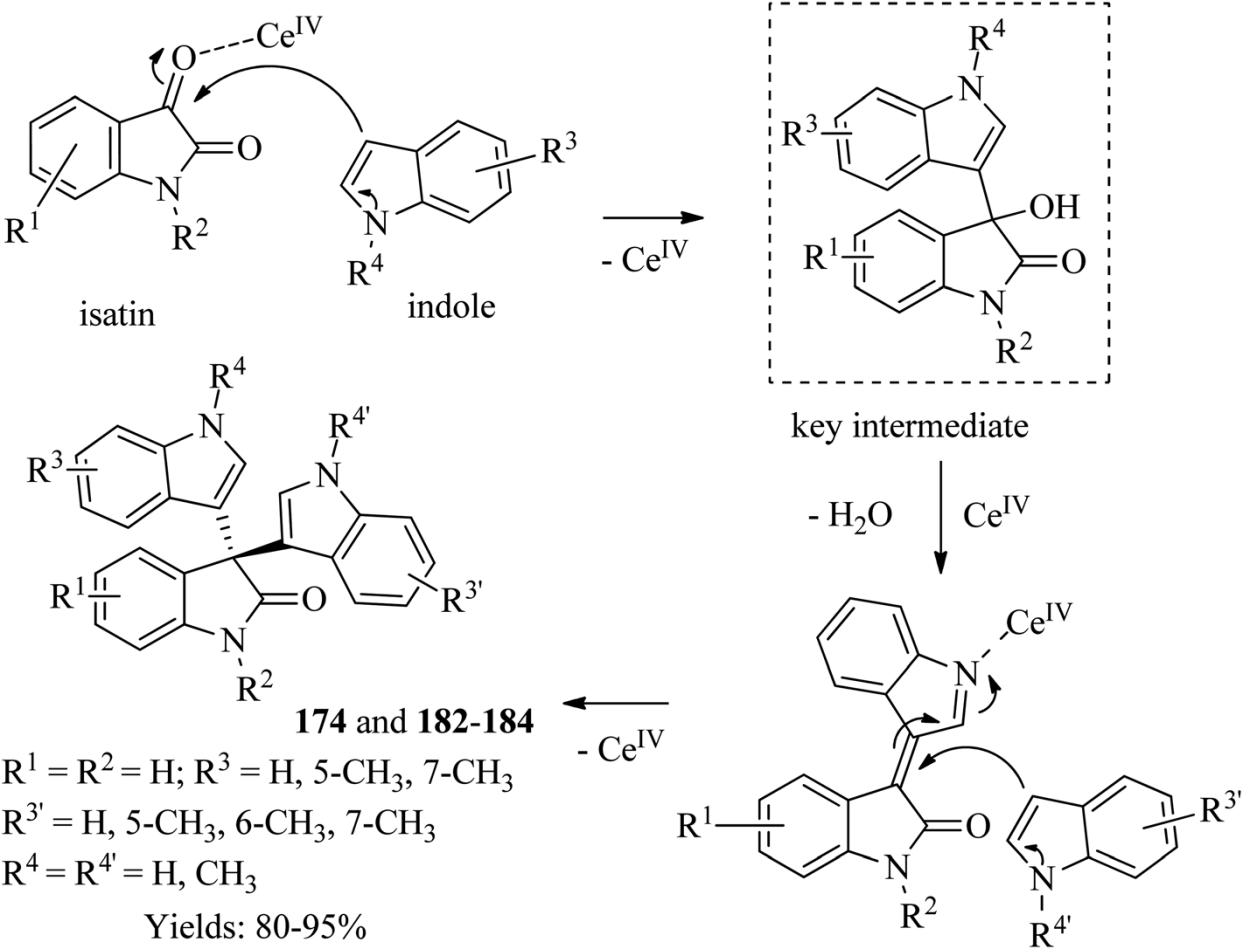
Mechanism of CAN-catalyzed synthesis of trisindolines.

The reaction mechanism involves activating the both the carbonyl group of isatin and the indole moiety by the Ce^IV^ atom of CAN (Scheme 26). Thus, in the first step, nucleophilic indole attacks the electrophilic carbonyl group of isatin to generate 3-hydroxy-3-(1*H*-indol-3-yl)indolin-2-one. The tertiary alcohol key intermediate undergoes dehydration to form the α,β-unsaturated imine-Ce^IV^ complex which upon attach by another indole at the β position gives the product and regenerate Ce^IV^. Evidence for the intermediacy of the 3-hydroxy-3-(1*H*-indol-3-yl)indolin-2-one species had been provided by independently preparing several derivatives of the alcohol key intermediate and converting it to the 3-hydroxy-3-(1*H*-indol-3-yl)indolin-2-one products by the action of CAN. The alcohol intermediate was exploited to prepare unsymmetrical trisindolines. Thus, by reacting an equimolar of 3-hydroxy-3-indolyl-2-indolones and indoles, unsymmetrical trisindolines 174 and 182–184 formed in 60–86% yields in 1–5 h (Scheme 26).

##### 3.1.6.3 Metal triflates catalyzed reactions

**Scheme 27:**
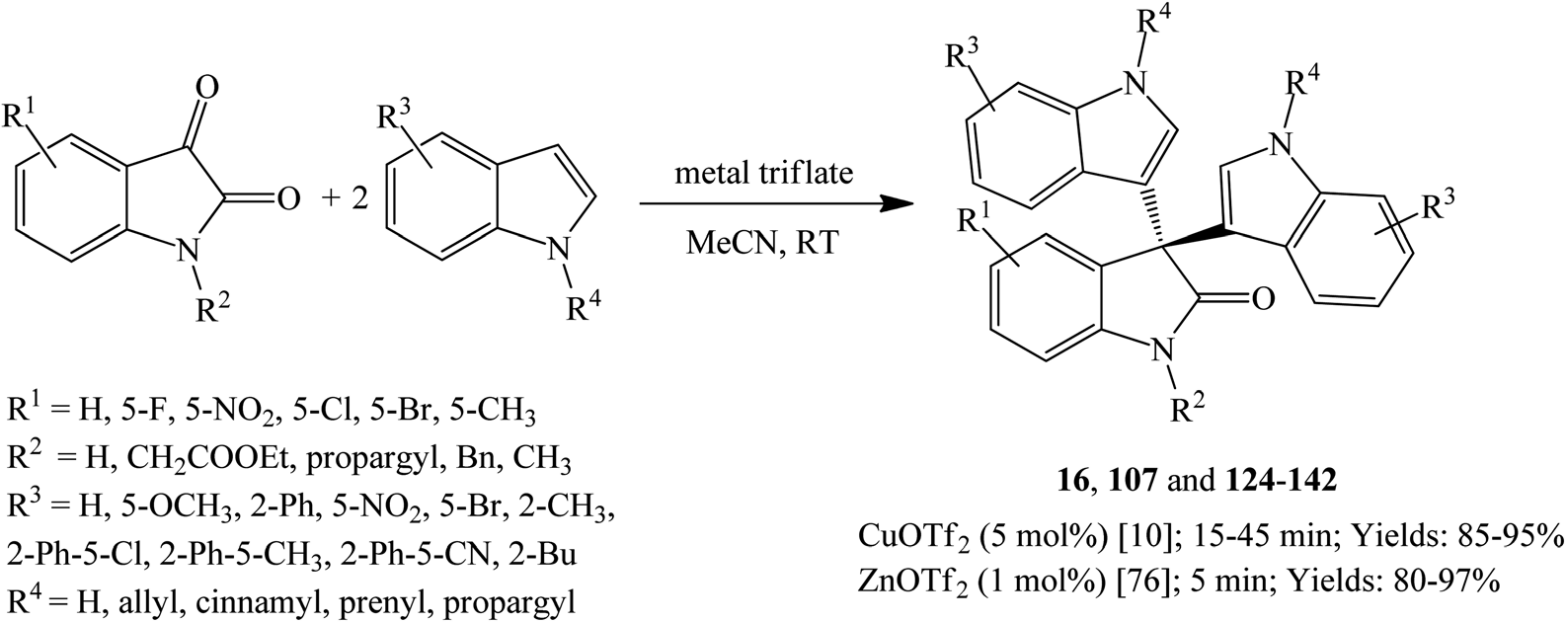
Metal triflates-catalyzed synthesis of trisindolines 16, 107 and 124–142.

Metal triflates such as CuOTf_2_, ZnOTf_2_ and BiOTf_2_ successfully catalyze the reaction between indoles and isatins to give trisindolines. For example, 2 mol% BiOTf_2_ catalyzed the reaction between indoles and isatins at room temperature within 2.5–4 h to give 82–95% yields of trisindolines 3, 15, 16, 22, 119, 122 and 123. Both EWGs and EDGs attached at any position of the indole gave considerably good yields.^75^ Praveen *et al.* examined CuOTf_2_ (5 mol%)^10^ and ZnOTf_2_ (1 mol%)^76^ and found them to be suitable catalysts especially when the NH of the indoles and isatins was substituted with various groups (Scheme 27). Both catalysts worked well to give similar high yields but the reaction with ZnOTf_2_ proceeded at a much faster rate within 5 minutes. Moreover, the use of ZnOTf_2_ did not cause isomerization of the (*E*)-cinnamyl substituents.^10,76^ Metal triflates seem to be ideal catalysts when NH substituted trisindolines are required.

##### 3.1.6.4 Zirconium catalyzed reactions

**Fig. 4:**
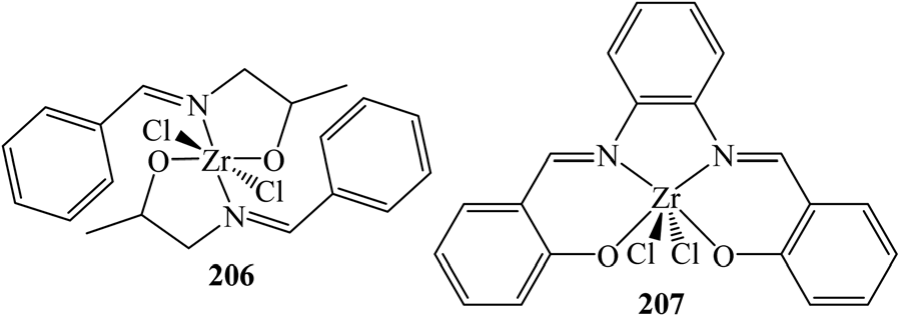
Zirconium(iv) Schiff base catalysts.

Several zirconium-based catalysts were also examined and found effective for the synthesis of trisindolines. 2 mol% zirconium(iv) Schiff base complex 206 (Fig. 4) gave trisindolines 3 and 22 in 97% yields at 25–27 °C in ethanol within 10–20 minutes.^77^

Zr(salphen)Cl_2_207 (1 mol%) afforded trisindoline 22 in 90% yield in less than a minute. The unsubstituted trisindoline 3 was obtained in 85% yield within 25 minutes.^78^ Likewise, the simple zirconium(iv) chloride (ZrCl_4_) (5 mol%) promoted the reaction to give trisindolines 3, 5, 15, 17, 18, 21, 22, 24, 25, 27 and 143 in 80–98% yields at 50 °C within 9–120 minutes.^79^ The order of the reactivity of *N*-alkylindole were *N*-methylindole > *N*-benzylindole > *N*-ethylindole. 5-Bromoindole also gave high yield. By adopting the same method, ZrCl_4_ catalyst was also evaluated for the synthesis of unsymmetrical trisindoline by reacting 3-hydroxy-3-indolyl-2-indolones (1 eq.) with various indoles (1 eq.) to obtain the desired trisindolines 174, 175, 177–179 and 185–187 with excellent 93–97% yields within 10–30 minutes.^79^

##### 3.1.6.5 Gold chloride (AuCl) catalyzed reactions

**Scheme 28:**
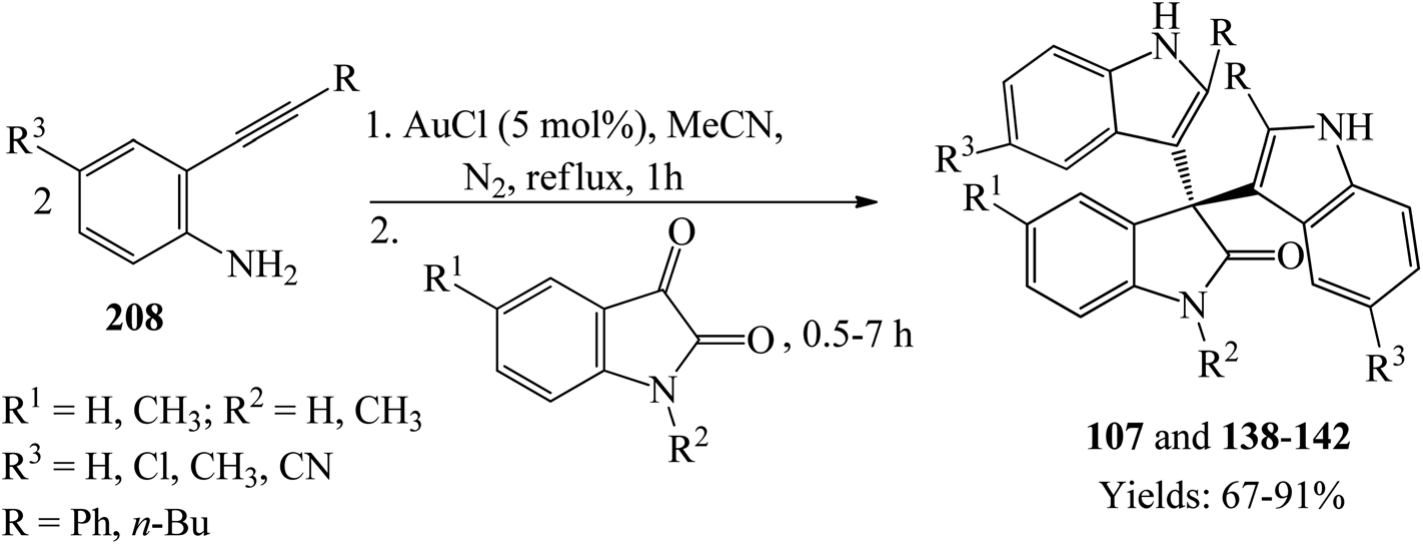
AuCl-catalyzed synthesis of trisindolines 107 and 138–142 by cycloisomerization/bis-addition of *o*-ethynylanilines to isatins.

Synthesis of trisindolines 107 and 138–142 proceeded in one-pot reaction by refluxing two equivalents of *o*-ethynylaniline 208 and one equivalent of the corresponding isatins in the presence of 5 mol% AuCl within 0.5–7 h to give 67–91% yield (Scheme 28).^80,81^*o*-Ethynylanilines 208 having phenyl substituents showed shorter reaction time and higher yields in comparison with *n*-butyl-*o*-ethynylaniline. EDG (*e.g.* methyl) on C-4 of *o*-ethynylaniline 208 exhibited better reactivity than EWG (*e.g.* cyano).

**Scheme 29:**
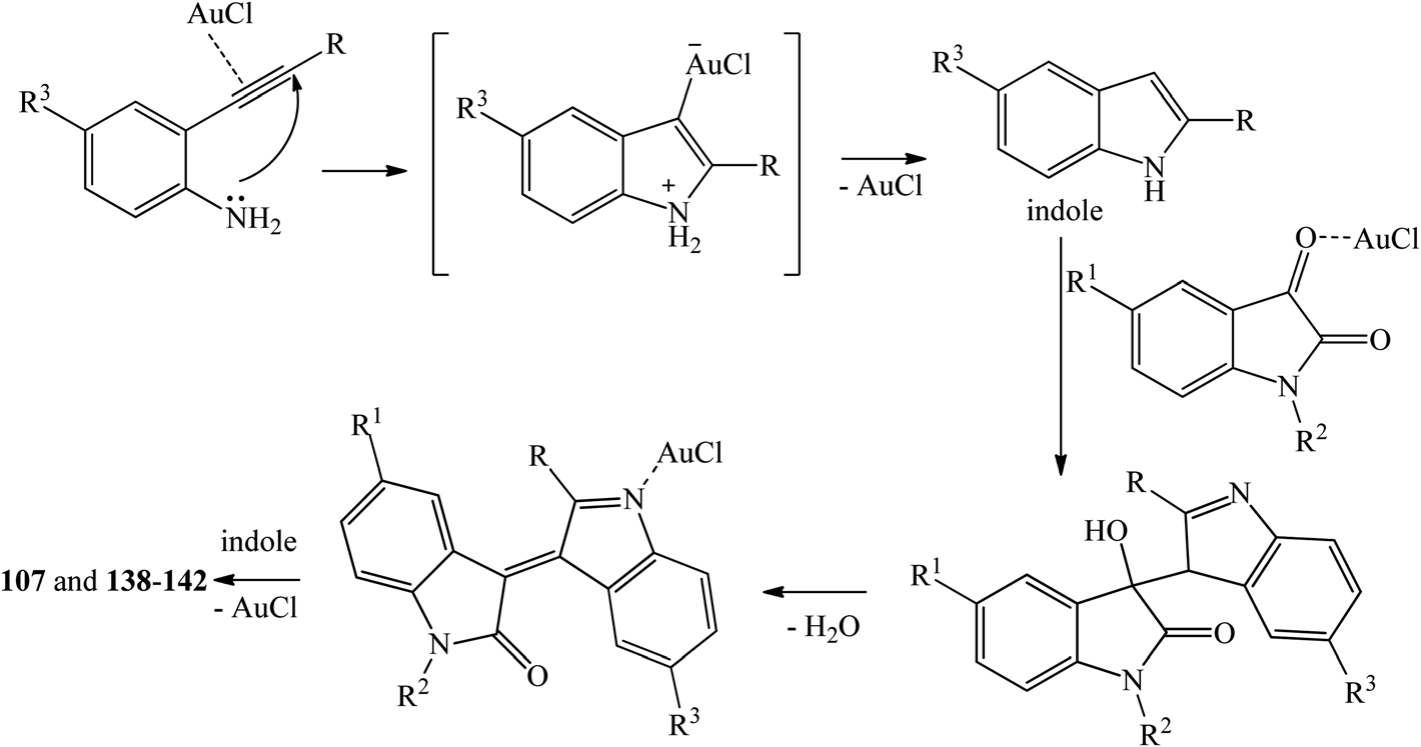
Plausible reaction mechanism of trisindoline synthesis by cycloisomerization/bis-addition of *o*-ethynylanilines.

Praveen *et al.* (2009) proposed a mechanism of this one-pot reaction as shown in Scheme 29.^80,81^ π-coordination of the alkyne residue with Lewis acidic AuCl forms a π-complex intermediate which undergoes intramolecular cyclization and subsequent proto-deauration to generate an indole and give back the AuCl (Scheme 29). The eliminated Lewis acid activates the C3 carbonyl oxygen of isatin, followed by an electrophilic addition reaction at C3 of the indole. The resulting tertiary alcohol undergoes dehydration and coordination with AuCl to form α,β-unsaturated azafulvene derivative, which reacts with another molecule of indole to form the products.

##### 3.1.6.6 Other metallic species catalyzed reactions

RuCl_3_·*n*H_2_O (5 mol%) gave 78–98% yields of trisindolines 3, 15, 17, 22, 54 in just 5–30 minutes in methanol at 50 °C. The electron deficient 5-cyanoindole gave 78% yield of 54.^82^

Iron salts are less toxic, cheap and environmentally friendly Lewis acid catalysts. Iron(iii) chloride (FeCl_3_) is a robust catalyst for many organic transformations. However, FeCl_3_ is sensitive to air and moisture thus requiring special care. Several trisindolines 3, 15–17, 30, 32, 42, 60, 61, 101, 124 and 144–148 were obtained in 78–95% yields within 10–60 minutes by employing 5 mol% anhydrous FeCl_3_. Both electron-rich and electron-poor indoles reacted smoothly. However, 5-fluoroisatin was less reactive than isatin.^8^

LiClO_4_ (20 mol%) afforded 88–93% yields of trisindolines 3, 17, 22, 28, 31 and 57 when the reaction was performed in ethanol at 60 °C.^26^

#### 3.1.7 Nanoparticle metal based catalysts

##### 3.1.7.1 Palladium nanoparticle (PdNPs) catalyzed reactions

**Scheme 30:**
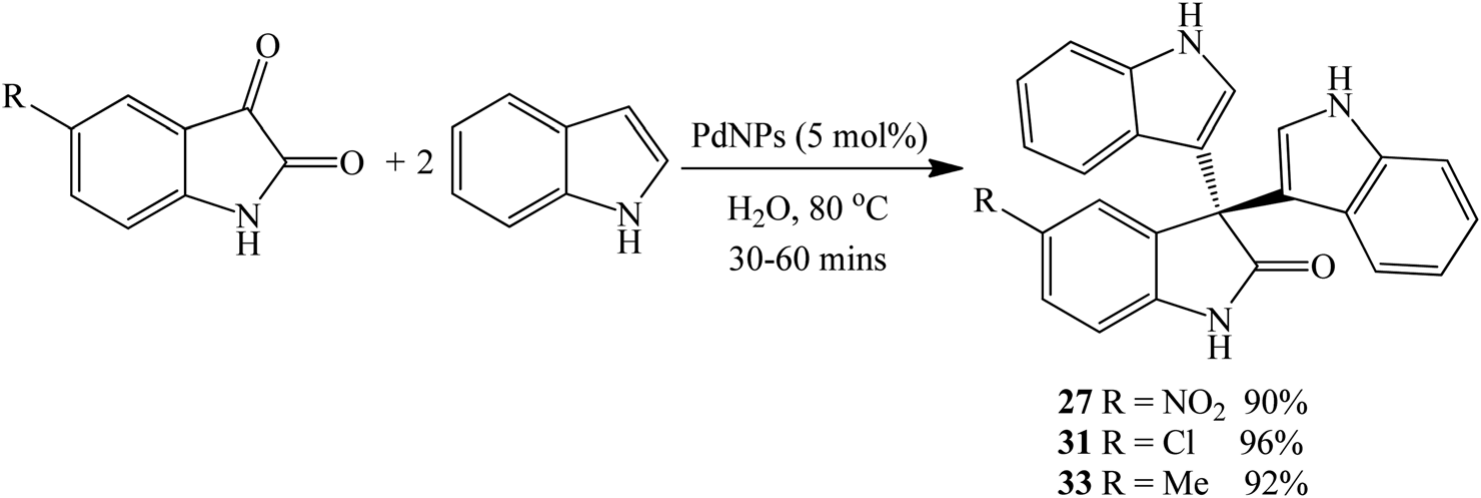
PdNPs-catalyzed synthesis of di(indolyl)indolin-2-ones 27, 31 and 33.

Nanoparticle metal catalysts are attractive reagents for organic reactions because of their ability to promote faster reaction rates, enhance catalytic activity, recyclability and increase chemical yields. With their nano-sized particles, they are characterized by voluminous surface area.^83^ Edayadulla *et al.* (2015) evaluated the palladium nanoparticles (PdNPs) for the multi-component synthesis of di(indolyl)indolin-2-ones. The PdNPs (20–30 nm particle size) were obtained by the reduction of palladium chloride (PdCl_2_) using *Artemisia annua* leaf extracts as natural reductant. Thus, indole 1 was reacted with 5-substituted isatins (5-methylisatin, 5-chloroisatin and 5-nitroisatin) in water at 80 °C for 30–60 minutes to afford the corresponding products 33, 31 and 27 in 92%, 96% and 90% yields, respectively (Scheme 30). Optimal chemical yields were obtained in the presence of 5 mol% PdNPs and the catalyst was effective with both, electron-rich and electron-deficient isatin derivatives.^84^ A prominent feature associated with this methodology involves a simple filtration workup procedure where the catalyst is recovered and recycled up to 5 times and the product is isolated without the need for further purification by column chromatography.

**Fig. 5:**
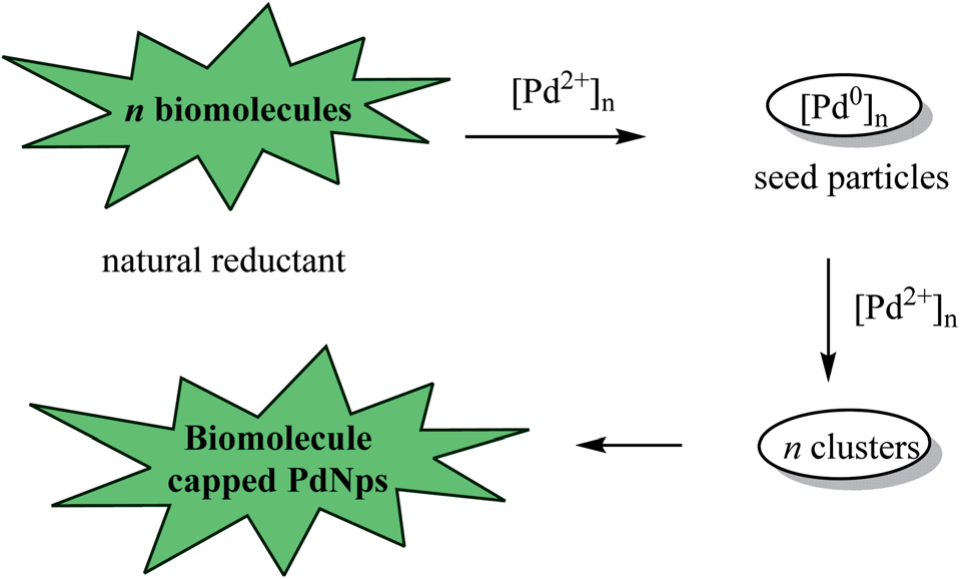
Proposed mechanism for the synthesis of PdNPs.

The mechanism for the generation of biomolecule capped Pd nanoparticles is shown in Fig. 5. The biomolecules in the aqueous *Artemisia annua* leaf extract coordinate with the Pd^2+^ ions to produce metal complexes, which are subsequently reduced to seed Pd^0^ particles. The seed particles agglomerate to clusters, which serve as nucleation centres where remaining metal ions get reduced catalytically (Fig. 5).

##### 3.1.7.2 Nickel oxide nanoparticle catalyzed reactions

**Scheme 31:**
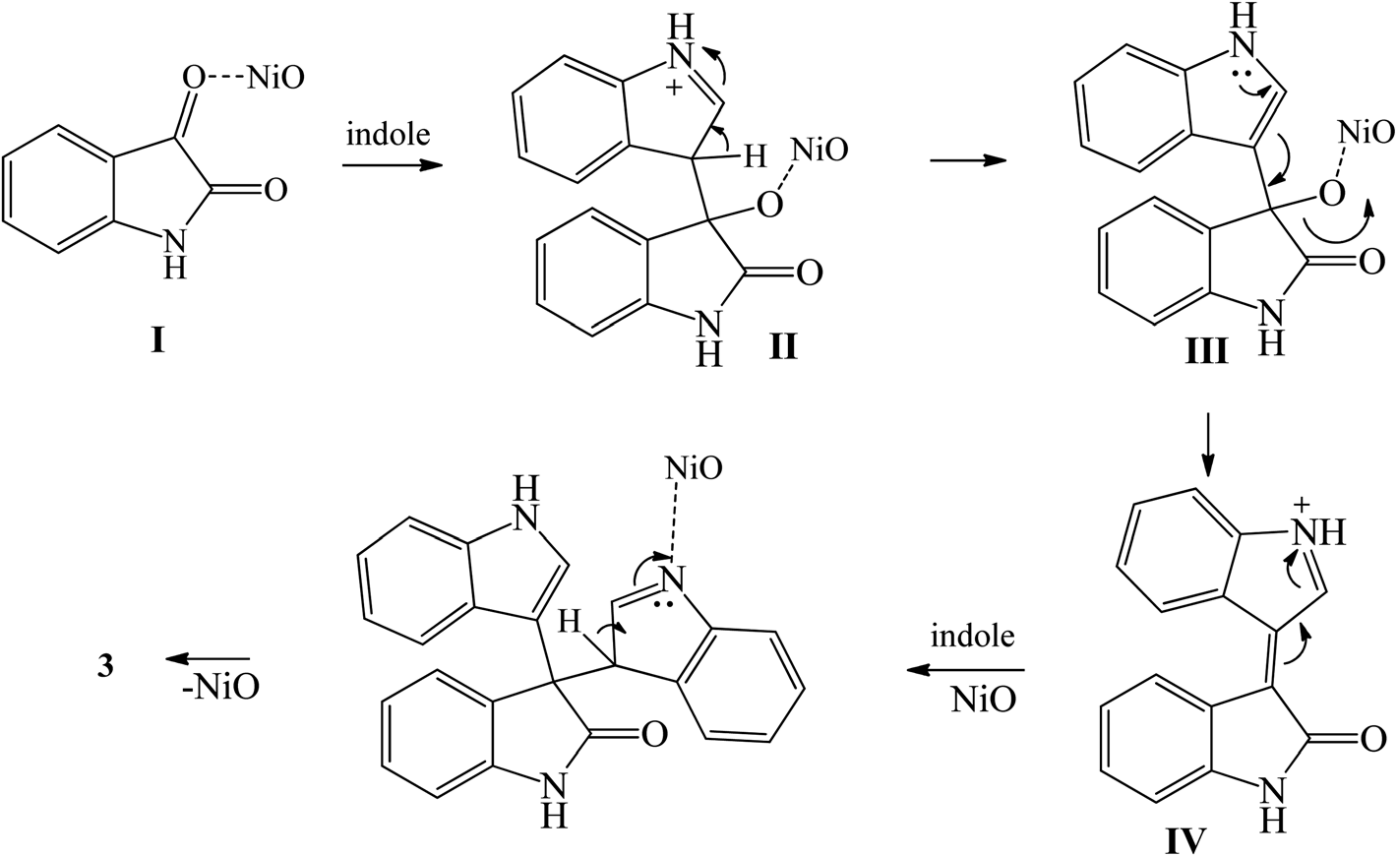
Proposed reaction mechanism for the synthesis of trisindoline 3 induced by NiO Nanoparticles.

Nasseri *et al.* (2015) used nickel oxide nanoparticle (average particle size diameter of 11 nm) to catalyze the synthesis of 3,3-diindolyloxindoles by the condensation of indoles with isatin derivatives in water at 70 °C for 0.5–1.5 h.^85^ The reaction was best catalyzed using 0.004 g NiO for every mmol of isatin. NiO nanoparticles were prepared *via* reaction of nickel(ii) nitrate hexahydrate with urea in water under heating conditions (115 °C, 1.5 h), followed by calcination at 400 °C for 1 h. The use of nano NiO was advantageous in improving the yield of 1 to 98%, compared to bare NiO (45% yield) and various other catalytic systems (10–68% yield). Furthermore, the NiO catalyst could be recycled at least five times. The optimized reaction conditions gave fair to excellent yields of 3,3-diindolyloxindoles 3, 5, 14, 18, 22, 24, 25, 27, 85, 102 and 149–152 (60–98%). Isatin derivatives bearing electron withdrawing group on C-5 showed higher yield and shorter reaction time than those with EDG at the same position. *N*-Benzylisatin was less reactive, especially upon reacting with unsubstituted indole. The proposed mechanism for the synthesis of 3 from isatin and indole catalyzed by NiO NPs is shown in Scheme 31. Initially, the activated isatin I react with indole 1 to produce intermediate II. This intermediate undergoes elimination reaction *via*III to give intermediate IV, which upon addition reaction with the second indole 1 generates trisindoline 3 (Scheme 31).^85^

##### 3.1.7.3 Titanium dioxide nanoparticle catalyzed reactions

Titanium dioxide nanoparticles (TiO_2_ NPs) were also evaluated for synthesis of trisindoline. Dwivedi *et al.* (2018) achieved the synthesis of trisindolines 3, 5, 15, 25, 27, 31, 32, 37, 86, 99, 100, 101, 103, 153 and 154 by reacting a mixture of indoles and isatins using 10 mol% nano TiO_2_ (40–70 nm) under ambient conditions. While quantitative yields for a model reaction was obtained in water as solvent, organic solvents performed relatively poorly, producing less than 80% yields after 1.5 h. The optimized conditions tolerated a wide scope of substrates with yields ranging from 95–99%. All isatins with EDG or EWG substituents on C-5 were reactive as well as those containing halogens on C-5 of the indole unit. Allyl or benzyl substituents attached to *N*-isatin barely affected the yields.^72^ It is noted though, 5-cyano, *N*-methyl, and azo indoles failed to generate the desired products.

##### 3.1.7.4 Magnesium aluminate (MgAl_2_O_4_) nanoparticle catalyzed reactions

**Scheme 32:**
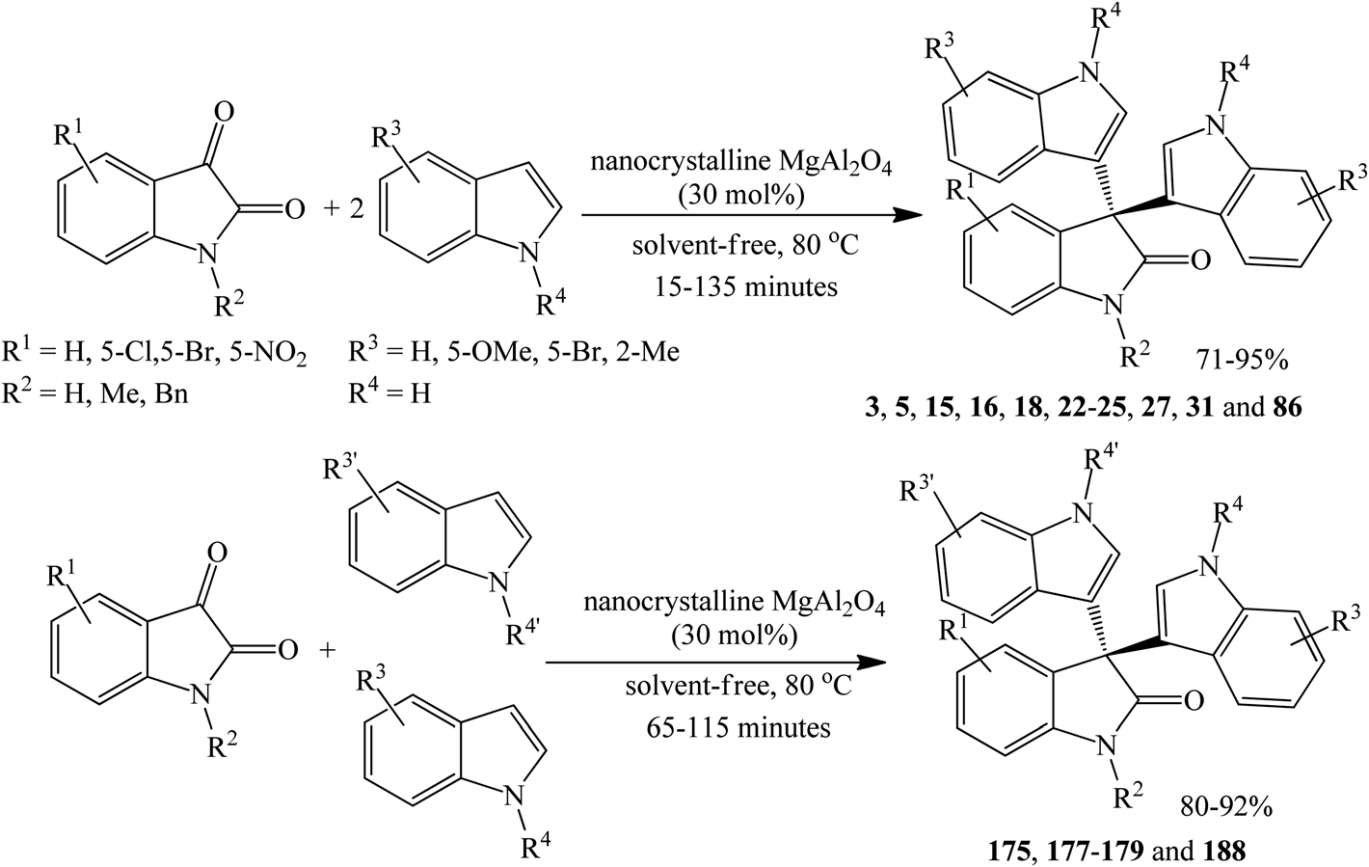
Preparation of trisindolines catalyzed by nanocrystalline MgAl_2_O_4_.

**Scheme 33:**
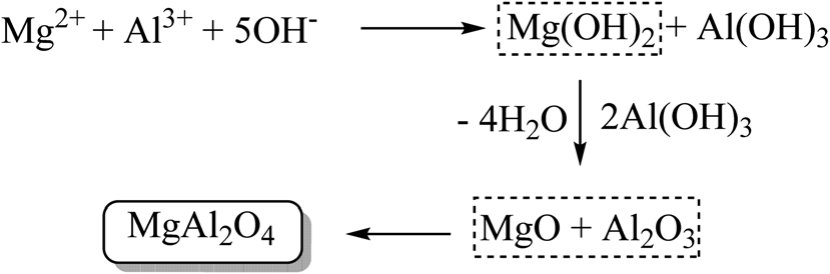
Preparation of MgAl_2_O_4_ catalyst.

Nikoofar *et al.* (2019) utilized nanocrystalline MgAl_2_O_4_ (40–50 nm particle diameter) as a recyclable catalyst for the synthesis of symmetrical trisindolines 3, 5, 15, 16, 18, 22–25, 27, 31 and 86 as well as unsymmetrical trisindolines 175, 177–179 and 188 yielding the corresponding products in 71–95% and 80–92% yields, respectively (Scheme 32). The catalyst may be prepared on a gram scale, although the lengthy procedure uses concentrated ammonia. The use of various organic solvents or water produced 56–64% yields with no prospect for yield improvement even under reflux conditions. However, the best yields were realized with 30 mol% catalyst under solvent-free conditions at 80 °C for 15–130 minutes and only 21% yield of 3 was obtained in the absence of any catalyst. While preparing 3-hydroxy-3-indolyl-indolin-2-ones involved using equimolar amounts of indoles and isatins substrates, unsymmetrical products 175, 177–179 and 188 were simply prepared by using a 1 : 1 equimolar mixture of two different indoles with the appropriate matching total molar amount of isatins (Scheme 32). It is noteworthy that the preceding one-step, three-component condensation for the preparation of unsymmetrical oxindoles derivatives from indoles and isatins has rarely been reported in the literature and no by-products of symmetrical oxindoles were observed. Various indoles and isatins with EDGs and EWGs underwent successful condensation. The substituent on isatin (H, CH_3_, Bn) had no noticeable effect on the yield or regiochemistry. Interestingly though, blocking the C-3 position forced the condensation reaction to occur at the less reactive C-2 position.^86^ The preparation of MgAl_2_O_4_ catalyst is shown in Scheme 33.

##### 3.1.7.5 Cupric tungstate (CuWO_4_) nanoparticle catalyzed reactions

**Scheme 34:**
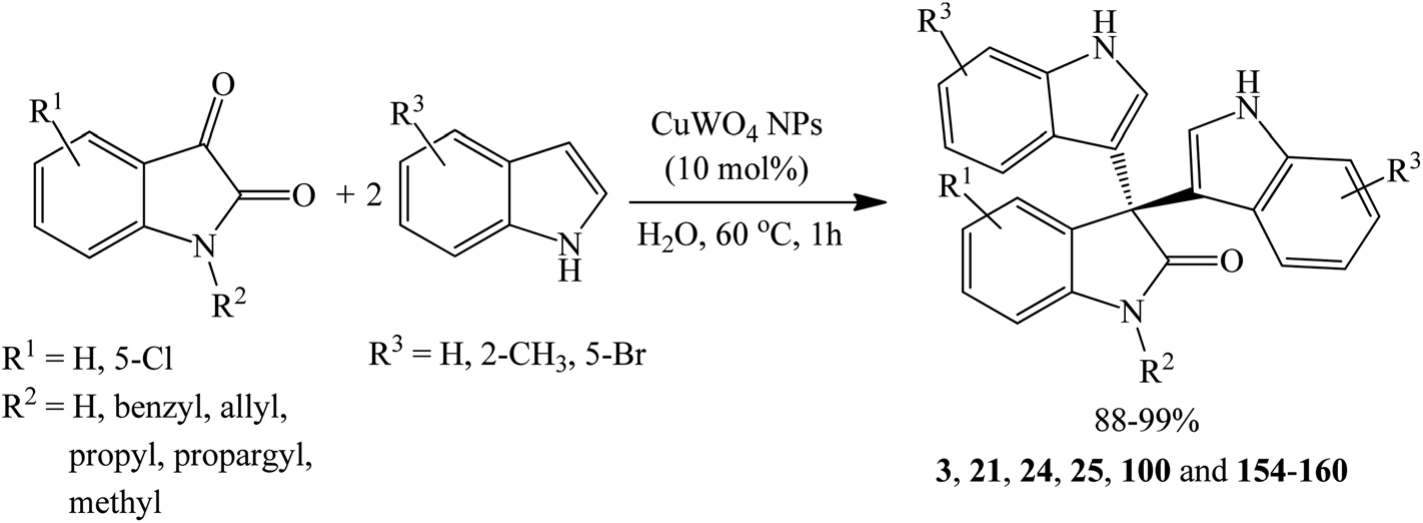
CuWO_4_ NPs-catalyzed synthesis of symmetrical di(indolyl)indolin-2-ones.

**Scheme 35:**
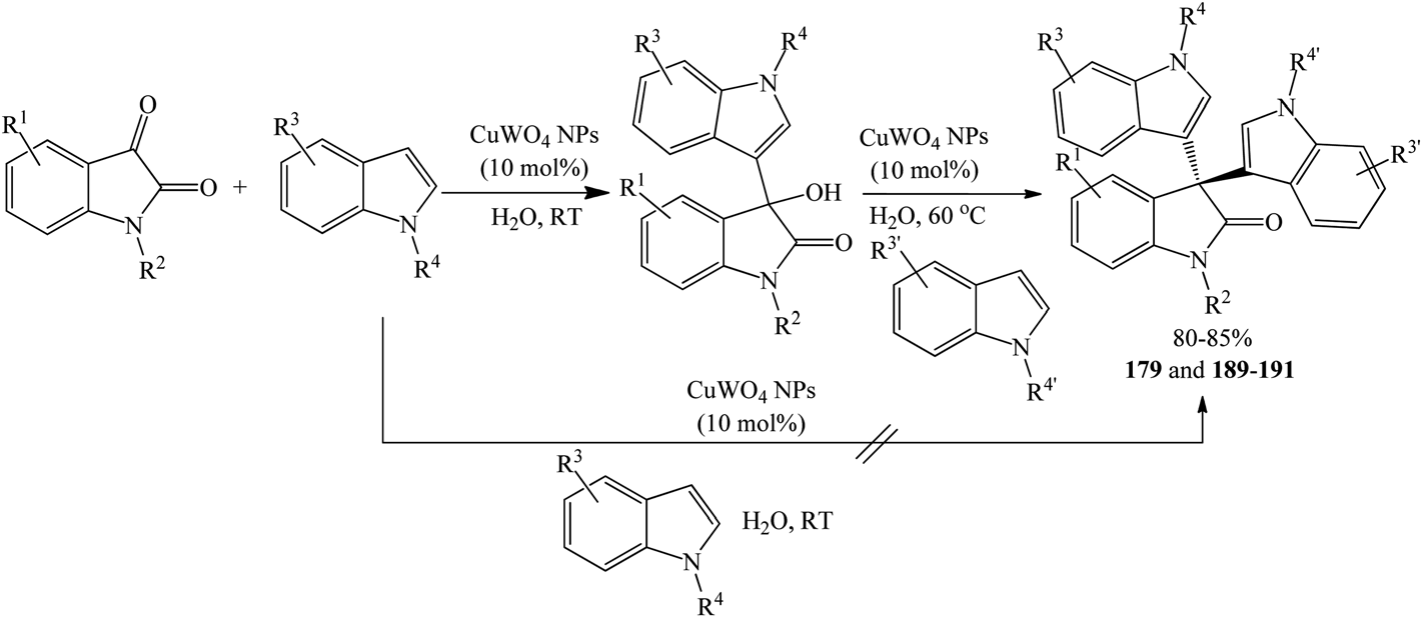
CuWO_4_ NPs-catalyzed synthesis of unsymmetrical di(indolyl)indolin-2-ones.

Paplal *et al.* (2020) synthesized a series of symmetrical trisindolines 3, 21, 24, 25, 100 and 154–160 by treating isatins and indoles with CuWO_4_ nanoparticle catalyst (10 mol%) in water at 60 °C for approximately 1 h (Scheme 34). Reaction temperature was critical to the preparation of the trisindolines as room–temperature reactions led to the formation of mono-substituted indolinones (3-hydroxy-3-(indol-3-yl)indolin-2-ones) as sole products. However, this notable feature was exploited to prepare unsymmetrical 3,3′-bis-indolyl-2-oxindoles in good yields (80–85%) (Scheme 35). As such, running controlled experiments with various starting indoles to produce mono-substituted products at room temperature, followed by further treatment with another indole at 60 °C gave unsymmetrical 3,3′-bis-indolyl-2-oxindoles 179 and 189–191 (80–85% yields). In this study, the reactivity of *N*-substituted isatin, 5-bromo, and 2-methylindole was explored. Trisindolines 3, 21, 24, 25, 100 and 154–160 were obtained in excellent yields (88–99%), whereas trisindoline 3 (R^1^–R^3^=H) was isolated in highest yield (99%) (Scheme 34). The substituents on the *N* atom of isatin ring (*N*-benzyl, *N*-allyl, *N*-propyl, *N*-propargyl, *N*-methyl) and the bromo group attached to C-5 position of indole did not impact the reactivity of substrates or yield of the products. The catalyst was recycled up to 6 cycles without loss of the catalytic activity. XRD analysis of the recovered CuWO_4_ after six cycles revealed no change in its morphology suggesting potential for re-use.^87^

##### 3.1.7.6 Zinc oxide (ZnO) nanoparticle catalyzed reactions

**Fig. 6:**
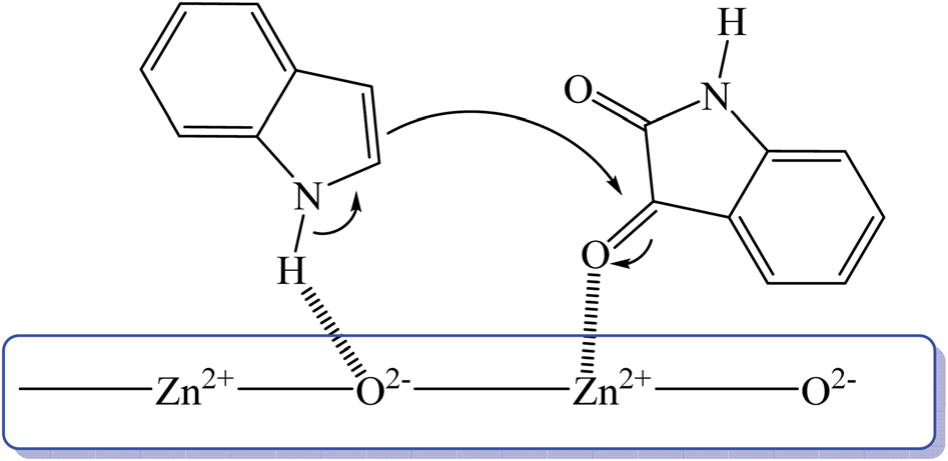
Interaction of ZnO catalyst isatin 2 with indole 1.

Nano zinc oxide (ZnO) has been exploited as catalyst for many organic transformations. Since the surface of nanostructured-ZnO consists of both Lewis acid (Zn^2+^) and Lewis base (O^2−^) sites, it is suitable for catalyzing organic reactions (Fig. 6). Recently, Zn^2+^ located on the surface of ZnO disc has been shown to play important role in activating the C-3 carbonyl group of isatin to induce 3-indolylation. To test the importance of surface catalyst, ZnO was covered with hydrophobic stearic acid and the results showed that the coated ZnO-catalyzed reaction did not generate the desired product. Nanodisc ZnO was utilized to prepare a series of trisindolines under solvent-free system at 100 °C within 2 h to produce the desired trisindolines 3, 16–18 and 54 in 82–88% yields. Interestingly, the reaction of isatin with deactivated indole (bearing cyano group) displayed higher 86% yield compared to the reaction with activated indole (bearing methoxy substituent). *N*-Methylindole or *N*-methylisatin gave 83% and 88% yields, respectively.^88^

#### 3.1.8 Supramolecular catalysts

##### 3.1.8.1 Sulfonated β-cyclodextrin (β-CD-SO_3_H) catalyzed reactions

**Scheme 36:**
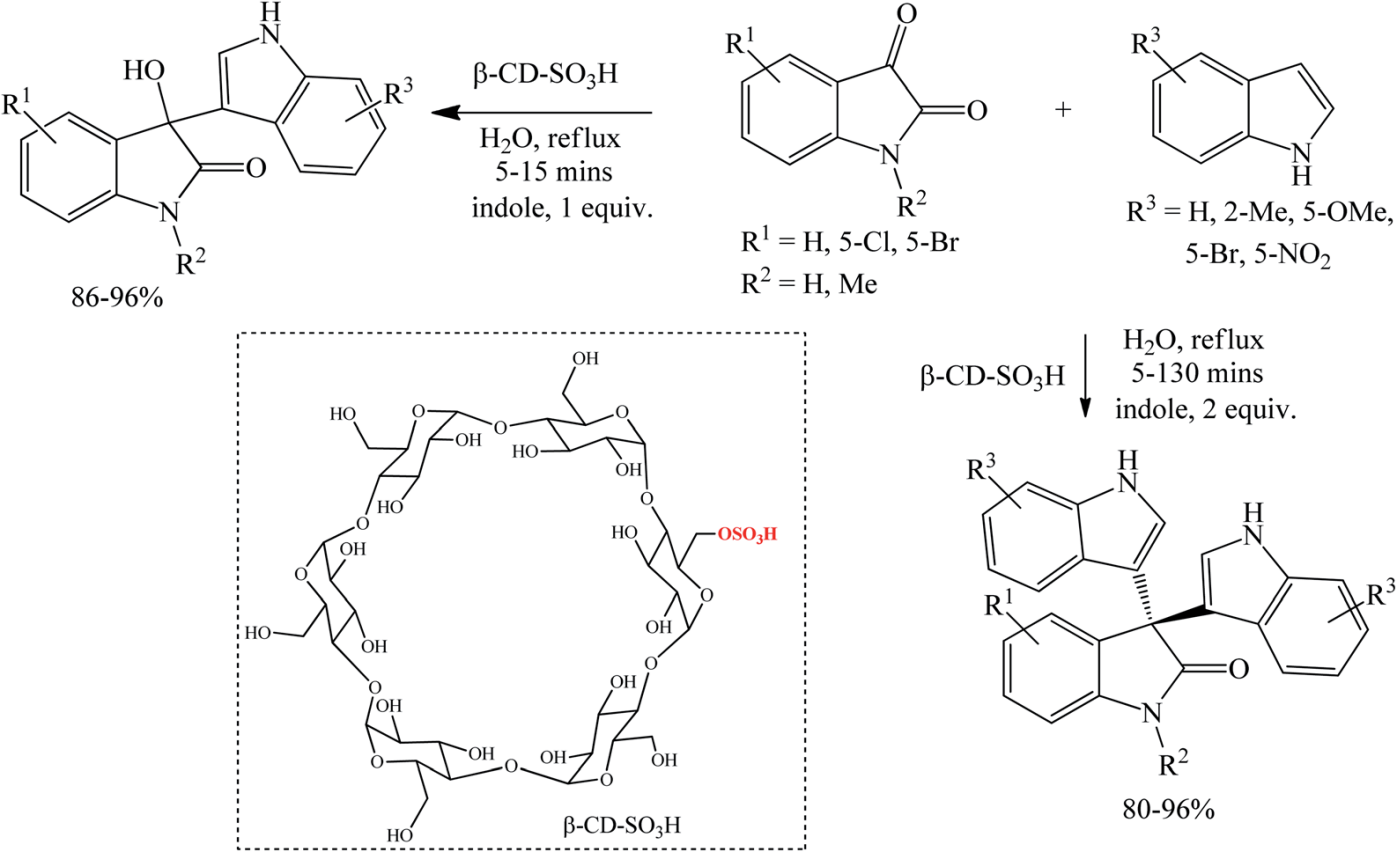
Sulfonated β-cyclodextrin-catalyzed synthesis of 3-hydroxy-3-indolylindoline-2-one and trisindolines.

Supramolecular catalysts are known to accelerate reactions by bringing the two reactants near one another and by reducing activation energy and stabilizing the transition state of the reaction. Tayade *et al.* (2015) synthesized trisindoline 3, 5, 15, 16, 18, 20–22, 31, 42, 62, 86, 88, 111 and 112 by refluxing isatins and indoles in water catalyzed by sulfonated β-cyclodextrin (β-CD-SO_3_H) for 5–130 minutes (80–96%) (Scheme 36). The yields were modest to excellent where various substrates reacted completely within 5–45 minutes, except for sluggish 5-nitroindole which required 130 minutes for complete conversion. Indole substrate with electron donating group had slightly better yield than that with halogen group (EWD). The presence of 5-methoxy group on indole, a strong electron donating group, accelerated the reaction (complete conversion within 10 minutes) and had an opposite effect to the 5-nitro group. 5-Bromoisatin was comparably less reactive than 5-chloroisatin, conceivably due to the steric factors associated with the bulky size of the bromo group. Sulfonated β-cyclodextrin displays better solubility in water than β-cyclodextrin owing to the polar sulfonate and hence shows enhanced reactivity profile. β-CD-SO_3_H also successfully catalyzed the synthesis of 3-hydroxy-3-indolylindoline-2-ones in water at reflux temperature (86–96% yield). These intermediate products are useful for the synthesis of unsymmetrical trisindolines.^89^

##### 3.1.8.2 β-Cyclodextrin catalyzed reactions

β-Cyclodextrin catalyzed the condensation reaction between isatin and indole in aqueous solvent at 80 °C within 20–100 minutes giving the trisindolines 3, 15–17, 22 and 105 in 75–98% yields. Reactions using 2-methyindoles gave 96% yield of trisindoline 22 within a shorter reaction time of 20 minutes while 2-carboxylindoles provided the lowest 75% yield of 105 and required the longest reaction time of 100 minutes. Unsubstituted trisindoline 3 gave the highest isolated yield with fastest reaction time. Interestingly, indoles bearing EDGs such as methoxy or methyl, and EWG such as bromo gave similar yields. The catalyst was recycled five times without loss of catalytic ability.^90^ The interaction between β-cyclodextrin and the starting materials gave β-cyclodextrin-substrate complexes through non-covalent bond interactions.^90,91^

##### 3.1.8.3 Sulfonated polyethylene glycol (PEG-OSO_3_H) catalyzed reactions

Sulfonated polyethylene glycol (PEG-OSO_3_H) (10 mol%) catalyzed synthesis of trisindolines 3, 5, 15, 18, 21–25, 27, 31, 84–88, 99 and 100 in acetonitrile at room temperature for 1.5–5 h, affording 65–98% yields. *N*-Methyl- or *N*-benzyl-isatins gave lower yields compared to others, especially when treated with 5-bromoindole. Consistent with what has been observed with other catalysts, isatin bearing a 5-nitro group required the longest reaction time and afforded the highest yields. PEG-OSO_3_H catalyst could be re-obtained and re-used with no significant loss of catalytic activity up to five cycles.^92^

#### 3.1.9 Magnetic nanoparticle-based catalysts

Catalysis using magnetic materials has recently attracted intensive research as it offers facile separation of catalysts that exhibit magnetic properties by using external magnet, avoiding filtration, centrifugation, or other techniques to separate the reusable catalyst.^93^ As such, nano magnetic sulfonic acid-supported Fe_3_O_4_ catalyzed the synthesis of 3,3-di(3-indolyl)-2-indolone 3 in ethanol under reflux for 3 h, providing 92% yield. The high catalytic activity of Fe_3_O_4_–OSO_3_H might presumably be attributed to the combined synergetic action of Fe_3_O_4_ as a Lewis acid and sulfonic acid as Brønsted acid.^94^

**Scheme 37:**
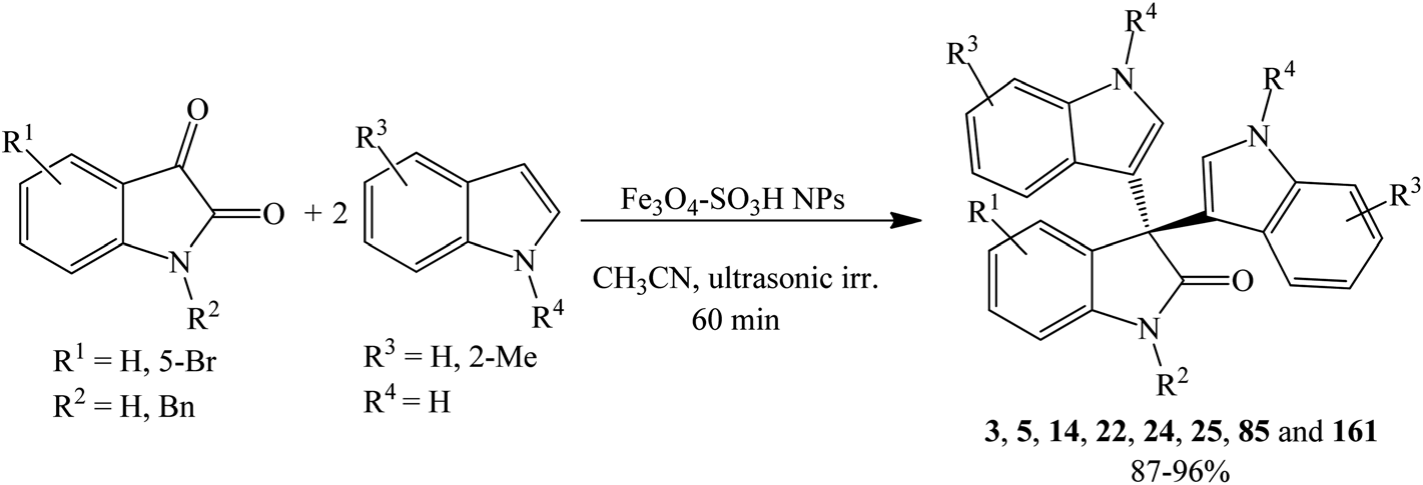
Fe_3_O_4_–SO_3_H NPs-catalyzed synthesis of trisindolines 3, 5, 14, 22, 24, 25, 85 and 161.

**Fig. 7:**
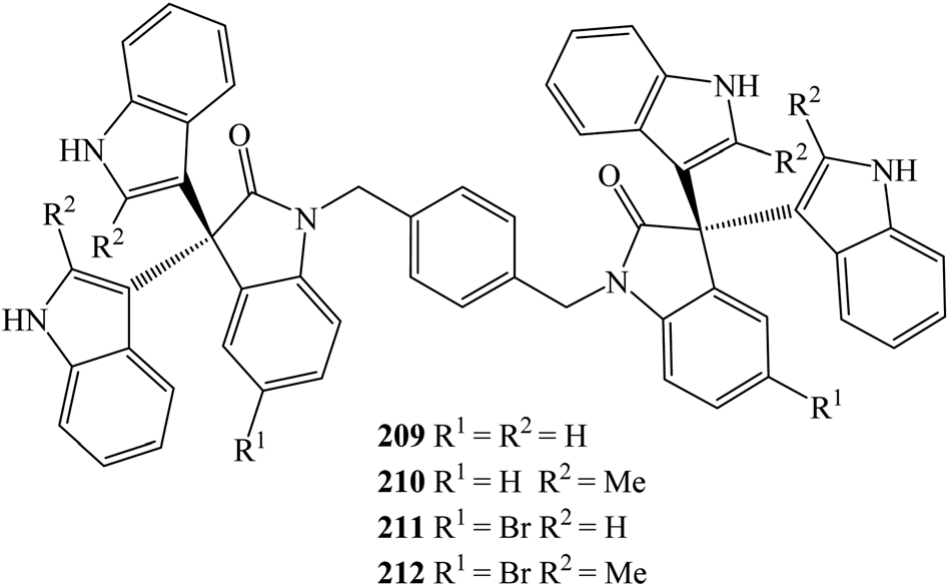
Bis-[3,3-di(indolyl)indolin-2-ones] 209–212.

Karimi *et al.* (2015) also employed nanoparticles of magnetically separable sulfonic acid-embedded Fe_3_O_4_ (Fe_3_O_4_–SO_3_H) to prepare a series of trisindolines 3, 5, 14, 22, 24, 25, 85 and 161. The magnetic nanoparticles were accessible *via* a one-step procedure by directly reacting chlorosulfonic acid with Fe_3_O_4_. Mono and bis-[3,3-di(indolyl)indolin-2-ones] 3, 5, 14, 22, 24, 25, 85 and 161 (Scheme 37) and 209–212 were obtained under ultrasonic irradiation in acetonitrile for an hour in 87–96% yields (Fig. 7). The optimum amount of catalyst was 0.15 g mol^−1^ isatin^95^. 5-Bromo substituted isatin improved the yield, while 2-methyl on indole and/or benzyl on *N* atom of isatin reacted smoothly to produce good yields. Generally, compound 3 was obtained in 93% yield within lower reaction time compared to other methods^94^ possibly because ultrasonic irradiation promoted faster and higher yielding reactions.^95^

Another fabricated supported-Fe_3_O_4_@SiO_2_ MNPs, a carboxylic acid-embedded Fe_3_O_4_@SiO_2_ (Fe_3_O_4_@SiO_2_@COOH), gave trisindolines 3, 5, 18, 22, 23, 85, 162 and 163 in 60–98% yields at 80 °C in aqueous solvent within 30–60 minutes. As many reports utilizing ferrite-silica (Fe_3_O_4_) based catalysts, 5-bromoisatin was excellent electrophile. Furthermore, it produced the highest yield when coupled with the highly nucleophilic 2-methylindole. EDG (methyl, morpholinomethyl) attached on *N*-isatin reduced the reactivity. The catalyst possesses high density of acidic sites and magnetic behaviour.^96^

Fe_3_O_4_@SiO_2_@Bi_2_O_3_ MNPs was used to prepare trisindolines 3, 5, 14, 18, 22–25, 85, 151 and 164 in 65–97% yields within 25–90 minutes. Methyl or benzyl attached to *N*-isatin render it less electrophilic, promoting slower reactivity and requiring prolonged reaction time to improve the yield. However, the presence of 5-bromo (as electron withdrawing group) on isatin ring led to a remarkably improved yield.^97^

Gupta *et al.* (2019) fabricated silica-coated magnetite-nanoparticle anchored DABCO-derived and acid-functionalized ionic liquid (DABCO-3@FSMNPs) and applied it as recyclable nanocatalyst to prepare a library of trisindolines. The reactions were carried out in H_2_O at 90 °C for 2 h to afford products 3, 5, 15, 16, 31, 32, 42, 60, 62, 86, 101 and 111 in 85–98% yields. Both electron-deficient and electron-rich indoles reacted well with either isatin or halo-substituted isatin. However, 2-methylindole was inert and did not afford any products.^98^ The three-component reaction was found to proceed exclusively *via* H-bonding intermolecular interactions between isatin and indole substrates and the nanocatalyst. The catalyst could be recycled easily without any significant loss in catalytic activity.

**Scheme 38:**
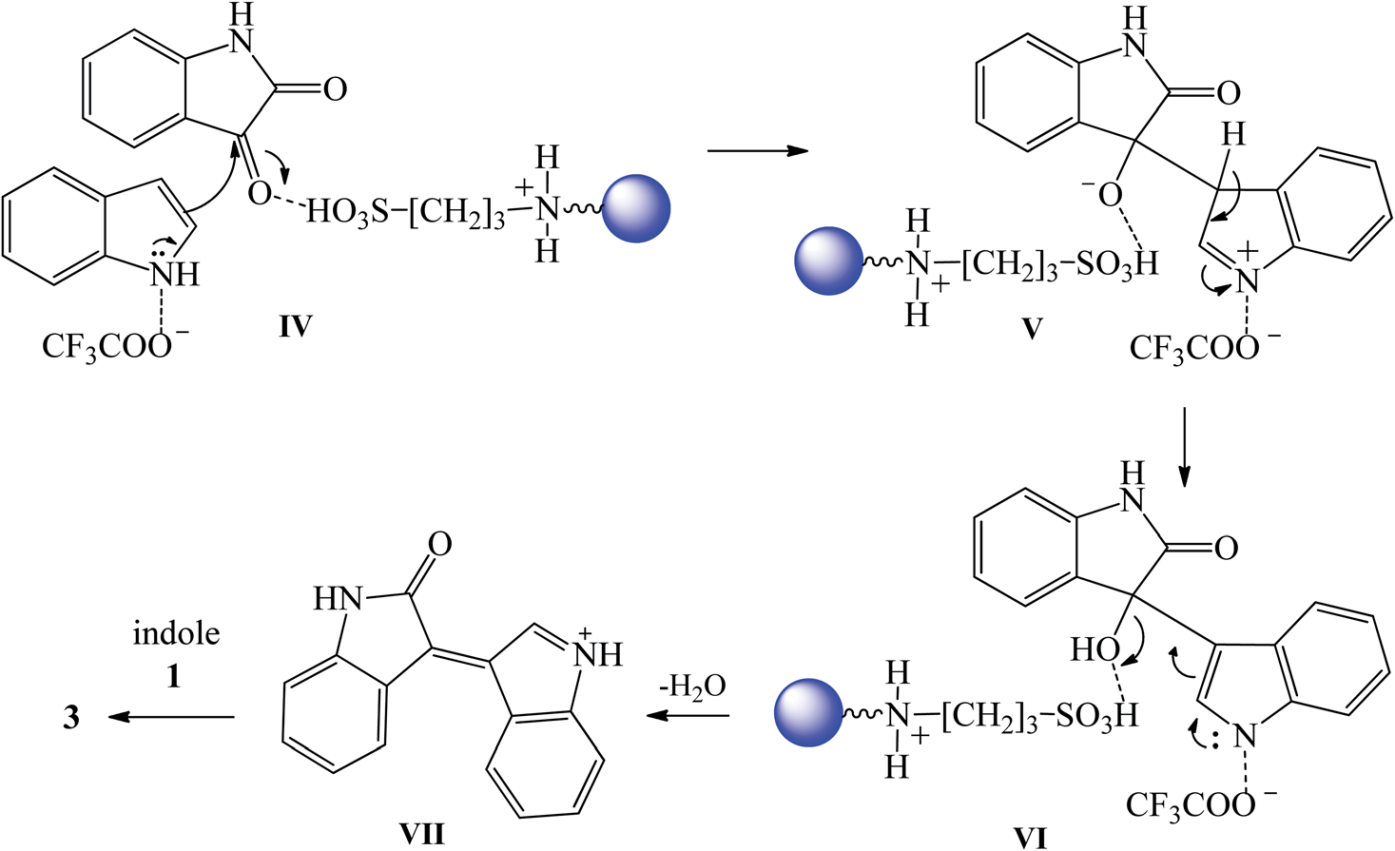
The proposed mechanism of trisindoline 3 synthesis catalyzed by DABCO-3@FSMNPs.

The plausible pathway of trisindoline formation catalyzed by DABCO-3@FSMNPs is illustrated in Scheme 38. Hydrogen bonds are formed by the interaction of both cationic and anionic species of the ionic liquid catalyst with the reactants (indole and isatin) (IV). The carbonyl (C-3) of isatin 2 is activated by the proton-donor of the sulfonic acid, making it more electrophilic. At same time, indole 1 is more nucleophilic due to the participation of trifluoroacetate as proton acceptor from the N–H of indole 1. Hence, it allows the nucleophilic attack of C-3 indole 1 onto activated carbon of C-3 isatin 2 to render V. Intermediate VII, generated by the dehydration of V to the corresponding α,β-unsaturated iminium ion, is attacked by a second indole 1 molecule to produce trisindoline 3.

#### 3.1.10 Nanocomposite materials based catalysts

##### 3.1.10.1 TiO_2_-impregnated SiO_2_ catalyzed reactions

Nanocomposite materials possess large surface areas and unique properties when compared to free nanomaterials. These materials consist of a matrix (graphene or graphene-like) and fillers, which can be metals, metal oxides, *etc*^99^. Haghighi and Nikoofar (2014) employed nanocomposite TiO_2_-impregnated SiO_2_ as a Lewis acid to catalyze the synthesis of a series of symmetrical trisindolines under neat conditions at 50 °C for 15–120 minutes, producing the corresponding products 3, 5, 15, 17, 18, 22–25, 27 and 28 in 72–93% yields. The catalyst is prepared by mixing column chromatography grade SiO_2_ 60 in CHCl_3_ with nano TiO_2_ and stirring at room temperature for 1.5 h. Simple evaporation of the solvent at room temperature affords 50% (w/w) nano TiO_2_/SiO_2_ as white solid. The reaction of 2-methylindole and unsubstituted isatin exhibited the fastest reaction time and the highest yield (93%). Bromo substituent attached to C-5 of either isatin or indole gave longer reaction and the lowest product yield. *N*-benzylated and 5-Br, 5-NO_2_-isatins were less reactive and required longer reaction times for complete conversion. Under similar reaction conditions, the catalytic activity of non-supported free nano TiO_2_ (70% yield) or SiO_2_ (60% yield) did not afford better yields of 3 in comparison with nano TiO_2_@SiO_2_ (85% yield).^100^

##### 3.1.10.2 Al_2_O_3_/V_2_O_5_ nanocomposite catalyzed reactions

Hassani *et al.* (2017) used Al_2_O_3_/V_2_O_5_ nanocomposite as a heterogeneous catalyst for trisindoline synthesis at 70 °C in water for 10–30 minutes to form trisindolines 3, 5, 18, 22, 23, 85, 162 and 163 in 60–98% yields. The catalyst was reusable but diminished activity was observed following multiple cycles. Reactions of substituted isatin with 2-methylindole led to decreased yields and long reaction times, but the reaction of unsubstituted isatin with 2-methylindole behaved oppositely, generating the highest observed yield of product 22.^101^ Further, 2-methylindole was most reactive compared to other substituted indoles with ferrite silica Fe_3_O_4_@SiO_2_@COOH nanoparticles catalyst.^96^

##### 3.1.10.3 SiO_2_@g-C_3_N_4_ nanocomposite catalyzed reactions

Allahresani *et al.* (2017) utilized SiO_2_@g-C_3_N_4_ nanocomposite as heterogeneous and recyclable catalyst for Friedel–Crafts reaction between indoles and isatins. The nanocomposite was prepared from the reaction of graphitic carbon nitride nanosheets produced from melamine (g-C_3_N_4_) and silicon dioxide nanoparticles by heating in a furnace at 550 °C. The synthesized trisindolines 3, 15, 17, 18, 21, 23–25, 27, 29, 31, 84, 86–88, 99, 100 and 165 were produced in high to excellent yields (81–95%) within 30–75 minutes (Table 4). Indoles with electron-releasing substituents at C-2 and isatins bearing EWG at C-5 position reacted synergistically to give the best yields. Although nano-SiO_2_ (20 mg) catalyzed the synthesis of trisindolines more efficiently than SiO_2_@g-C_3_N_4_ (60 mg), a tedious separation of nano-SiO_2_ from the reaction media as well as agglomeration of nano-SiO_2_ rendered it impractical. The remarkable catalytic behavior of SiO_2_@g-C_3_N_4_ nanocomposite has been attributed to the uniformly distributed SiO_2_ nanoparticles where size distribution centered at a value of 17.6 nm.^102^

##### 3.1.10.4 CuO@g-C_3_N_4_ nanocomposite catalyzed reactions

**Scheme 39:**
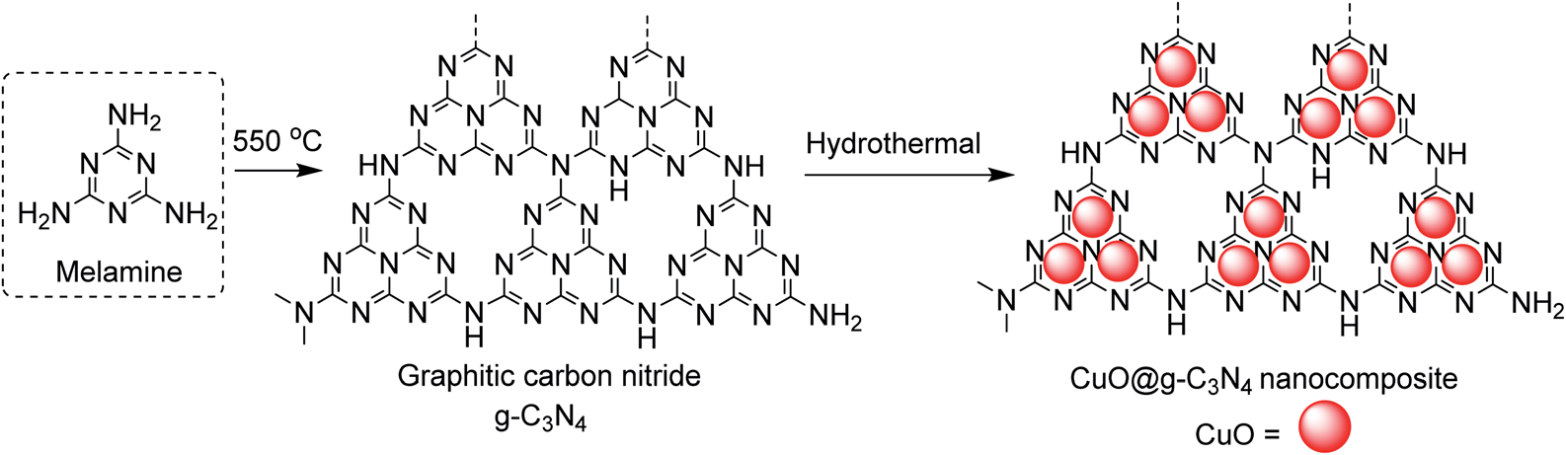
The synthesis of CuO@g-C_3_N_4_ nanocomposites.

Another doped graphitic carbon nitride catalyst, nanocomposite CuO@g-C_3_N_4,_ was also investigated and applied for trisindoline synthesis by Allahresani (2017) (Scheme 39). The reactions were conducted at room temperature in water to produce the targeted trisindolines 3, 15, 17, 18, 21, 23–25, 27, 29, 31, 84, 86–88, 99, 100 and 165 within 45–75 minutes with yields ranging from 80–95% (Table 4).^103^ The best yields were obtained with 85 mg of the catalyst per mole of isatin. The synthesis of the same trisindolines required longer time than those prepared using SiO_2_@g-C_3_N_4_. Indole bearing electron withdrawing group at C-5 position led to longer reaction time and lower yields (82–83%). The catalyst was however able to match the optimal yields obtained in ref. 102 with isatin bearing EWG on C-5 and indoles having EDG on the C-1 position. The catalytic activity of CuO@g-C_3_N_4_ has been attributed to the uniformly distributed CuO nanoparticles impregnated into the pi-conjugated graphitic carbon nitride nanosheets assist in C–C bond formation. One noted disadvantage involves the aggregation of the nanoparticles as observed in the second reuse of SiO_2_@g-C_3_N_4_ catalyst.^103^

**Table 4:** Reaction of isatins and indoles using g-C_3_N_4_–based catalysts

Trisindolines	SiO_2_@g-C_3_N_4_ (ref. 102)		CuO@g-C_3_N_4_ (ref. 103)		Fe(iii)@g-C_3_N_4_ (ref. 99)	
	Time (min)	Yields (%)	Time (min)	Yields (%)	Time (min)	Yields (%)
3	45	95	60	95	30	96
15	75	83	75	80	40	80
17	35	95	55	95	27	96
18	60	90	75	89	50	90
21	60	83	75	81	50	80
23	55	91	75	90	50	87
24	55	87	75	86	50	83
25	60	81	75	80	50	82
27	70	91	70	95	55	95
29	50	85	60	87	40	90
31	35	93	55	94	30	96
84	60	93	65	95	40	95
86	75	82	75	88	40	85
87	30	95	45	93	30	93
88	75	83	75	83	55	85
99	75	91	75	94	50	92
100	60	82	75	80	50	79
165	45	95	45	95	30	95

##### 3.1.10.5 Fe(iii)@g-C_3_N_4_ nanocomposite catalyzed reactions

Recyclable Fe(iii)@g-C_3_N_4_ is another nanocomposite catalyst Allahresani and co-workers (2018) studied for oxindoles synthesis (Table 4). Initially, g-C_3_N_4_ nanosheets were prepared by the oxidation of melamine powder in a heated furnace (550 °C) over 4 h. The catalyst is then prepared by stirring a mixture of FeCl_3_ and g-C_3_N_4_ nanosheets in EtOH at 40 °C for further 6 h. The most effective microscopic and spectroscopic characterization techniques for such nanocomposites include XRD and TEM. For instance, XRD analysis for pure g-C_3_N_4_ established the hexagonal phase of the nanosheets by two observed distinctive peaks at 13.1 and 27.4°. The signals indicate interlayer stacking and repeated units. Absence of such peaks for Fe(iii)@g-C_3_N_4_ suggests a breakdown of the nanosheet hexagonal phase, interlayer stacking, and planar repeated units due to the incorporation of FeCl_3_. On the other hand, while the TEM image of pure g-C_3_N_4_ shows the expected agglomerated nanosheet, that of Fe(iii)@g-C_3_N_4_ clearly shows the presence of FeCl_3_ throughout the surface of the nanocomposite sheets. The condensation of indoles and isatin molecules to give trisindolines 3, 15, 17, 18, 21, 23–25, 27, 29, 31, 84, 86–88, 99, 100 and 165 in 79–96% yields proceeded under reflux in water using 35 mol% Fe(iii)@g-C_3_N_4_ catalyst, and it required shorter reaction time (27–55 minutes). Trisindoline 17 was obtained in 96% yield in just 27 minutes. It is noted that among a dozen other g-C_3_N_4_-based catalysts, Fe(iii)@g-C_3_N_4_ furnished the highest yield of trisindolines.^99^

In summary, the presence of g-C_3_N_4_ support limits metal and metal oxide nanoparticles from accumulation to increase the activity and selectivity of the catalyst. Metal or metal oxide nanoparticles exhibits a problem in its aggregation, and this problem can be overcome by doping with g-C_3_N_4_.^103^ Among above g-C_3_N_4_ support catalyst, Fe(III)@g-C_3_N_4_ required shorter reaction times, and no significant differences in the yields of product was observed.

##### 3.1.10.6 MoS_2_-GCN nanocomposite catalyzed reactions

**Scheme 40:**
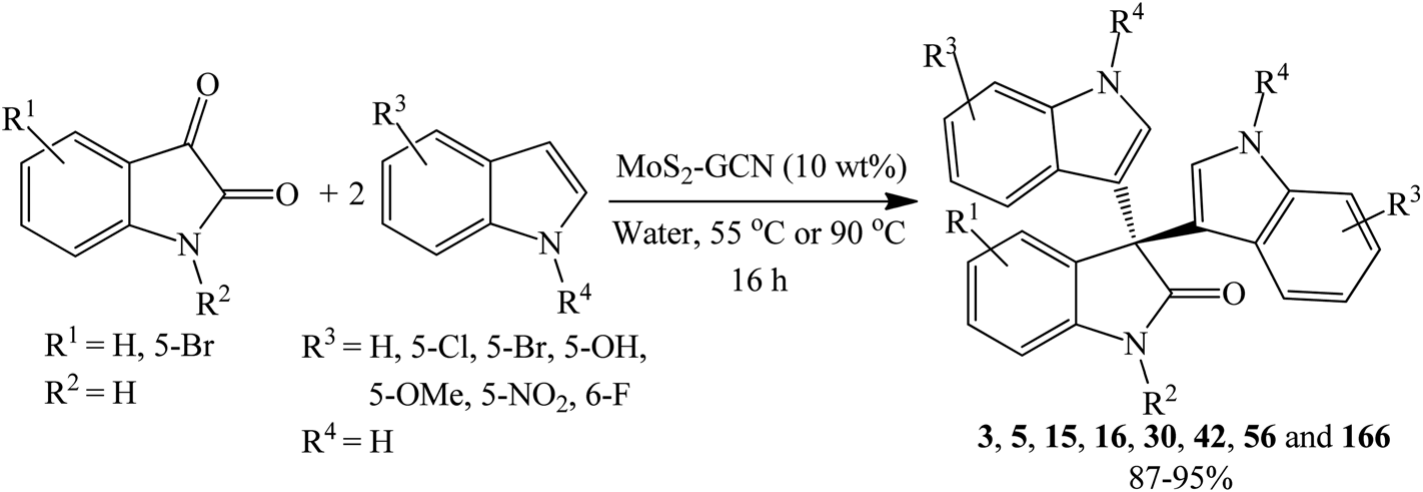
MoS_2_-GCN-catalyzed synthesis of trisindolines 3, 5, 15, 16, 30, 42, 56 and 166.

Molybdenum disulfide-supported on graphitic carbon nitride (GCN), MoS_2_-GCN, is a nanocomposite of two dimensional MoS_2_ and has been employed as catalyst in several organic reactions. While MoS_2_ acts as a Lewis acid catalyst, GCN exhibits basic character due to the nitrogen atoms. The exceptionally high catalytic activity of nanocomposite MoS_2_-GCN may be linked to the synergistic effect of non-bonding Lewis acid–base arrangement and possibly because it possesses relatively larger surface area among many other composites. Bahuguna *et al.* (2018) prepared the catalyst with varying amount of MoS_2_ in GCN nanosheets to generate MoS_2_-GCN (1 : 1), MoS_2_-GCN (1 : 2) and MoS_2_-GCN(1 : 4) nanocomposites and applied them for the synthesis of trisindoline 3. The best of the three catalytic systems was determined to be 10 wt% of MoS_2_-GCN (1 : 1). Reactions of indoles substituted by either EDG or EWG at C-5 with isatin produced notably excellent 87–95% yields of trisindolines 3, 5, 15, 16, 30, 42, 56 and 166 (Scheme 40). The 5-nitroindole substrate, however, required higher reaction temperature of 90 °C.^104^

##### 3.1.10.7 Ru(iii)-exchanged zeolite Y nanocomposite catalyzed reactions

Khorshidi and Tabatabaeian (2010) treated a mixture of indoles and isatins with Ru(iii)-exchanged zeolite Y as a catalyst (10 mol%) in 1,2-dichloroethane for 20–75 minutes to afford trisindolines 3, 15, 17, 19, 22, 54 and 167 in 60–98% yields. The catalyst was prepared from zeolite FAU-Y and ruthenium chloride hydrate by stirring a mixture thereof at room temperature for 1 day. EDG on the indoles (N atom, C-2 or C-3) enhanced the yield or contributed to faster reaction than indoles with EWG. 5-Cyanoindole reacted well with isatin and yielded 80% of 54. However, reacting 5-cyanoindole with 5-cyanoisatin resulted in the longest reaction time and lowest yield of 167.^105^ In addition, another mild synthesis of trisindoline 3 catalyzed by NaY zeolite functionalized by sulfamic acid/Cu(OAc)_2_ (NaY zeolite-NHSO_3_H/Cu(OAc)_2_) in acetonitrile for 50 minutes gave 96% yield.^106^

#### 3.1.11 Biocatalyzed reactions

**Scheme 41:**
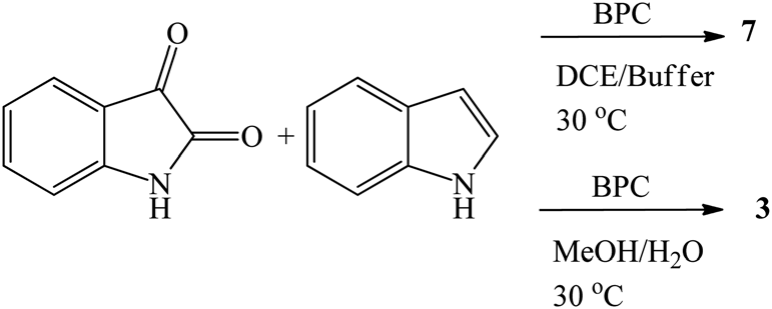
BPC-catalyzed synthesis of trisindoline 3 and 3-hydroxy-3-(indol-3-yl)-2-indolone 7.

α-Chymotrypsin from bovine pancreas (BPC) was utilized as an enzymatic catalyst for the synthesis of trisindolines by Xue *et al.* (2016). Indoles and isatins were treated with 0.93 kU of bovine pancreas (BPC) in methanol with 20% water at 30 °C for 60–96 h to yield trisindolines 3, 15–18, 20, 27–29, 32, 38, 56–66, 68 and 69 in 63–97% yields. When polar protic solvent was changed to aprotic solvent, 7 was formed instead of 3. Trisindoline 3 was obtained in 96% yield after 72 h (Scheme 41). Isatins bearing EDGs or EWGs worked well with the indole and its substituted form. Nevertheless, 5-nitroisatin and 1-methylindole were less reactive, producing relatively lower yields.^107^ In general biocatalysts require longer reaction time in comparison with other catalysts.

#### 3.1.12 Electrochemical-based reactions

**Scheme 42:**
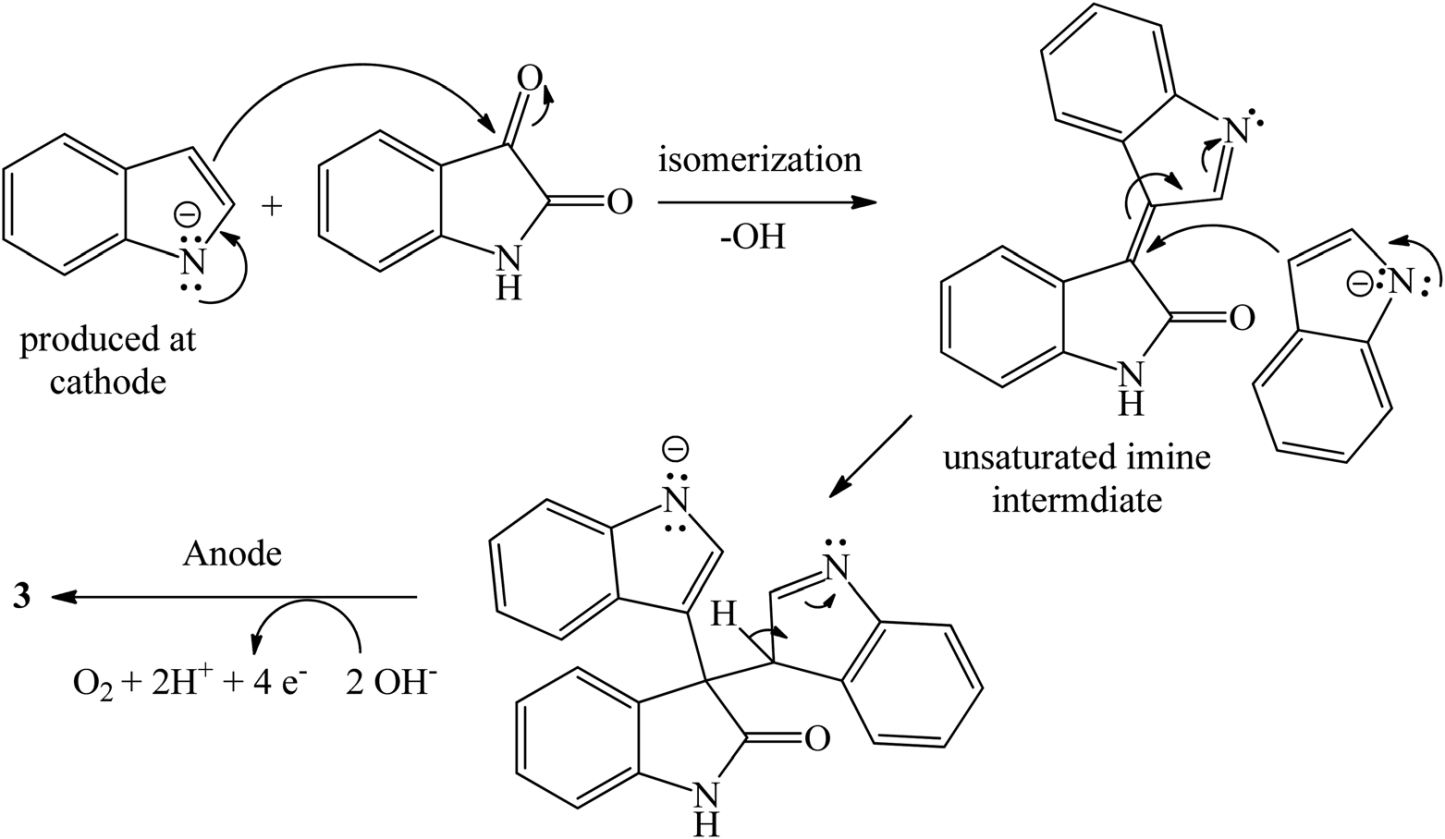
Proposed mechanism of trisindoline synthesis by electrolysis.

Trisindoline nanorods 3, 5, 15, 17, 18, 22, 25 and 27 were synthesized in excellent yields (90–96%) by electrochemical methods. Reaction of isatins and indoles were conducted in acetonitrile in unseparated cell under constant current (20 mA) at ambient temperature for 5–150 minutes. LiClO_4_ was used as electrolyte, and graphite rods as the cathode and the anode. Overall, the reactions of substituted indoles with isatin proceeded in shorter reaction times and gave better yields than those with indole and substituted isatins. Trisindoline 18 and 25 obtained from *N*-alkylated and *N*-benzylated isatins gave the highest yields of 96%. The proposed mechanism of the reaction producing trisindoline 3 is displayed in Scheme 42.^108^ Deprotonation of the indole at the cathode produces indole anion which undergoes the usual nucleophilic addition at the C_3_ carbon of isatin to produce, after proton transfer and elimination of hydroxide ion, an α,β-unsaturated imine intermediate. A second molecule of indole anion reacts with the imine, followed by protonation of the indole nitrogen by protons produced at the anode and a final isomerization step to form the product.

#### 3.1.13 Catalyst-free based synthesis

The catalyst-free reaction between isatins and indoles in water under reflux condition gave trisindolines 3, 5, 15, 22, 32, 33, 40, 85, 88, 101,104 and 115 in 80–93% yields.^109^ The synthesis was also achieved in water/ethanol (7 : 3) mixture within 5–38 minutes to give 55–86% trisindolines 3, 15, 17–19, 22, 23, 69, 153, 158 and 168–171.^110^ In both cases, the reactivity and yield of the trisindolines increased with EDG on C-5 of the indole ring and decreased in the presence of EWG such as bromine. Furthermore, substituents attached to the NH of isatins decreased the reactivity and yields.

### 3.2 Synthesis of trisindolines from isatin-imine as coupling partner

#### 3.2.1 Ru(iii)·*n*H_2_O catalyzed reactions

**Scheme 43:**
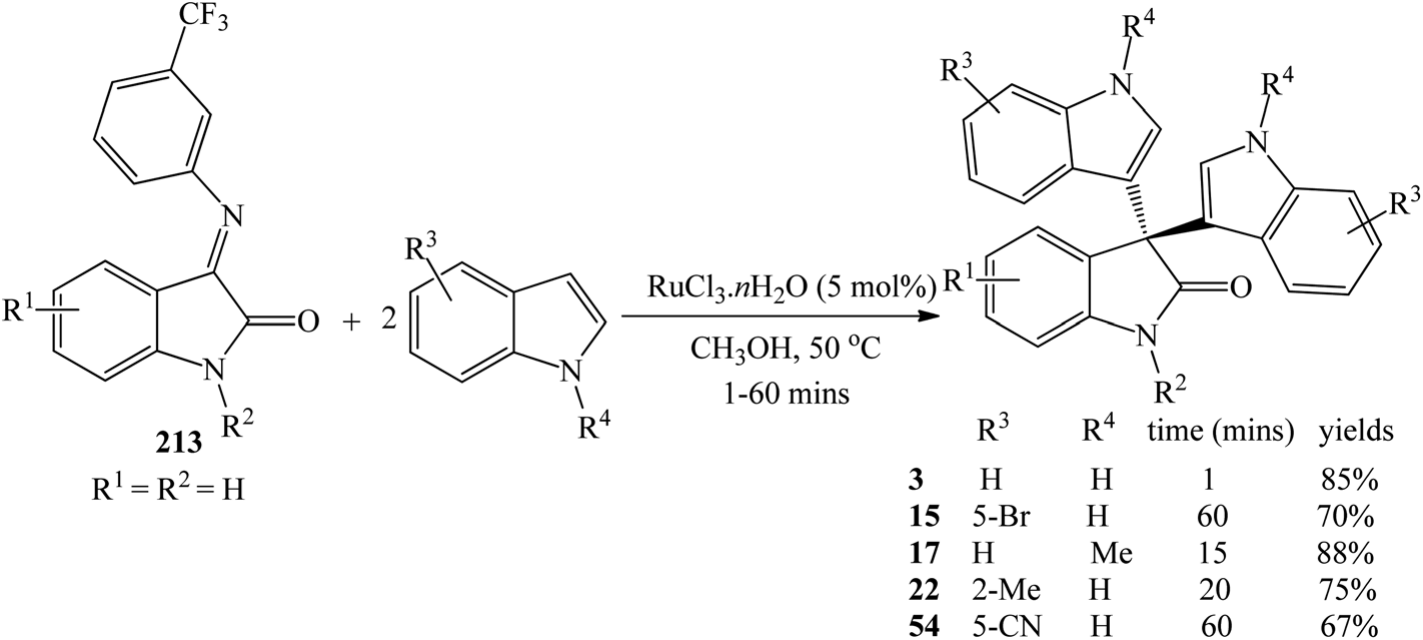
Reaction of isatin-imine with indoles in the presence of RuCl_3_·*n*H_2_O.

Ru(iii)-catalyst was also used for condensation reactions of CF_3_-attached isatin-imine 213 with several indoles in methanol for 1–60 minutes. The reactions gave trisindolines 3, 15, 17, 22 and 54 in 67–88% yields (Scheme 43). Reaction of indole and isatin gave trisindoline 3 in 85% yield in just 1 minute. EWGs retarded the reaction and gave lower yields. Accordingly, RuCl_3_ worked well in the following order: reaction of isatin and indole > reaction of isatin-derived aldimine and indole > trimerization of indole.^82^

#### 3.2.2 *p*-TsOH catalyzed reactions

**Scheme 44:**
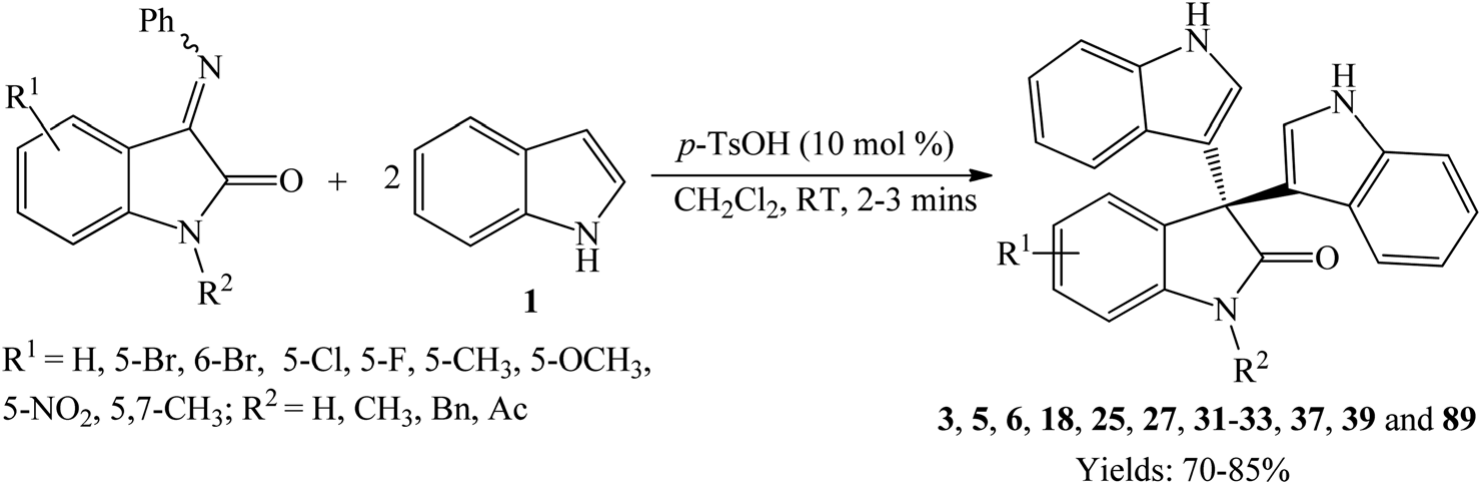
Trisindolines obtained from the reaction of isatin-imine and indoles catalyzed by *p*-TsOH.

Coupling reaction between isatin-imines and indoles using catalytic amount of *p*-TsOH (10 mol%) in dichloromethane at room temperature proceeded within 2–3 minutes. This method tolerated various indoles and isatins where trisindolines 3, 5, 6, 18, 25, 27, 31–33, 37, 39 and 89 were obtained in 70–85% yields (Scheme 44). EDG (OCH_3_, CH_3_) attached to isatin ring of (phenylimino)indolin-2-ones gave higher yields compared to the ones with EWG (F, Cl, Br, NO_2_). Moreover, the presence of acetyl group on nitrogen rendered isatin-derived aldimine less reactive than those bearing methyl or benzyl group.^111^

**Scheme 45:**
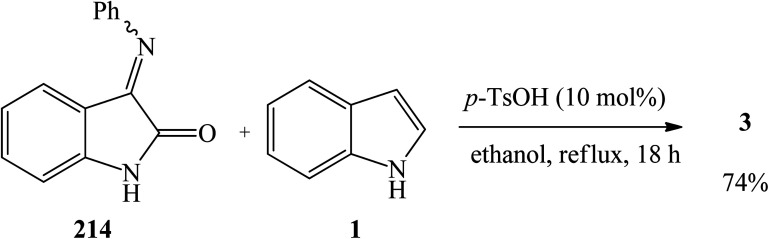
Reaction of isatin-imine with indoles in the presence of *p*-TsOH.

Schilling *et al.* (2020) reported the reaction of prepared isatin-imine 1,3-dihydro-3-(phenylimino)-2*H*-indol-2-one 214 (1 equivalent) and indole (two equivalents) in the presence of *p*-TsOH catalyst (10 mol%) under reflux in ethanol for 18 h, generating 3 in 74% yield (Scheme 45).^112^

### 3.3 Synthesis of trisindolines from 3,3-dibromoxindole as coupling partner

**Scheme 46:**
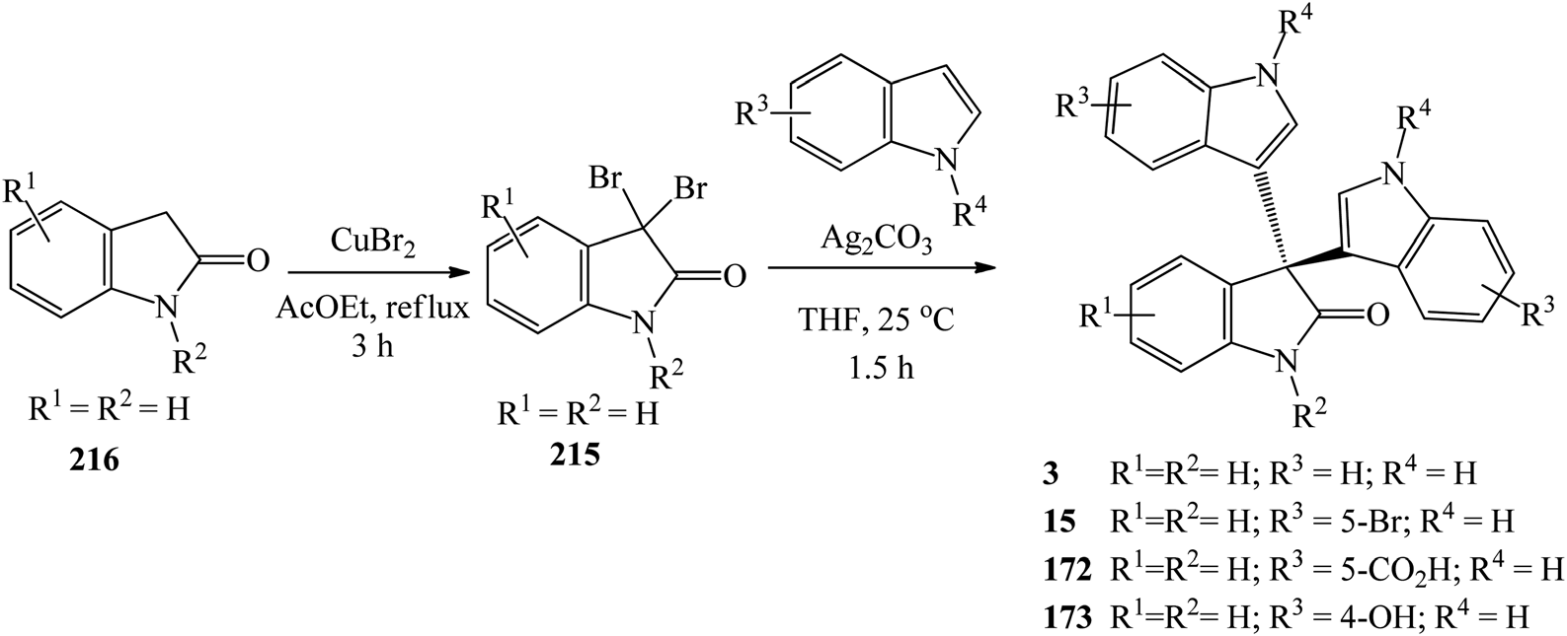
Two-step synthesis of trisindolines.

Reaction of 3,3-dibromoxindole 215 with indole, catalyzed by silver carbonate at 25 °C for 1.5 h gave trisindoline 3 in 65% yield. Following the same procedure, reaction of 3,3-dibromoxindole 215 with other indoles gave the corresponding trisindolines 15, 172 and 173. 3,3-Dibromoxindole 215 was prepared in 72% yield by refluxing indoline-2-one 216 and copper(ii) bromide in ethyl acetate for 3 h (Scheme 46).^6^

### 3.4 Synthesis of trisindolines from trimerization reactions of indoles

**Scheme 47:**
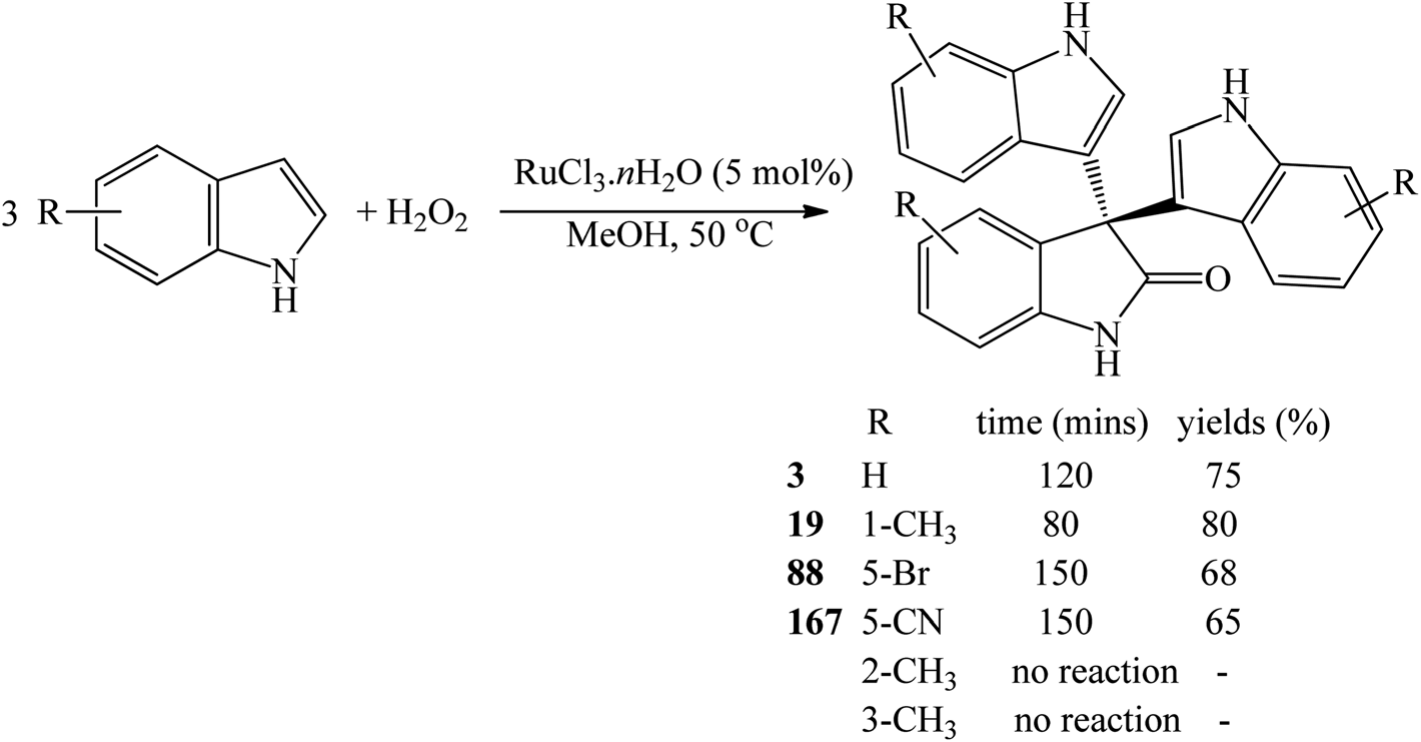
Trimerization of indoles using RuCl_3_·*n*H_2_O.

Tabatabaeian *et al.* (2009) synthesized trisindolines 3, 19, 88 and 167 in 65–80% yields by self-condensation reaction of indoles in the presence of hydrogen peroxide and RuCl_3_·*n*H_2_O (5 mol%) as catalyst in methanol at 50 °C within 80–150 minutes (Scheme 47). Deactivated indoles 5-cyanoindole and 5-bromoindole reacted slower and gave lower yields compared with activated (*N*-methylindole) and unsubstituted indoles. However, 3-methylindole and 2-methylindole were unreactive.^82^

**Scheme 48:**
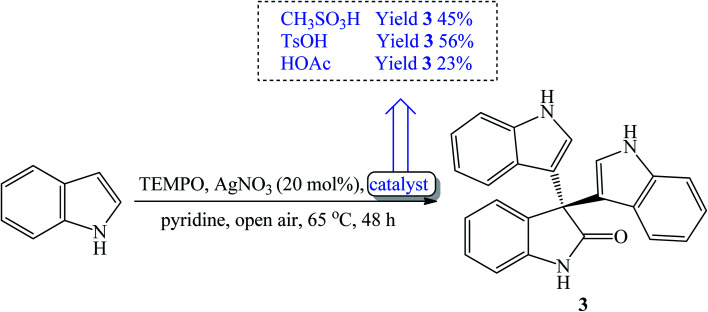
Trimerization of indoles using TEMPO.

Trisindoline 3 was prepared by the reaction of indole in the presence of AgNO_3_ (20 mol%), acid catalyst additive and TEMPO (2,2,6,6-tetramethylpiperidine-1-oxyl radical) as additive, in pyridine under open air at 65 °C for 48 h. For comparison, compound 3 was obtained in 45%, 56%, 23% yields respectively using methanesulfonic acid or *p*-toluenesulfonic acid or acetic acid as catalysts (Scheme 48).^113^

## 4. Bioactivity of trisindolines

### 4.1 Anticancer activity

**Fig. 8:**
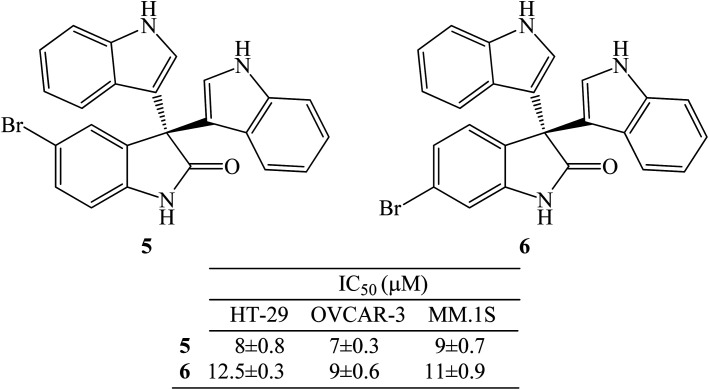
IC_50_ values of 5 and 6 against HT-29, OVCAR-3, and MM.1S cell lines.

Trisindoline 3 was reported to have anticancer activity against colorectal adenocarcinoma (HCT15) and uterine sarcoma (MES-SA). Trisindoline 3 displayed stronger toxicity against both multidrug resistant cell lines HCT15/CL02 and MES-SA/DX5 and exhibited IC_50_ values of 5.11 ± 0.44 μM and 5.50 ± 0.53 μM, respectively, compared to etoposide which is used for treating ovarian and testicular cancers.^9^ Trisindoline 3 displayed IC_50_ of 18.4 μM against A-549 cell lines (lung cancer cells) while its isomer, 2,2-di(3-indolyl)-3-indolone 4, was less potent with IC_50_ value of 69.7 μM.^19^ In other research, trisindoline 3 showed stronger toxicity against A-549 cell lines with IC_50_ values of 8.6 μM^8^ and 1.23 ± 0.030 μM.^7^ Trisindoline 3 was also investigated for its anticancer activity against SK-N-SH cell lines (neuroblastoma) and gave IC_50_ of 11.3 μM^8^ and 0.90 ± 0.06 μM (IC_50_ of doxorubicin as drug standard was 0.97 ± 0.03 μM).^7^ Furthermore, trisindoline 3 exhibited IC_50_ value of 5.70 μg mL^−1^ against HL-60 (human promyelocytic leukemia cell line) and 6.85 μg mL^−1^ against Bel-7402 (human hepatocellular carcinoma cell line), and was more potent than indoles isolated from *Shewanella piezotolerans* (Table 5).^22^ Natural brominated trisindolines 5 and 6 showed promising activity against cancer cell lines HT-29 (human colon), OVCAR-3 (human ovarian), and MM.1S (multiple myeloma) (Fig. 8).^23^

**Table 5:** IC_50_ values of trisindoline 3 against cancer and normal cell lines^a^

Cancer/tumor cell lines	IC_50_			References
	Trisindoline 3	Etoposide (standard)	Doxorubicin (standard)	
HCT-15	6.63 ± 0.43 μM	1.42 ± 0.02 μM		9
HCT-15/CL02	5.11 ± 0.44 μM	11.68 ± 2.38 μM		9
MES-SA	3.51 ± 0.03 μM	0.28 ± 0.02 μM		9
MES-SA/DX5	5.50 ± 0.53 μM	8.14 ± 0.89 μM		9
A549	18.4 μM			19
	8.6 μM			8
	1.23 ± 0.030 μM		15.07 ± 0.13 μM	7
SK-N-SH	11.3 μM			8
	0.90 ± 0.06 μM		0.97 ± 0.03 μM	7
HL-60	5.70 μg mL^−1^			22
Bel-7402	6.85 μg mL^−1^			22
MCF-7	49.8 μM			8
Hep G-2	20.4 μM			8
DU-145	8.7 μM			8
MDA-MB	Inactive		8.14 ± 0.14 μM	7
MRC-5	>100 μM		14.84 ± 0.25 μM	7

a HCT-15: colorectal adenocarcinoma; HCT-15/CL02: multidrug-resistant colorectal adenocarcinoma; MES-SA: uterine sarcoma; MES-SA/DX5: multidrug-resistant uterine sarcoma; A549: lung cancer; SK-N-SH: neuroblastoma or central nerve system (CNS) cancer; HL-60: promyelocytic leukemia; Bel-7402: hepatocellular carcinoma; MCF-7: breast cancer; Hep G-2: liver cancer, DU-145: prostate cancer, MDA-MB: breast cancer; MRC-5: human normal lung.

Kamal *et al.* assayed the bioactivity of trisindolines 3, 15–17, 30, 32, 42, 60, 61, 101, 124 and 144–148 against lung cancer cell lines (A549), central nerve system cancer cell lines (SK-N-SH), breast cancer cell lines (MCF-7), liver cancer cell lines (Hep G-2), and prostate cancer cell lines (DU-145). Trisindolines 16 and 145 with methoxy substituent at C-5 and C-6 showed lower IC_50_ of 2.2 μM and 1.2 μM, respectively, against DU-145. In comparison, trisindoline 15 with Br on C-5 gave IC_50_ value of 3.6 μM. Trisindoline 146 showed high potency with IC_50_ value of 7.5 μM against MCF-7 cancer cells. 5-Fluoro-3,3-di(1*H*-indol-3-yl)indolin-2-one 32 also showed anticancer potency against Hep G-2 while other analogues either showed higher IC_50_ or were inactive. Trisindoline 101 showed the best IC_50_ against SK-N-SH. In addition to its activity against DU-145, trisindoline 145 displayed IC_50_ value of 7.8 μM against A549 cell lines, and this value was similar to the ones exhibited by trisindolines 146 and 124. Overall, the introduction of a methoxy moiety on the indole framework in any position positively enhanced the anticancer activity.^8^

**Fig. 9:**
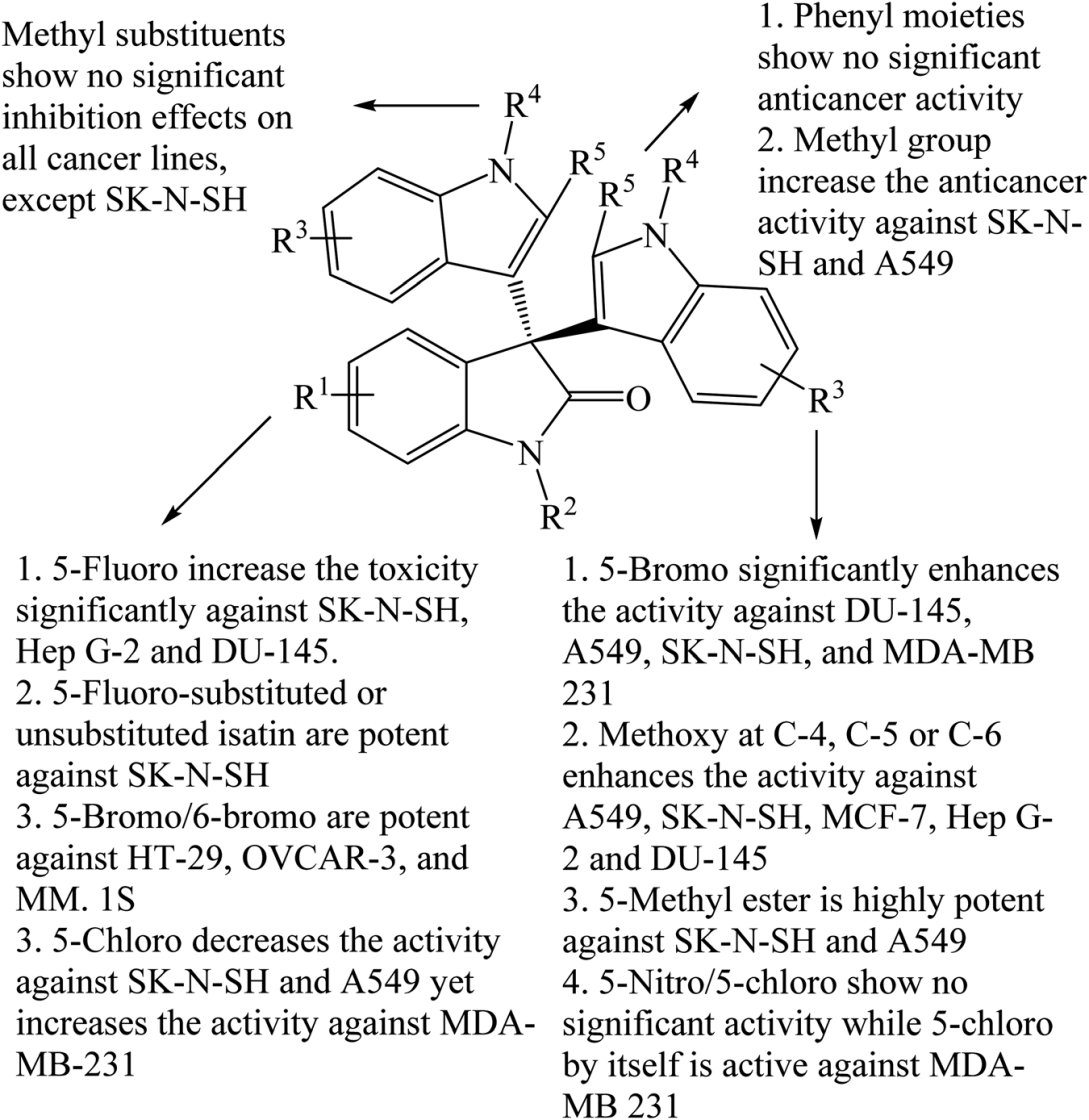
Effect of various substituents on the anticancer activity of trisindolines.

Trisindolines 3, 15, 17, 22, 30, 31, 42, 54, 55, 86 and 106–110 were examined against MDA-MB 231 (breast), SK-N-SH (neuroblastoma), A549 (lung), and MRC-5 (normal human lung cell) cell lines. Trisindolines 31, 86, 108–110 with 5-chloroisatins were not potent against A549 and SK-N-SH. However, the combination of 5-chloroisatin with 5-bromoindole as in trisindoline 86 gave IC_50_ of 1.04 μM against SK-N-SH cancer cell line. Isatins and indoles bearing electron withdrawing group (except 5-chloro) and/or with methyl on C-2 showed promising activity with IC_50_ of 0.75–1.23 μM against lung cancer cells (A549) and 0.90–0.99 μM against neuroblastoma cancer cells (SK-N-SH). In the case of lung cancer, they have significantly lower IC_50_ value compared to doxorubicin with IC_50_ of 15.07 μM. However, doxorubicin possessed IC_50_ of 0.97 μM towards neuroblastoma cancer cell lines. The combinations of isatins and indoles bearing EWG (except CO_2_Me) or trisindolines with EWG (except halogen) on indoles and chloro on isatin, inhibited the viability of MDA-MB 231 cells and exhibited IC_50_ value of 9.95–10.05 μM (IC_50_ of doxorubicin is 8.14 μM). The introduction of 2-phenylindole decreased the activity against three cancer cell lines (MDA-MB 231, SK-N-SH, A549). Trisindolines 15 and 42 were potent during the inhibition of the above cancer cell lines. Interestingly, trisindolines 3, 15, 17, 22, 30, 31, 42, 54, 55, 86 and 106–110 were not toxic to the normal cell (MRC-5) although some were not active as anticancer agents.^7^ A simplified structure–activity relationship (SAR) of trisindolines toward anticancer activity can be seen in Fig. 9.

### 4.2 Antimicrobial activity

Trisindoline 3 showed antibacterial activity against *E. coli*, *B. subtilis*, *S. aureus* at 10 μg per disk with inhibition zone of 16, 17, and 10 mm respectively.^6^ Trisindoline 3, unlike its analogues, possessed better activity against Gram-negative bacteria compared to Gram-positive bacteria. Trisindolines 172 with 5-carboxylic acid indole and 173 with 4-hydroxyindole did not show any inhibitions while trisindoline 15 with 5-bromoindole exhibited weak activity (at the concentration of 30 μg per disk) against *E. coli*.^6^

Trisindoline 3 was found to have excellent antibacterial activity against *B. cereus*, displaying inhibition zone of 20 mm at 10 μg per paper.^18^ The antimicrobial activity of trisindoline 3 and *N*-benzyl-substituted trisindoline 25 was assayed by disc diffusion method using 6 mm paper discs. Compounds 3 and 25 were found to possess inhibition zones of 25 mm and 26 mm against *B. subtilis* respectively, while chloramphenicol and gentamycin (antibiotics) showed inhibition zones of 26 mm and 28 mm respectively. Trisindolines 5, 31 and 27 with halogen or nitro groups on the isatin moiety caused 11–18 mm inhibition zone, while trisindolines 17, 57 and 29 with methyl group on the nitrogen did not show inhibition zones. Compounds that were active against *B. subtilis*, also possessed antimicrobial activity against *S. aureus*, but with lower inhibition zone of 12–16 mm than that of gentamycin with 20 mm inhibition zone. All the substituted trisindolines 3, 5, 17, 25, 27, 29, 31 and 57 were not active against *E. coli* and *P. aeruginosa*.^59^

Trisindolines 14 with 5-bromo and *N*-benzyl moieties on isatin showed promising antibacterial activity against *S. aureus* with inhibition zone of 23 mm and MIC of 1.25 μg mL^−1^. Trisindoline 5 without the *N*-benzyl moiety on isatin and trisindoline 161 with 2-methyl on the indole unit exhibited lower activity with inhibition zones of 12 mm (MIC of 10 μg mL^−1^) and 19 mm (MIC of 2.5 μg mL^−1^), respectively. However, trisindolines 5, 14 and 161 were inactive against *E. coli* and *P. aeruginosa*.^95^

Trisindolines 125–137 inhibited *E. coli* and *S. aureus* successfully. Trisindoline 135 with *N*-benzyl group in the isatin and *N*-propargyl group in indole rings emerged as the most active against *E. coli* with 17 mm inhibition zone as well as the most active against *S. aureus* with 15 mm inhibition zone. Trisindolines 125–134, 136 and 137 showed zone of inhibitions of 10–15 mm against *E. coli* and 10–15 mm against *S. aureus*. Trisindolines 129, 133 and 137 were the most potent with 15 mm inhibition zone against *S. aureus*. Under the same conditions, Amikacin exhibited 18- and 17 mm zone of inhibition for *E. coli* and *S. aureus*, respectively.^10^ The presence of substituents on the nitrogen of isatin or indole seems to contribute positively to the antibacterial activity against *E. coli*.

**Fig. 10:**
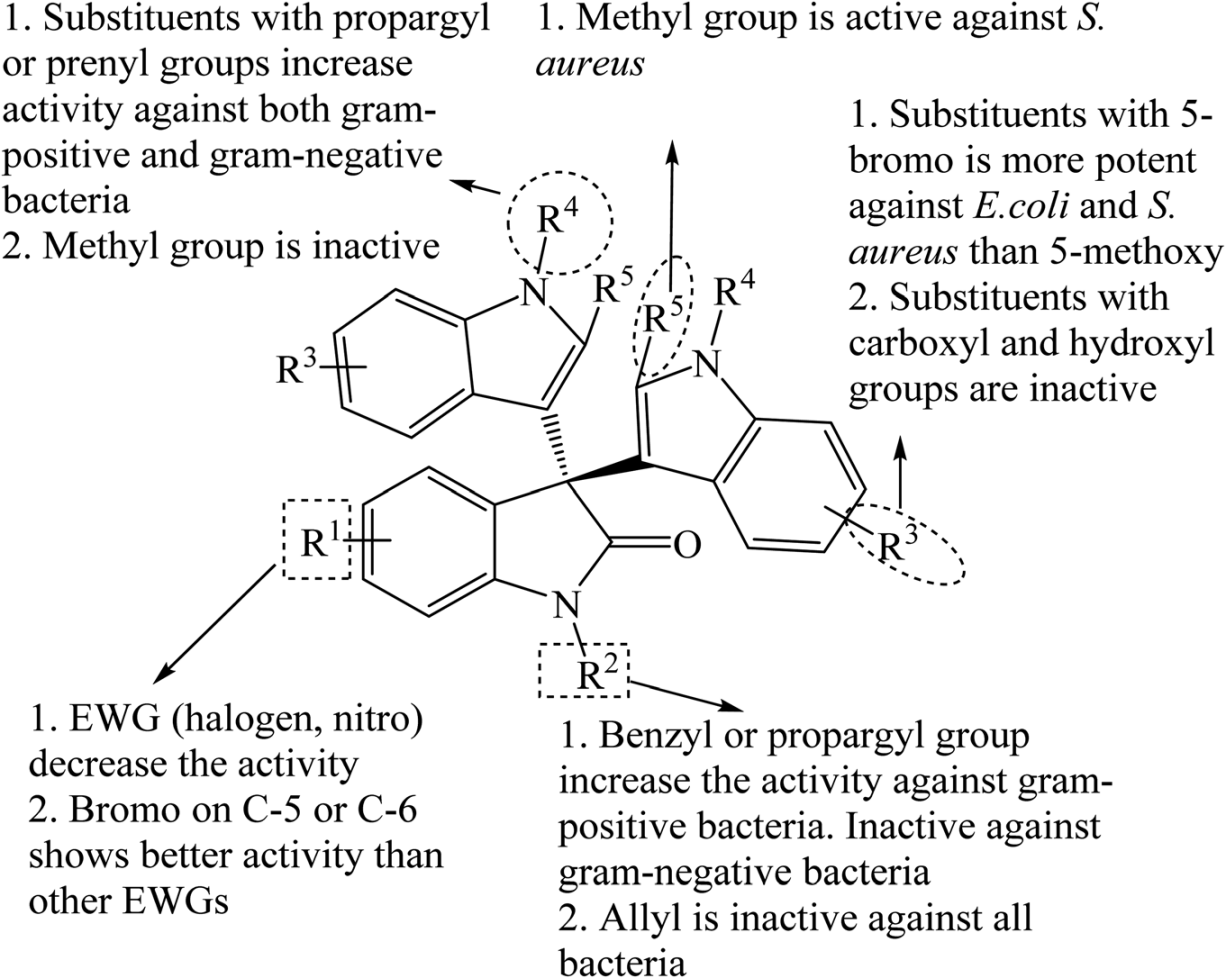
Structure–activity relationship (SAR) of trisindolines as antibacterial agents.

The antibacterial activity of trisindolines 5 and 6 was examined against *S. aureus*, *B. subtilis*, *E. coli*, and *P. aeruginosa* by disc diffusion method (Table 6).^23^ Trisindoline 5 possessed wider inhibition zone against *S. aureus* (17.5 ± 0.8 mm) and *B. subtilis* (18 ± 0.1 mm) than trisindoline 6, and both were weaker than amikacin. However, trisindolines 5 and 6 showed better inhibition against *S. aureus* and *B. subtilis* than gentamicin. Trisindoline 5 with the 5-bromine on isatin is more potent than 6 having 6-bromine. However, both compounds were inactive against Gram-negative bacteria (*E. coli*, and *P. aeruginosa*).^19^ The structure–activity relationship (SAR) of the trisindolines as antibacterial agents is seen in Fig. 10.

**Table 6:** Zone of inhibition and MIC values of 5 and 6 against *S. aureus* and *B. subtilis*

Trisindolines	Zone of inhibition (mm)		MIC (μg mL^−1^)	
	*S. aureus*	*B. subtilis*	*S. aureus*	*B. subtilis*
5	17.5 ± 0.8	18 ± 0.1	8	4
6	15 ± 1.1	16.4 ± 0.9	16	4
Amikacin	23.5 ± 0.8	20.2 ± 0.6	—	—
Gentamycin	—	—	16	8

### 4.3 Antimycobacterial activity

The potency of trisindoline 3 and its analogues 13 and 14 as antitubercular agents was examined using the resazurin microtiter assay (REMA) method.^11^ Trisindoline 3 was inactive (MIC > 25 μg mL^−1^) while trisindolines 13 and 14 inhibited the growth of *Mycobacterium tuberculosis* H_37_Rv with MIC values of 12.5 and <6.25 μg mL^−1^, respectively. Rifampicin reference showed MIC of <6.25 μg mL^−1^ under the same conditions.^11^

### 4.4 Antifungal activity

Trisindolines 3, 5, 17, 25, 27, 29, 31 and 57 were found inactive against *Candida albicans* by the disc diffusion method.^59^ Compounds 5, 14 and 161 were assayed *in vitro* using USP 29-NF25 cylinder plate assay against *Candida albicans*, and none of them were active as antifungal agents.^95^ Surprisingly, trisindolines 125–137 displayed inhibitions against *Candida albicans* by cup plate method. Compounds 126 and 137 showed the best inhibition (15 mm zone of inhibition) while ketonazole as standard antifungal agent showed 16 mm inhibition zone. Other analogues 125 and 127–136 displayed 10–14 mm inhibition zone. The presence of methoxy group on C-5 of the indole increased the activity against *Candida albicans*.^10^ The inactivity of trisindolines to inhibit the growth of *C. albicans* was presumably caused by the absence of substituents on the nitrogen atom in either isatin or indole.

### 4.5 Anticonvulsant activity

A series of trisindolines 125–137 were tested *in vivo* to examine their anticonvulsant activity by observing seizure in rat.^10^ All the tested compounds 125–137 were active at a dose of 20 mg kg^−1^ and halted convulsion. Compounds 127, 128, 130, 133, 136, 137 markedly showed excellent activities with extensor time of 31.60–31.80 s while standard drug phenytoin's extensor time was 43.00 ± 0.40 s (Table 7). The presence of *N*-propargyl on indole or isatin moieties as well as *N*-allyl moiety reduced the time of tonic extensor remarkably. Methyl substituent on C-2 of the indole increased the activity. Trisindoline 125 was less promising as anticonvulsant since it possessed longer extensor time than phenytoin.^10^

**Table 7:** The effect of trisindolines 125–137 on seizures induced by MES in mice

Trisindolines	R^1^	R^2^	R^3^	R^4^	Time (s)		
					Flexion	Extensor	Clonus
125	H	H	H	Allyl	5.80 ± 0.20	48.00 ± 0.32	3.60 ± 0.20
126	5-NO_2_	H	H	Allyl	4.80 ± 0.20	39.20 ± 0.40	3.40 ± 0.24
127	H	H	5-OMe	Allyl	4.80 ± 0.18	31.80 ± 0.40	3.20 ± 0.24
128	5-Cl	H	5-OMe	Allyl	5.80 ± 0.20	31.60 ± 0.37	3.40 ± 0.20
129	5-Br	H	5-OMe	Allyl	4.80 ± 0.20	39.20 ± 0.40	3.40 ± 0.24
130	H	CH_2_CO_2_Et	5-OMe	Allyl	4.80 ± 0.20	31.60 ± 0.37	3.40 ± 0.24
131	H	H	H	Cinnamyl	4.80 ± 0.20	39.60 ± 0.40	3.60 ± 0.20
132	H	H	5-Br	Prenyl	4.80 ± 0.20	39.60 ± 0.40	3.40 ± 0.24
133	H	Propargyl	5-Br	Prenyl	5.40 ± 0.24	31.60 ± 0.37	2.80 ± 0.24
134	H	H	H	Propargyl	4.80 ± 0.20	39.20 ± 0.40	3.20 ± 0.24
135	H	Bn	H	Propargyl	4.80 ± 0.18	39.20 ± 0.40	3.20 ± 0.24
136	H	Propargyl	H	Propargyl	5.40 ± 0.20	31.80 ± 0.32	3.60 ± 0.20
137	H	H	2-Me	Propargyl	5.40 ± 0.24	31.60 ± 0.37	2.80 ± 0.24
Phenytoin (reference standard)					5.60 ± 0.20	43.00 ± 0.40	3.40 ± 0.24

### 4.6 α-Glucosidase inhibition activity

**Fig. 11:**
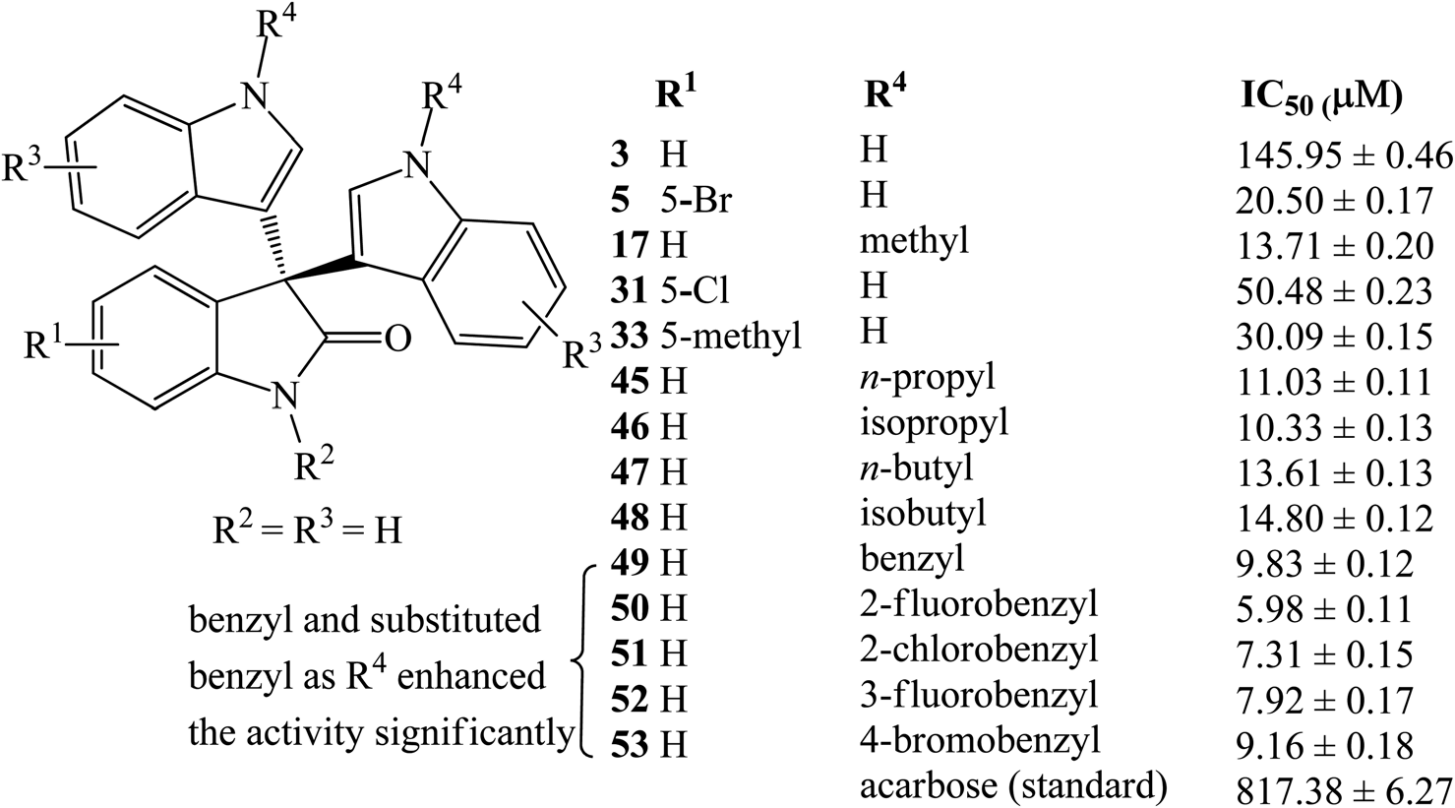
IC_50_ values of trisindolines 3, 5, 17, 31, 33 and 45–53 as α-glucosidase inhibitors.

Trisindolines 3, 5, 17, 31, 33 and 45–53 inhibited α-glucosidase activities and possessed significantly lower IC_50_ values than commercial drug acarbose (Fig. 11).^12^ The presence of bromo substituent on isatin ring enhanced the activity. Trisindoles with *N*-benzyl and *N*-substituted benzyl isatins were more active than *N*-alkyl analogues. Trisindoline 3 displayed the lowest inhibition activity and yet was superior to acarbose (Fig. 11).

### 4.7 Spermicidal activity

Trisindoline 3 and its analogues 15–17, 22 and 105 were investigated for their effects on sperm mobility (Table 8). The minimum effective concentrations (MECs) were defined as the minimum concentration that led 100% sperm immobility within 20 seconds without awakening the next motility in Baker's buffer after 1 h of incubation at 37 °C. Trisindoline 16 showed the best inhibition activity with MEC of 0.34 ± 0.018 mg mL^−1^. In comparison, nonoxynol-9 (N-9) as standard showed lower MEC of ∼0.5.^13^

**Table 8:** MEC values of trisindolines 3, 15–17, 22 and 105 as spermicidal

Trisindolines	MEC (mg mL^−1^)	Trisindolines	MEC (mg mL^−1^)
3	2.40 ± 0.058	22	2.82 ± 0.057
15	3.18 ± 0.041	16	0.34 ± 0.018
17	2.36 ± 0.045	105	1.11 ± 0.061

### 4.8 Miscellaneous activities

Compound 3 displayed moderate inhibition activity against xanthine oxidase with IC_50_ of 179.6 ± 0.04 μg mL^−1^ while standard inhibitor allopurinol showed IC_50_ value of 7.4 ± 0.07 μg mL^−1^.^25^ Xanthine oxidase is an enzyme responsible for catalyzing the transformation of xanthine to uric acid as well as reducing oxygen (O_2_) into reactive oxygen species (ROS). The abundance of uric acid causes oxidized lipid membrane, leading to hyperuricemia and obesity.^114^

Trisindoline 3 showed promising bioactivity as tyrosinase inhibitor with IC_50_ value of 17.34 ± 0.04 μg mL^−1^ while reference l-mimosine showed IC_50_ value of 37.0 ± 0.03 μg mL^−1^.^25^ Tyrosinase enzyme is critical to melanogenesis as it catalyzes the production of melanin in skin, hair, and eye pigmentation.^115^

Trisindoline 3 was less potent as antioxidant since it showed IC_50_ value of 431 ± 0.09 μg mL^−1^ compared with the positive control of 3-*tert*-butyl-4-hydroxyanisole (BHA) with IC_50_ value of 46 ± 0.22 μg mL^−1^.^25^

## 5. Future directions

Various routes and catalysts have been explored for trisindoline synthesis. In majority of cases, acids have been used to catalyze the reaction successfully. Ionic liquid-catalyzed condensation reaction of isatins with indoles have attracted attention since it is considerably environmental benign and requires simple operation giving high yields within short reaction time. Besides, many ionic liquid catalysts could be utilized to synthesize both symmetrical and unsymmetrical trisindolines. On other hand, only a handful of trisindolines have been synthesized using ionic liquids which warrants further exploration. Trisindolines emerge as a new class of bioactive compounds as it displays several promising bioactivities that need developing. However, limited analogues of trisindoline have been well-investigated and the structure activity relationship is reported on limited varieties of compounds especially in when trisindolines were explored as antitubercular and α-glucosidase inhibitors. Interestingly, there is also very limited studies on the biological activity of unsymmetrical trisindolines which should be explored.

## 6. Conclusions

Trisindolines are nitrogen heterocyclic structures with promising biological activities. This review provides comprehensive account of trisindolines including their natural occurrence, synthesis, and biological activities. Various routes of synthesis and catalysts used have been discussed in detail. The biological activities of trisindolines have also been discussed with a special focus on the structure activity relationship. This review aims to inspire further development of trisindolines as lead drug candidates.

## Conflicts of interest

There are no conflicts to declare.

## Supplementary Material
